# Hypoxia deactivates epigenetic feedbacks via enzyme-derived clicking proteolysis-targeting chimeras

**DOI:** 10.1126/sciadv.abq2216

**Published:** 2022-12-14

**Authors:** Thang Cong Do, Jun Wei Lau, Caixia Sun, Songhan Liu, Khoa Tuan Kha, Seok Ting Lim, Yu Yang Oon, Yuet Ping Kwan, Jia Jia Ma, Yuguang Mu, Xiaogang Liu, Thomas James Carney, Xiaomeng Wang, Bengang Xing

**Affiliations:** ^1^ Division of Chemistry and Biological Chemistry, School of Physical and Mathematical Sciences, Nanyang Technological University, Singapore 637371, Singapore.; ^2^ Department of Chemistry, National University of Singapore, Singapore 117543, Singapore.; ^3^ Duke-NUS Medical School, Singapore 169857, Singapore.; ^4^ Singapore Eye Research Institute, Singapore 169856, Singapore.; ^5^ School of Biological Sciences, Nanyang Technological University, Singapore 637551, Singapore.; ^6^ Lee Kong Chian School of Medicine, Nanyang Technological University, Singapore 636921, Singapore.; ^7^ Institute of Molecular and Cell Biology, A*STAR, Singapore 138673, Singapore.; ^8^ School of Chemistry, Chemical Engineering and Biotechnology, Nanyang Technological University, Singapore 637371, Singapore.

## Abstract

Epigenetic mediation through bromodomain and extraterminal (BET) proteins have progressively translated protein imbalance into effective cancer treatment. Perturbation of druggable BET proteins through proteolysis-targeting chimeras (PROTACs) has recently contributed to the discovery of effective therapeutics. Unfortunately, precise and microenvironment-activatable BET protein degradation content with promising tumor selectivity and pharmacological suitability remains elusive. Here, we present an enzyme-derived clicking PROTACs (ENCTACs) capable of orthogonally cross-linking two disparate small-molecule warhead ligands that recognize BET bromodomain-containing protein 4 (BRD4) protein and E3 ligase within tumors only upon hypoxia-induced activation of nitroreductase enzyme. This localized formation of heterobifunctional degraders promotes specific down-regulation of BRD4, which subsequently alters expression of epigenetic targets and, therefore, allows precise modulation of hypoxic signaling in live cells, zebrafish, and living mice with solid tumors. Our activation-feedback system demonstrates compelling superiorities and may enable the PROTAC technology with more flexible practicality and druggable potency for precision medicine in the near future.

## Teaser

An enzyme-derived clicking proteolysis targeting chimera degrades BRD4 specifically at the hypoxia settings within tumours.

## INTRODUCTION

Hypoxia, the state of low oxygen level, is a common feature of most tumors and frequently represents the pernicious effects of cancer progression, therapeutic resistance, uncontrolled metastasis, and poor prognosis (*1*–*3*). The cellular response to oxygen deficiency is mainly mediated by hypoxia-inducible factors (HIFs), a family of crucial transcription factors orchestrating the expression of various essential genes involved in the manipulation of epigenetic plasticity and the acquisition of cancer hallmarks to adapt to hostile environment in tumors (*4*, *5*). Apart from directly affecting gene expression, these HIFs and the hypoxic tumor microenvironment can also influence epigenetic modifications (*6*–*8*). A clear interpretation of the intrinsic correlation between hypoxia and epigenetic regulations is critical to identify plausible cancer hallmarks for effective therapeutic developments. However, much detail remains to be further clarified.

Bromodomain-containing protein 4 (BRD4), an important member of the bromodomain and extraterminal (BET) family, can recognize acetylated histones and recruit transcription factors as well as epigenetic mediators to regulate gene replication and transcription (*9*–*12*). Dysfunction of BRD4 protein is closely associated with the malignant progression of various tumors and, thus, represents a promising target for cancer treatment (*13*–*15*). So far, inhibition or degradation of BRD4 has been found to effectively constrain malignant progression and distant metastasis of tumors (*16*–*22*). Among the different strategies, proteolysis-targeting chimeras, known as PROTACs, are typical heterobifunctional molecules consisting of a ligand specific for an intracellular protein of interest (POI) associated via an appropriate linker with an E3 ubiquitin ligase recruiting moiety that triggers ubiquitination and subsequent degradation of target proteins in the proteasome (*23*–*26*). Unlike traditional drug design through occupancy-driven mechanism such as antibody or small-molecule inhibitors, the PROTAC technology exhibits great pharmacological merits with less susceptibility to point mutations in target proteins. In theory, this ubiquitin-mediated protein degradation can abrogate or manipulate the expression of all disease-modifying molecules, including those that are difficult to target with traditional pharmacological approaches. The maximized drug potency can be fully potentiated for addressing multifunctional drugs or targets (*27*, *28*).

However, the development of PROTACs requires an extensive investigation of the physicochemical properties of POI, the E3 ligase ligand, and the linkers, by evaluating the prerequisite architecture and structure-activity relationship. Disparity or alteration of any of these three elements can lead to substantial diminution in activity (*29*–*32*). As both ends of PROTACs are connected via a linker, its exact nature of high molecular weight should be carefully considered to ensure optimal absorption, distribution, metabolism, excretion, and toxicity profile (*33*–*38*). In addition, intracellular accumulation of heterobifunctional molecules needs to be monitored to minimize off-target response. This can be achieved by controlling cell-type selectivity of the compounds or by assessing the appropriate tissue or disease-specific E3 ligases (*39*, *40*). To comprehend and resolve target specificity, the incorporation of affinity ligands or reporters into the conventional architecture of PROTACs is gradually being developed. However, this adds complexity to the compounds that might hinder their application in clinical translation (*41*–*45*). A more comprehensive design consideration is required to overcome the limitation of current development aspects to bring further therapeutic benefits.

**Fig. 1.**
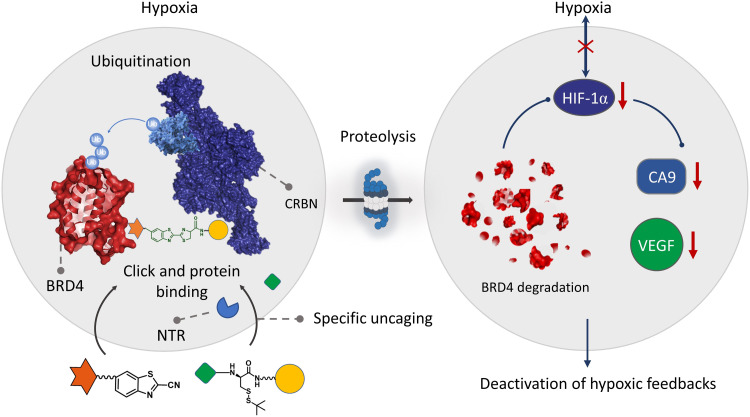
Illustration of ENCTAC performances under hypoxic condition for selective degradation of BRD4 proteins.

In this work, we developed a system that offers enzyme-derived clicking PROTACs (ENCTACs) for stimulation-responsive protein degradations in the hypoxic microenvironment (Fig. 1). Typically, this degradation paradigm relies on the nitroreductase (NTR) enzyme for selective formation of heterobifunctional degraders only at the hypoxic site, promoting targeted epigenetic BRD4 degradation in vitro and in vivo. The ENCTACs are initialized by NTR reduction of a nitro-containing substrate to expose the cysteine fragment premodified with an E3 ligase cereblon (CRBN) recruiting moiety, pomalidomide. Subsequently, orthogonally cross-linking (*46*) occurs between the uncaged cysteine residue and the 2-cyanobenzothiazole (CBT) preconjugated BRD4 ligand, JQ1, to produce the localized degrader molecule. This first-in-class strategy realizes enzyme-selective protein disruption without the need for tedious chemical synthesis to link two protein recruiters beforehand. Moreover, these small-molecule ENCTACs exhibited favorable pharmacokinetic profile and potent tumor inhibition efficacy in vivo, which may offer PROTAC technology with more flexible practicality and druggable potency for the precision medicine in the future.

## RESULTS

### Rational design and formations of our NTR-activatable ENCTACs

**Fig. 2.**
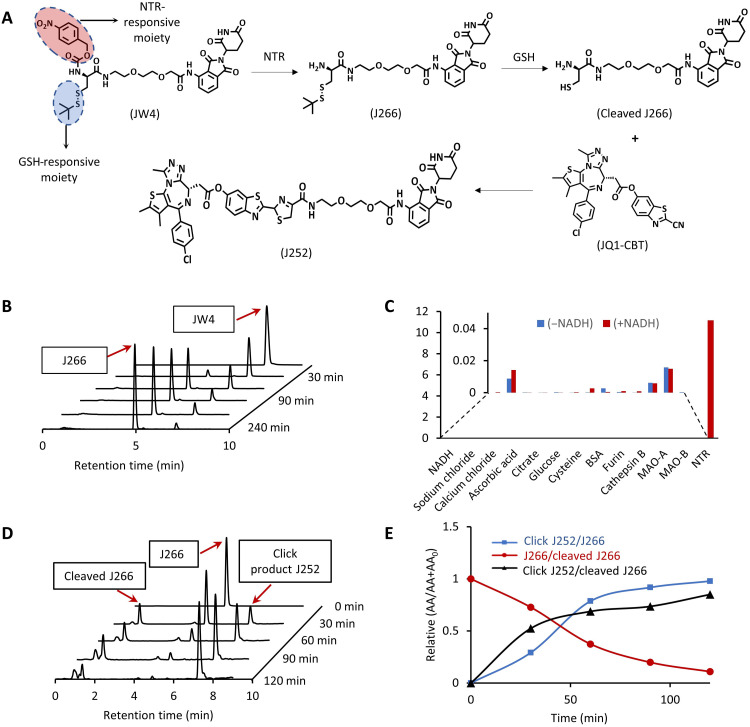
Enzyme-derived click formation of heterobifunctional degraders of BRD4. (**A**) Chemical structure of GSH-responsive CRBN ligand (J266); GSH- and NTR-responsive CRBN ligand (JW4); cleaved J266; CBT-linked BRD4-targeting ligand (JQ1-CBT); click-induced BRD4 degrader (J252). (**B**) LC-MS spectra of NTR uncaging JW4 (10 μM) to form J266 at different time points in NTR (40 μg/ml) dissolved in phosphate-buffered saline (PBS; 10 mM) (pH 7.4). (**C**) Selectivity of JW4 toward a broad range of biological and chemical agents with or without NADH. (**D**) Time-dependent LC-MS spectra of click J252 formation under reducing reagent [tris(2-carboxyethyl)phosphine (TCEP)] that cleave the J266 to induce cleaved J266 before the click reaction with JQ1-CBT in buffer solution. (**E**) Ratio of peak areas of click J252 and cleaved J266 to J266 over multiple time points in (D).

To achieve an NTR-oriented ENCTACs, we proposed the rational design and molecular structures of different components, J266, JW4, JQ1-CBT, and J252, in Figs. 1 and 2A. In general, the synthetic design is separated into two different parts including the E3 ligase–recruiting ligand pomalidomide (circled in blue) and the BRD4-targeting ligand JQ1 (circled in red). For the pomalidomide-linked fragment, J266 was first synthesized by covalent coupling between pomalidomide-PEG_2_-NH_2_ and Boc-Cys(StBu)-OH. The thiol *tert*-butyl(–StBu) group serves as a glutathione (GSH)–responsive moiety (Fig. 2A). Subsequently, the Boc-protected amino group was deprotected and further caged with *p*-nitrobenzyl chloroformate to obtain JW4, the molecular fragment that can correspond to both NTR-responsive uncaging and reductive GSH cleavage. On the other hand, the BRD4-targeting ligand, JQ1, underwent esterification with CBT to form JQ1-CBT (fig. S12). Last, J252 was also synthesized as our preformed PROTAC control molecule to perform a preliminary analysis of the linker properties on the ENCTAC efficacy in targeted protein degradation. All the synthesized molecules were characterized by nuclear magnetic resonance and mass spectrometry (MS; see the Supplementary Materials).

Upon exposure to external enzymatic stimuli, the NTR-responsive moiety undergoes self-immolation to unleash the amino group. Simultaneously, the presence of a reducing agent cleaves the –StBu group to liberate thiol group (-SH), creating the cleaved J266. The presence of a readily reactive amino group and a thiol group on cleaved J266, with the coinsertion of JQ1-CBT would spontaneously induce the orthogonal CBT-cysteine click conjugation and shape the luciferin-based structure into click J252 for targeted protein disruption (Fig. 2A). To validate the kinetics of ENCTAC formation, we tested the as-synthesized JW4 in the presence of NTR enzyme and the cofactor reduced form of nicotinamide adenine dinucleotide (NADH) at 37°C across a range of time settings in the liquid chromatography–MS (LC-MS) system. Over a span of 4 hours under the enzymatic incubation of NTR, JW4 molecules were facilely self-immolated to J266 (Fig. 2B and fig. S1, A to D) with enzyme kinetics of *K*_M_ = 101.29 μM and *K*_cat_ = 0.0157 min^−1^. Experimental results were also repeated for J268 with a disparate linker (C3-NH_2_ instead of PEG_2_-NH_2_) and showed a similar trend of enzymatic cleavage (fig. S1, E to H). These results reveal the flexibility of choosing linker length and hydrophilicity in our NTR-responsive ENCTAC system. Moreover, to identify the specificity of NTR, a selectivity assay of JW4 against different components and biomolecules was performed, and JW4 showed no obvious cleavage except for NTR + NADH (Fig. 2C).

Furthermore, the formation of J252 in the presence of J266 and JQ1-CBT was also examined by LC-MS analysis (Fig. 2, D and E). Upon reduction of J266 by tris(2-carboxyethyl)phosphine (TCEP), CBT-cysteine click formation occurred almost immediately after –StBu cleavage of J266. The relative peak intensity of cleaved J266 remains static, but the peak intensity of click J252 gradually increases, indicating the spontaneity and efficiency of click conjugation. The continuous enzymatic cleavage and click formation process were evaluated in one-pot reaction (fig. S2), signifying the facile formation of the heterobifunctional degrader under fixed enzymatic conditions when sufficient ENCTAC fragments are introduced.

### Luciferin-based heterobifunctional molecules induce BRD4 degradation

**Fig. 3.**
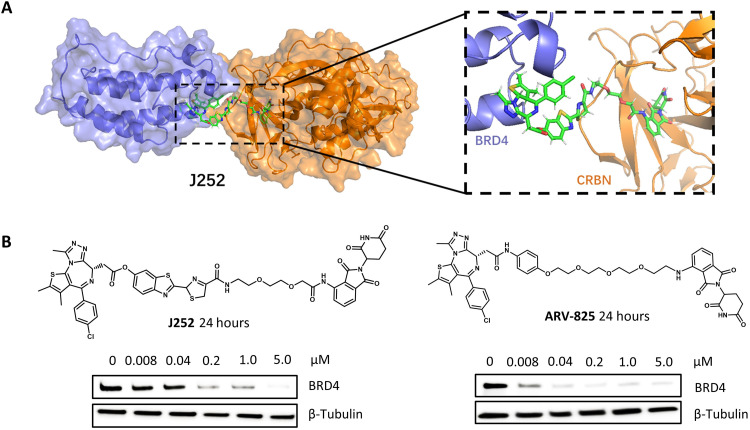
Heterobifunctional degraders efficiently disrupt BRD4 protein. (**A**) Molecular dynamic simulation of J252 interacting with CRBN (gold) and BRD4 (blue) proteins. The position of the luciferin-based component shows no steric collision with BRD4 or CRBN. (snapshot at 1 ns). (**B**) Western blot analysis of BRD4 protein after treatment with J252 and ARV-825 in human embryonic kidney (HEK) 293T cells for 24 hours with indicated concentration and β-tubulin as internal controls.

Inspired by the promising results in hypoxia NTR enzyme–responsive ENCTAC conjugation, we further proceed molecular docking of click J252 based on the crystal structures of BRD4 and CRBN (*21*) and assess the suitability of the orthogonal cross-linking products for the degradation of the POI. Simulation calculations revealed the minimal steric hindrance in the pocket binding site of the two proteins with the luciferin-type linkers. In this context, the difference in linker lengths as indicated in the chemical structures of the presynthesized J252 and analogs also shows that the steric hindrances between the protein-ligand interaction are trivial (Fig. 3A and fig. S3, A and B). In addition, the nonhindered effect of the click product linkers on PROTAC activity was further validated by the analysis of BRD4 protein levels upon treatment with these molecularly simulated compounds. Immunoblot analysis indicated that incubation of the PROTAC compounds for 24 hours induced concentration-dependent degradation of BRD4 (Fig. 3B). The protein depletion effects were comparable to the standard PROTAC ARV-825 degrader in the same cells (*47*). This calculated evidence provides an insightful reference for our study, in which we sought to use the ENCTACs for selective degradation of the BRD4 under the hypoxia condition.

### ENCTACs facilitate hypoxia-specific degradation of BRD4

**Fig. 4.**
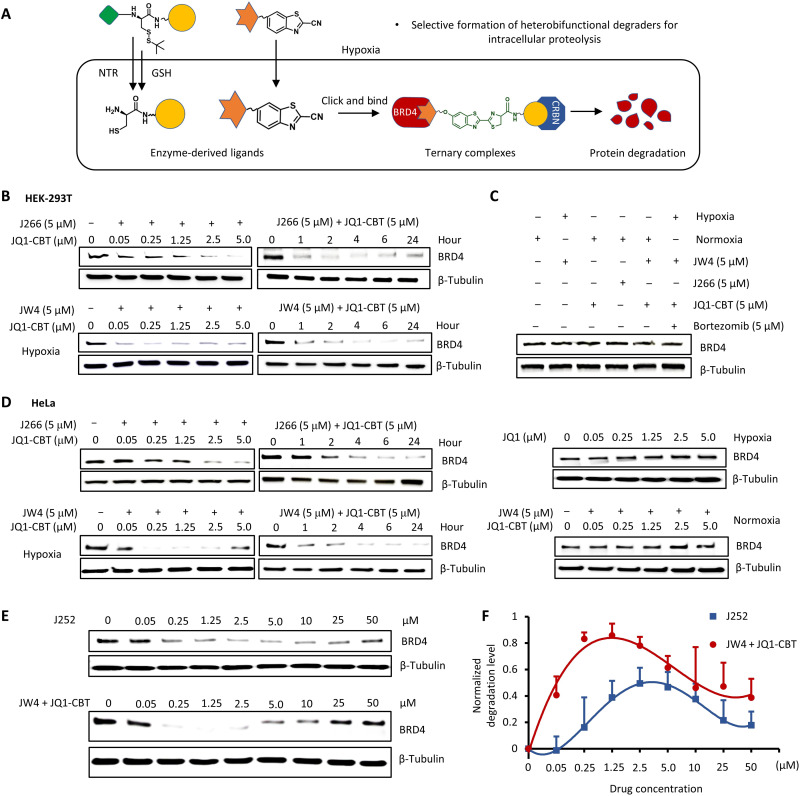
Hypoxia-activated degradation of epigenetic BRD4 protein. (**A**) Illustration of intracellular hypoxic enzyme uncaging, formation of the degrader (click J252), and degradation of BRD4. (**B**) Western blot analysis of BRD4 protein levels after treatments with GSH-cleavable J266 (6 hours) and JQ1-CBT (12 hours) at indicated concentration and different time points; GSH- and NTR-responsive JW4 (6 hours) with JQ1-CBT (12 hours) at the indicated concentration and for a different time period under hypoxia in HEK-293T cells. (**C**) Analysis of BRD4 protein levels under the combination of different control conditions for 12 hours (the inhibitor bortezomib incubated with cells for 2 hours before the addition of JW4 and JQ1-CBT). (**D**) Western blot analysis of BRD4 protein levels after treatments with J266 (6 hours) and JQ1-CBT (12 hours) or JW4 (6 hours) and JQ1-CBT (12 hours) in time- and concentration-dependent examinations of HeLa cancer cells; analysis of BRD4 protein levels after treatments with JQ1 (12 hours) in a concentration-dependent manner of hypoxia HeLa cells; and JW4 (6 hours) and JQ1-CBT (12 hours) in normoxia HeLa cells. (**E**) Extended concentration of degraders demonstrating the hook effect during incubation under hypoxia (representative results from two to three biological replications). β-Tubulin was used as internal controls. (**F**) BRD4 degradation level over varied concentration as indicated in (E). Values represent triplicate means ± SD, normalized to nontreated cells, and baseline-corrected using immunoblots.

The effective production of heterobifunctional BRD4 degraders in solution allowed us to investigate the function of ENCTAC components in biological applications. Considering the up-regulated NTR expression in oxygen-deficient cells, the hypoxia condition was created by 1% O_2_ gas environment with simultaneous incubation of ENCTAC fragments (JW4 and JQ1-CBT). In particular, the NTR-responsive JW4, the pomalidomide containing compound targeting CRBN, is expected to be uncaged through NTR-assisted nitro and GSH reduction once entering hypoxic cells. The exposed 1,2-aminothiol in cysteine groups is then readily linked with the CBT moieties on the JQ1-CBT fragments via a specific bio-orthogonal click reaction in which the JQ1 warheads would be presumed to recognize the BRD4 proteins to form heterobifunctional degraders in situ for localized proteolysis (Fig. 4A).

We next sought to evaluate the viability of the method in selective proteolysis. As a proof of concept, J266, the control molecule that can only respond to GSH but not to NTR, was first introduced into the human embryonic kidney (HEK) 293T cell line (for 6 hours), with the subsequent addition of JQ1-CBT (for 12 hours). After intracellular formation of the click J252, the proteolysis process was observed by Western blot analysis. Figure 4B shows that protein degradation occurred as a function of concentration and time. These results from this exclusively GSH-responsive uncaging molecule of J266 illustrated the possibility of our ENCTACs to facilitate proteolysis after conjugation of two separated fragments. We further examined the hypothesis of cell selectivity activation of small molecule–induced protein degradation using the NTR-responsive compound (JW4). BRD4 protein level in HEK-293T cells exposed to the hypoxic condition showed a substantial reduction with increasing concentration of ENCTAC compounds. Similar to J266, the proteolysis process occurred at an early stage after the introduction of JW4 and JQ1-CBT. After 24 hours, most of BRD4 was degraded in hypoxic HEK-293T cells following the treatment with 5 μM of the compounds. On the contrary, cells treated with JW4, JQ1-CBT, and J266 alone for 12 hours did not induce any degradation of the BRD4 (Fig. 4C). Notably, the pretreatment of cells with a proteasome inhibitor (e.g., bortezomib) before addition of the NTR-activated ENCTACs showed trivial BRD4 degradation. This interference demonstrated the involvement of proteasomal machinery in our ENCTAC system.

The enzyme activation-feedback epigenetic BRD4 protein degradation was further examined in different tumor cell lines including HeLa, MDA-MD-231, 4T1, and B16F10. Immunoblots revealed an effective decrease in epigenetic protein levels compared with the untreated cells in the hypoxic environment with molecule concentration and time dependence (Fig. 4D and figs. S4 and S5). In contrast, treatment of hypoxic cells with the fragmented ENCTAC molecules alone did not implicate degradation of the targeted protein. A similar result was also observed in HeLa cells treated with the BRD4 inhibitor, (+)-JQ1, under the hypoxic condition. Moreover, although the treatment of hypoxic HeLa cells with standard ARV-825 degrader would lead to effective concentration-dependent BRD4 elimination, same protein degradation effect could also be observed in HeLa cells under normoxia conditions (fig. S4). There is no selectivity observed from the different environmental stimulation. In contrast, coincubation of the ENCTAC molecules did not trigger the protein degradation in multiple cell lines in the normoxia environment (fig. S4). This observation indicates that copresence of both fragments is required for activation of the target degradation processes and that NTR-activated ENCTACs are indeed specific for hypoxic cells.

In conventional PROTAC systems, “hook effect” frequently present when heterobifunctional degraders are supplied in excess (*48*). This effect is expected to occur naturally in all ternary complexes in which three components are combined to induce activities (*49*). In conventional PROTAC systems, a saturated amount of the degraders may lead to an excess of binary complexes, minimizing the amount of active ternary complexes and degradation of the target protein. Our presynthesized J252 compound showed the same hook effect as the conventional PROTACs when a high concentration of the compound was used (Fig. 4E). In this case, the decrease in the efficiency of proteolysis started at 5 μM J252 and was markedly interfered by 50 μM of the compound with a degradation level of only around 20%. In our ENCTAC system, with the NTR-responsive JW4 and JQ1-CBT, this effect was also observed, which suggests that the ternary complexes formation would be required via the recruitment of E3 ligase to BRD4 by the click reaction. However, the proteolysis process was obtained at lower concentrations of the ENCTACs but higher efficiency of protein degradation compared with the whole-molecule J252 treatment, possibly due to the better intracellular uptake of the compounds (Fig. 4F). Although the hook effect occurs, the lower concentration of our ENCTACs offers a promising opportunity to enhance the application of targeted protein degradation by small molecules, in which lower molecular weight, simpler chemical preparation, and better cellular penetration of the compound with enhanced site specificity and pharmacological performance could be achieved.

### ENCTACs deactivate the hypoxia response by suppressing HIF-1α

**Fig. 5.**
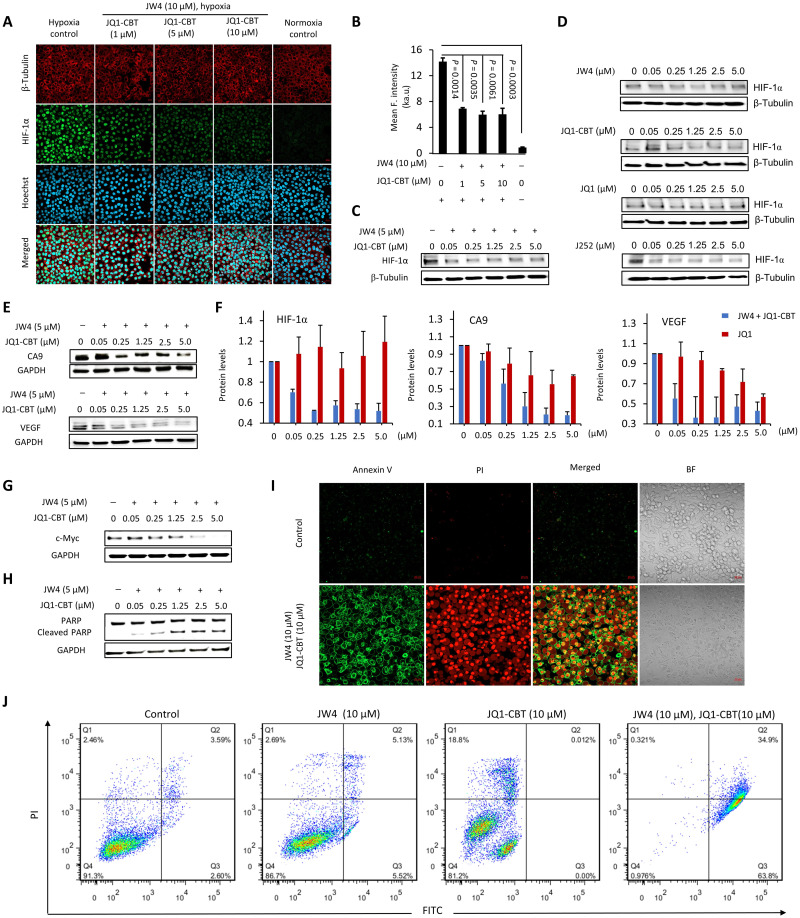
Hypoxic BRD4 degradation resulted in a change in the response of the cellular microenvironment and malfunction of cell growth. (**A**) Confocal imaging of HIF-1α immunostaining (green; λ_ex_ = 488 nm and λ_em_ = 515/30 nm) after subjecting HeLa cells to the as-stated treatment. Nucleus was stained with Hoechst 33258 (blue; λ_ex_ = 405 nm and λ_em_ = 460/50 nm), β-tubulin was stained with the fluorescent β-tubulin antibody (red; λ_ex_ = 561 nm and λ_em_ = 617/20 nm). Scale bars, 40 μm. (**B**) Quantitative mean fluorescence intensity of HIF-1α after treatment and staining as indicated in (A). ka.u., kilo–arbitrary units. Values represent mean fluorescence intensity of three different cell areas ± SD. Western blot analysis of HIF-1α after treatment with different concentrations of JW4 and JQ1-CBT (**C**) or separated JW4, JQ1-CBT, JQ1, and J252 controls (12 hours) (**D**). (**E**) Western blot analysis of vascular endothelial growth factor (VEGF) and CA9 after hypoxia-activated ENCTAC treatment (12 hours). (**F**) Protein level of HIF-1α, VEGF, and CA9 after hypoxia ENCTAC (blue bar) and JQ1 inhibitor treatment (12 hours) (red bar). Values represent the average of duplicates and the range as error bars, normalized to nontreated cells, and baseline-corrected using immunoblots. (**G**) Immunoblot for c-Myc and glyceraldehyde-3-phosphate dehydrogenase (GAPDH) levels after hypoxia-activated ENCTAC treatment using JW4 and JQ1-CBT for 12 hours. (**H**) Immunoblot for poly(adenosine 5′-diphosphate–ribose) polymerase (PARP) cleavage and GAPDH levels after similar treatment condition as in (G). (**I**) Confocal imaging of apoptosis cell death staining with annexin V (AnnV)/propodium iodide (PI) [AnnV (green), λ_ex_ = 488 nm and λ_em_ = 520/30 nm; PI (red), λ_ex_ = 561 nm and λ_em_ = 590/30 nm]. Hypoxic cells without ENCTAC treatment as control. BF, bright field. Scale bars, 40 μm. (**J**) Flow cytometry of apoptosis/necrosis-stained HeLa cells under treatments with JW4, JQ1-CBT individually, or in combination of JW4 and JQ1-CBT (10 μM) (12 hours). Quarter 1 (Q1) indicates the relative percentage of necrosis cells, Q2 indicates late apoptosis cells, Q3 indicates early apoptosis cells, and Q4 indicates live cells.

Epigenetic regulators are essential for HIF-mediated transactivations. In particular, BRD4 is one of the epigenetic readers recruited by zinc finger Myeloid, Nervy and DEAF-1–type containing 8 to the hypoxia-responsive elements of HIF target genes. This protein interacts with positive transcription elongation factor b and supports HIF activation by mediating the release of paused RNA polymerase II and elongation of HIF target genes (*8*, *10*, *50*). So far, the first-generation BET bromodomain inhibitors that are JQ1-related compounds have been tested in the clinic but have shown relatively low potency (*51*) and controversial mechanism to suppress HIF-1α albeit the reduced expression of hypoxia-regulated genes (*17*, *20*), probably due to the complex interplay in the HIF pathway. In contrast, our NTR-activated ENCTAC system can selectively degrade BRD4 under the hypoxia condition, thereby greatly reducing its functions and interactions with other off-target cell proteins. The diminished activity of these epigenetic proteins has a direct consequence in attenuating the expression of HIF-1α, as indicated in Fig. 5 (A and B). Immunofluorescence staining was carried out, and the microscopy images illustrated the nucleus localization of HIF-1α during hypoxia, which is associated with the activation of hypoxia genes. The level of this protein was reduced upon treatment with our ENCTAC compounds (JW4 and JQ1-CBT), which was also confirmed by Western blot analysis (Fig. 5C). Similarly, the presynthesized J252, as control PROTAC compound, was able to down-regulate the HIF-1α levels in hypoxic HeLa cells. In contrast, separate incubation of JW4 or JQ1-CBT under hypoxia did not induce HIF-1α elimination (Fig. 5D), which is consistent with the static protein level of BRD4 (fig. S4). Meanwhile, inhibition of the BRD4 with JQ1 alone did not substantially alter the protein level of HIF-1α in hypoxic HeLa cells. These results unequivocally illustrate that the degradation of BRD4 interrupts the accumulation of HIF-1α in the nucleus. In addition, as compared to standard inhibition, our NTR-activated ENCTAC system may show a different perturbation pathway to manipulate the hypoxia response.

To further investigate the effects of ENCTAC degradation of BET proteins on hypoxia-regulated pathways, we evaluated expressions of hypoxia-responsive proteins. In particular, carbonic anhydrase 9 (CA9), as a cellular biomarker and pH regulator of hypoxia, is overexpressed in cells in the abnormal vasculature and tumor microenvironment (*52*). This protein is transcriptionally activated by HIF-1α. JW4 and JQ1-CBT coincubation in hypoxic HeLa cells dose-dependently reduced the amount of CA9 as a consequence of BRD4 and HIF-1α downgrade Fig. 5E). Although JQ1 treatment did not obviously suppress BRD4 and HIF-1 protein levels, it can still partially downgrade CA9 expression (fig. S6), which is possibly attributed to the inhibition of the interaction between BRD4 and CA9 promoter (*17*). In addition, similar effects were also observed in the expression of a potent angiogenic factor, vascular endothelial growth factor (VEGF; Fig. 5E and fig. S6). VEGF is induced under the hypoxia condition and functions to promote neovascularization to restore oxygen supply to the affected tissue (*53*). Quantitative analysis after ENCTAC or inhibitor treatments revealed the significant difference in the impact of BRD4 degradation on the protein levels of HIF-1α and its hypoxia-regulated targets (Fig. 5F). Specifically, almost 50% lower in HIF-1α level was observed in cells with ENCTAC treatments in contrast to the static HIF-1α level done by JQ1 incubation with the same concentration, suggesting that degradation of BRD4 by ENCTACs provided efficient down-regulation of its hypoxia-regulated proteins. The further mRNA analysis of the hypoxia-regulated genes, shown in fig. S7, indicated that HIF-1α mRNA was reduced after ENCTAC treatment in hypoxia, which is much significant than the inhibitor JQ1 and the standard PROTAC ARV-825. Similarly, our JW4 and JQ1-CBT system induced strong suppression of VEGF and c-Myc mRNA. The results of this transcription effect showed that the reduction in protein levels of the hypoxia-regulated gene may not be due to bystander effect (i.e., codegradation of interacting proteins).

ENCTAC treatments for targeted BRD4 degradation lead to notable malfunction in cell development. Once the adaptive system of microenvironment alterations is interfered by HIF-1α deductions, cells may undergo apoptosis and are thus unable to sustain the development process (*54*, *55*). To this end, we analyzed the typical cell growth factor c-Myc and observed the reduced amount after the introduction of NTR-oriented ENCTACs (Fig. 5G). In the meantime, cleavage of poly(adenosine 5′-diphosphate–ribose) polymerase (PARP) was increased indicating the elevated cell apoptosis (Fig. 5H). The apoptotic response was further detected by annexin V (AnnV)/propodium iodide (PI) staining. Confocal microscope images revealed the clear AnnV translocation and the surge in cell membrane permeability after the introduction of drugs for 12 hours (Fig. 5I). Quantification of the staining signals was recorded by flow cytometry (Fig. 5J), illustrating obvious late apoptotic cell death after BET proteolysis in hypoxia. These results indicate that microenvironment-specific degradation of BET is critical for therapeutic applications.

### In vivo ENCTAC-dependent angiogenesis

**Fig. 6.**
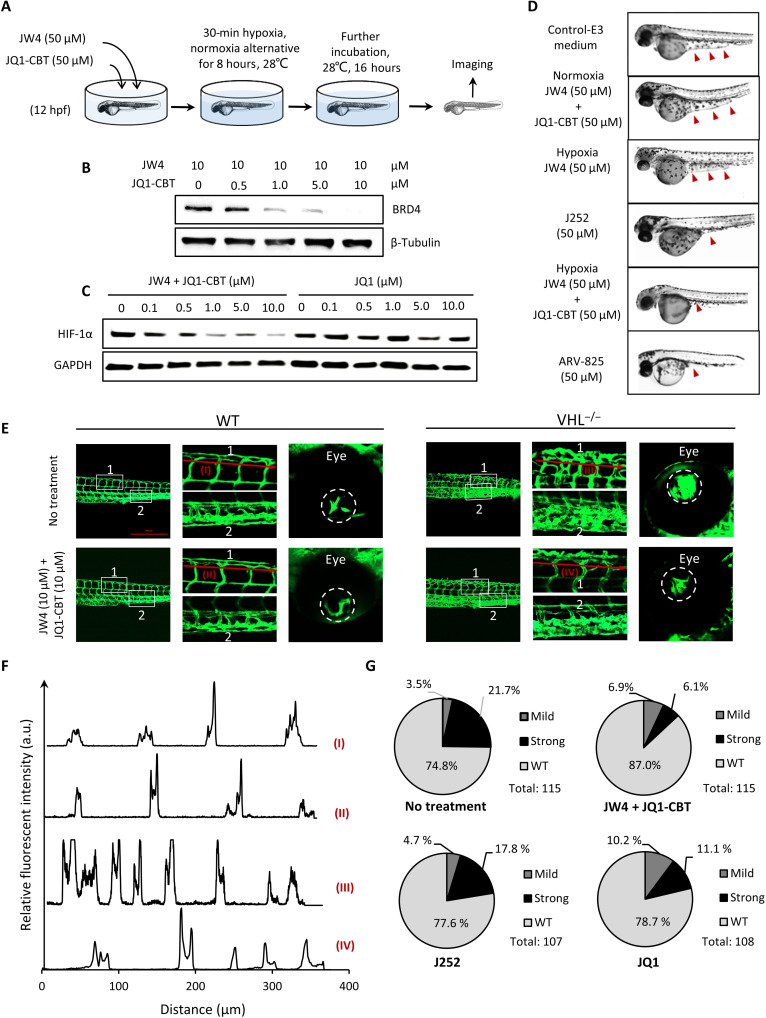
In vivo degradation of BRD4 using ENCTACs to manipulate hypoxic zebrafish development. (**A**) Scheme of zebrafish early treatment with hypoxia-activated ENCTACs. (**B**) Western blot analysis of BRD4 level in hypoxia zebrafish after treatment with different concentrations of ENCTAC molecules. (**C**) Western blot analysis of HIF-1α level in hypoxia zebrafish upon treatment with different concentrations of ENCTAC molecules or with inhibitor JQ1. (**D**) Bright-field images of zebrafish embryos under different conditions of indicated drug treatment at 36 hpf. Red arrowheads indicate yolk extension area. (**E**) Fluorescent imaging of zebrafish larvae phenotype with blood vessel trackers (*fli1:eGFP^y1^*) in wild-type (WT), and von Hippel–Lindau (*vhl*) mutant (*vhl*^−/−^) larvae with and without ENCTAC treatment. Blood vessel tracker (green), λ_ex_ = 488 nm and λ_em_ = 520/30 nm. Scale bar, 500 μm. (**F**) Fluorescent intensity spectra of blood vessel alignment along red line (I to IV) indicated in (E). (**G**) Statistical numbers of vascularization phenotypes in zebrafish larvae with and without ENCTAC treatments or with PROTAC, J252, or inhibitor JQ1 (10 μM).

We extended our analysis of hypoxia-activated ENCTACs to ascertain its effect in vivo using zebrafish larvae. The relatively high level of BRD4 and the structural conservation of CRBN and JQ1 binding sites between zebrafish and human make it an ideal model for protein-ligand activity (*56*, *57*). Zebrafish embryos were used to evaluate the in vivo activity of ENCTACs. Twelve hours postfertilization (hpf), zebrafish were incubated with hypoxia-responsive ENCTAC compounds at 50 μM under alternating hypoxia-normoxia conditions for 8 hours at 28°C. Embryos without drug treatment, maintained only in normoxia or treated under hypoxic conditions with only JW4, were used as negative controls. Embryos treated with our presynthesized compound J252 or standard PROTAC ARV-825 were used as positive controls. Whole extracts of embryos upon treatment were collected and analyzed by immunoblotting. As revealed in Fig. 6B, our ENCTAC system under hypoxic conditions, considerably reduced the level of BRD4 protein in embryos. This hypoxia-mediated BRD4 degradation also resulted in lower expression level of HIF-1α, in contrast to JQ1-mediated inhibition of BRD4 (Fig. 6C). Phenotypic observation after treatment illustrated that embryos incubated with ENCTACs under hypoxia showed thinner yolk sac extension compared to those of controls. Embryos treated with directly linked PROTAC control molecule J252 or ARV-825 showed a similarly reduced yolk extension at 36 hpf (Fig. 6D). Notably, treatment of zebrafish embryos under hypoxia using the standard ARV-825 PROTAC degrader also indicated the promising BRD4 disruption and low expression of HIF-1α (fig. S8). The results suggested the effective function of the ENCTAC system in zebrafish.

In line with the efficiency of our system in down-regulating the hypoxia-responsive proteins including VEGF in cancer cells and its ability to reduce HIF-1α level via BRD4 depletion in zebrafish, we next evaluated the ENCTAC capacity in modulating angiogenesis, one typical hallmark of tumors. We used *vhl^hu2117^* mutant embryos, which bear mutation of a negative regulator of HIF-1α , as an in vivo model system with excess HIF-1α (*58*). The excessive level of HIF-1α and the subsequent up-regulation of VEGFA in zebrafish embryos resulted in extensive vascularization (fig. S9). The mutant embryos showed NTR activation as detected by our NTR-responsive dye in comparison with wild-type larvae (fig. S10). While the ENCTAC compounds showed negligible effect on wild-type vascular patterning, it reduced the number and tortuosity of the vessels in the tail plexus and retina of the *vhl^hu2117^* homozygous mutants (Fig. 6, E and F). Incrosses of *vhl^hu2117^* heterozygous generated approximately 25% embryos with hypervascularization as the tail plexus. The ENCTAC system significantly decreased the number of hypervascularized embryos (13% of total) compared to the nontreated group. Meanwhile, treatment of the embryos from *vhl^hu2117^* heterozygous incrosses with the J252 or JQ1 alone slightly lowered the penetrance of the hypervascularized phenotype with 22.5 and 21.3% vascularized larvae remaining, respectively (Fig. 6G). This selective suppression of the severe vascular phenotype further illustrated the superiority of our ENCTAC system in vivo.

### ENCTAC activity in mice with solid tumors

**Fig. 7.**
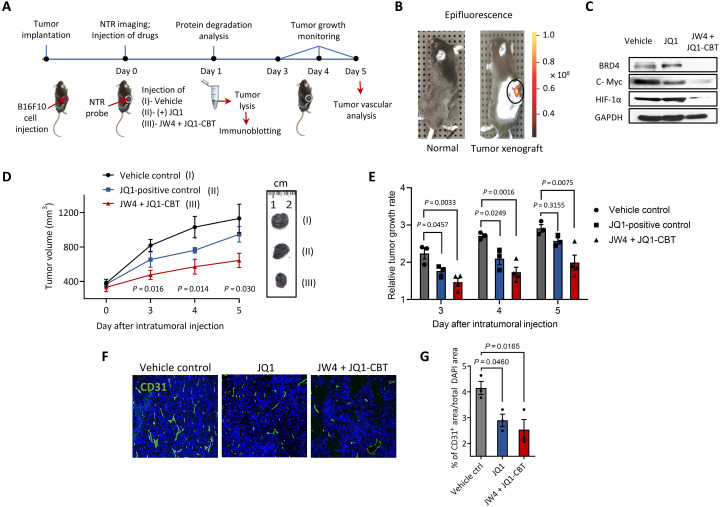
Tumor inhibition by ENCTACs assisted BRD4 degradation. (**A**) Schematic diagram shows the treatment of ENCTACs in the mouse melanoma xenograft model. (**B**) Fluorescence image of NTR activity in solid tumor. Near-infrared fluorescence reporter was intratumorally injected, and the image was taken 30 min later. (**C**) Immunoblot for BRD4, c-Myc, HIF-1α, and GAPDH in tumor lysates collected from mice treated with JW4 and JQ1-CBT (5 mg/kg) mixture, JQ1 (5 mg/kg) alone, or vehicle twice for 4 hours. (**D**) Representative pictures and quantitative analysis of tumor volume (means ± SD) of vehicle-treated mice (*n* = 3) or mice treated with JW4, JQ1-CBT mixture (5 mg/kg; *n* = 4), or JQ1-treated mice (*n* = 3) for 5 days. (**E**) Relative tumor growth rate under different treatments as indicated for 5 days. (**F**) Immunohistochemistry staining with a vascular marker CD31 (green) and nucleus marker 4′,6-diamidino-2-phenylindole (DAPI) (blue) of tumors subjected to ENCTACs, (+)-JQ1, or vehicle control. (**G**) Quantification of total vascular area in tumors subjected to ENCTACs, (+)-JQ1, or vehicle control.

We further investigated the feasibility of tumor microenvironment–activatable epigenetic BRD4 degradation and validated the potential antitumor efficacy of ENCTACs in a mouse melanoma xenograft model. Specifically, we implanted the B16F10 mouse melanoma cells into the right flank of C57BL/6 mice. Tumor-bearing mice were subjected to targeted protein disruption and therapeutic studies once the tumor reaches the palpable size (Fig. 7A). First, the NTR enzyme activity was determined in melanoma xenograft using NTR-Cy7, a probe molecule responsive to NTR enzyme (*59*) by animal imaging scanning. As shown in Fig. 7B, NTR is activated in melanoma in vivo. Inspired by the promising imaging results, we further accessed the acute pharmacodynamic degradation of BRD4. In this study, tumor-bearing mice were subjected to intratumoral injection of ENCTACs, JW4 + JQ1-CBT, at the dosage of 5 mg/kg every 4 hours for 8 hours on day 1 of the treatment. Meanwhile, vehicle and JQ1 were used as controls for tumor-dependent protein degradation and therapeutic studies. As expected, the administration of ENCTAC compounds (JW4 + JQ1-CBT) showed marked degradation of BRD4. Accompanied investigations disclosed the down-regulation of HIF-1α and c-Myc (Fig. 7C), suggesting a close relationship between epigenetic regulation and tumor hypoxia. Although injection of JQ1 alone reduced tumor growth and downgraded c-Myc expression in the xenograft model, this epigenetic BET inhibition had only a trivial effect on altering BRD4 and HIF-1α protein levels, in contrast to our ENCTAC compounds, indicating a different mechanism for suppressing the tumor response to hypoxia. The antitumor efficacy of our ENCTAC system was evaluated by daily treatment with the compounds at equimolar concentrations. Serial volumetric measurements illustrated a significant attenuation of tumor progression using ENCTACs compared with vehicle controls and JQ1 administrations (Fig. 7D), and decreased tumor growth rates were also observed (Fig. 7E). To compare the efficacy of BET inhibition to BET degradation in tumor vascularization, we selected CD31, a standard blood vessel marker. Our immunofluorescence staining of excised tumors at the end of the efficacy study on day 5 suggested a significant reduction in tumor vessel area compared with vehicle controls and JQ1 administration (Fig. 7, F and G), indicating the superior efficacy of our ENCTAC degradation on the tumor hallmark, angiogenesis. These findings demonstrate an improved tumor suppression efficacy of hypoxia-activated ENCTACs over JQ1 in the mouse xenograft model.

**Fig. 8.**
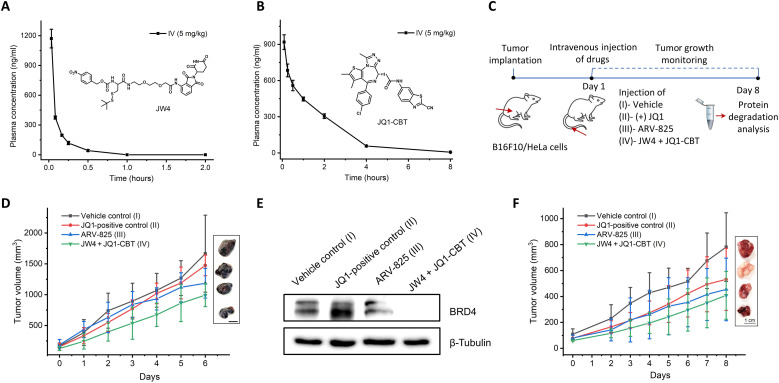
Pharmacokinetic analysis and tumor inhibition studies of ENCTACs. (**A** and **B**) Plasma concentration–time profiles of JW4 (A) and JQ1-CBT (X = NH) (B) after intravenous dosing (5 mg/kg). (**C**) Schematic diagram for the treatment of ENCTACs in the xenograft mouse model with melanoma and HeLa tumor. (**D**) Representative images and quantitative analysis of tumor volume (means ± SD) of melanoma mice treated with vehicle (*n* = 4), JQ1 (5 mg/kg, *n* = 5), ARV-825 (5 mg/kg; *n* = 5), or JW4 + JQ1-CBT mixture (5 mg/kg; *n* = 5) for 6 days. (**E**) Western blot analysis of BRD4 levels in melanoma tumor after intravenous injection of vehicle, JQ1, ARV-825, or JW4 + JQ1-CBT (5 mg/kg). (**F**) Representative images and quantitative analysis of tumor volume (means ± SD) of HeLa tumor–bearing mice treated with vehicle control (*n* = 3), JQ1 (5 mg/kg, *n* = 3), ARV- 825 (5 mg/kg; *n* = 3), or JW4 + JQ1-CBT mixture (5 mg/kg; *n* = 3) for 8 days.

Furthermore, we performed pharmacokinetic studies to evaluate the performance of the ENCTAC system by intravenous injections of JW4 and JQ1-CBT (X = NH; fig. S12) into mice at a dose of 5 mg/kg. In contrast, similar in vivo pharmacokinetic analysis was also conducted using the standard ARV-825 degrader as our reference. As shown in Fig. 8 (A and B), our ENCTAC warhead molecules exhibited favorable bioavailability with the half-life of the JW4 observed for 0.12 hours, positively comparable to the value for the control ARV-825 molecule, while the value of JQ1-CBT is observed for 1.12 hours, suggesting its good stability in vivo (fig. S11). These data suggest a great potential of our ENCTAC system for tumor-selective degradation of targeted proteins in vivo.

Encouraged by this favorable pharmacokinetic analysis, we further validated the effect of our ENCTAC warhead components on tumor growth in different mouse models bearing melanoma and HeLa tumors (Fig. 8C). In these typical studies, our ENCTAC components and standard control JQ1 and ARV-825 were administered via intravenous injections to tumor-bearing mice with the dose of 5 mg/kg for eight consecutive days. As shown in Fig. 8D, the in vivo studies provided evidence of our ENCTAC system for potent tumor inhibition on melanoma. Moreover, simultaneous introduction of our ENCTAC components also induce efficient tumor degradation of BRD4 with better effect as compared to the reference PROTAC compound of ARV-825 or the inhibitor JQ1 (Fig. 8E). Consistently, the prolonged tumor-suppressive effects were also identified in the HeLa tumor xenograft model following the treatment of our ENCTAC components, which was more significant than those of ARV-825 and JQ1 control molecules (Fig. 8F). Moreover, along the whole process, there was minimum body weight loss in mice treated with our components (fig. S11). These results reveal the great potential of our ENCTAC system for targeted tumor protein disruption and therapeutic efficacy against tumors in living system.

## DISCUSSION

Taking advantage of the unique capability of specific enzyme reactions, here, we have presented an ENCTAC strategy capable of responding specifically to environmental stimuli to induce activation-feedback degradation of an epigenetic protein, BRD4. Environmentally activatable ENCTAC formation and BRD4 protein degradation were confirmed upon incubation of NTR-pomalidomide and JQ1-CBT substrates in hypoxic cell and animal models of angiogenesis with tumor. Specifically, immunoblot analysis after stimulation by hypoxia revealed a notable decrease in BRD4 level in multiple tumor cell lines with increasing concentration of the small-molecule substrates. Along with the disruption of BRD4 expression, an obvious proliferation arrest was observed in the tumor cells, suggesting the feasibility of ENCTACs for inhibiting cancer growth. Similarly, ENCTAC-associated BRD4 elimination was also clearly executed in zebrafish models of hypoxic stress and mouse solid tumor model. Although direct cross-linking of pomalidomide and JQ1 substrate, as a control similar to that of traditional PROTAC design, could indeed disrupt epigenetic BRD4 in live cells, there was no selectivity to differentiate between normoxia and hypoxia cells. This could indicate a detrimental off-target effect. Moreover, the large molecular weight of the conventional PROTAC molecule also raised the concern about cell permeability and efficient protein perturbation. Although the potential effect of the ENCTACs on the stability of other proteins in the cells could not be completely excluded, our fragmented ENCTAC strategy and in situ formation of ENCTACs upon site-selective activation exhibit promising prospect for the targeted protein degradation in living system.

As a pernicious effect on cells and animals, hypoxia is usually a characteristic feature of the tumor microenvironment in most solid tumors, largely due to the inability of tortuous leaky tumor vessels to meet the metabolic demand of rapid proliferation of tumor cells (*60*). This critical pathway of oxygen deprivation, mediated by HIFs, can up-regulate hypoxia-induced gene expression associated with hostile homeostasis in tumors to adapt their formation, metastasis, malignant proliferation, and drug resistance (*61*). These tumor modulatory factors serve as attractive targets for the development of previously unidentified anticancer therapeutics (*62*–*64*). Accumulating evidence have demonstrated that increased levels of HIF and their dependence on epigenetic regulation are closely related to hypoxia. Unfortunately, the detailed mechanism remains unclear (*65*–*67*). In this study, we realized that the selective epigenetic BRD4 protein degradation in hypoxic cells could down-regulate CA9 expression, a key hypoxia pH gene regulator overexpressed in various tumors to adapt a hypoxia environment. Meanwhile, a marked reduction in VEGF levels, a master regulator of angiogenesis, was also identified in the course of selective BRD4 disruption in hypoxic cells and zebrafish, clearly indicating a promising effect on antiangiogenesis. This combined perturbation of CA9 and VEGF triggered by specific ENCTAC degradation may provide the possibility to enhance sensitivity toward tumor therapies. Although our in vitro and in vivo investigation demonstrated that the epigenetic BRD4 substrate JQ1 alone can indeed function as a BET inhibitor and impair hypoxia-induced the CA9 and VEGF expression, this occupancy-driven BRD4 inhibition occurred nonspecifically under both hypoxia and normoxia conditions and that epigenetic protein inhibition did not markedly alter cellular HIF-1α expression. Conversely, along with our selective ENCTAC-mediated BRD4 degradation, a substantial decrease in HIF-1α expression was observed in hypoxic cells and animals, suggesting that epigenetic regulators play key roles in mediating HIF-1α transcription in hypoxia. This environment-responsive HIF perturbation indicated an apparent antiangiogenic effect that could considerably reduce vascularization in hypoxic zebrafish models and the mouse model of solid tumors. Furthermore, our enzyme-derived ENCTACs exhibited promising pharmacokinetic profile and potent activity against tumor growth in vivo, suggesting its possibility of developing potential therapeutic targets.

In summary, we have presented a first-in-class ENCTACs that can specifically respond to NTR stimulation and selectively degrade BRD4 protein in vitro and in vivo under hypoxia. Environmentally activatable ENCTACs could impair the cellular response to hypoxic stress by directly down-regulating HIF-1α levels and other multiple downstream targets including CA9 and VEGF with the mechanism different from those of physical molecular inhibition, thus opening a whole innovative avenue for the therapeutic development. This well-defined ENCTAC design can realize the controlled cross-linking of two small-molecule warheads and targeted elimination of proteins in a specific environment without prior covalent conjugation as observed in traditional PROTAC, minimizing poor pharmacological performance due to large molecular weight. Together with its potential therapeutic impacts on tumor suppression, we anticipate that these ENCTAC strategies may provide valuable insights on enhanced flexible practicality and druggable potency for disease-related protein degradation in the future.

## MATERIALS AND METHODS

### Compounds

Synthesis of new compounds and their intermediates is described in the Supplementary Materials. (+)-JQ1 was purchased from MedChemExpress, pomalidomide-PEG_2_-NH_2_, pomalidomide-C3-NH_2_ was purchased from Sigma-Aldrich.

### NTR enzymatic cleavage in vitro

Procedure to examine the time required for NTR to perform enzymatic cleavage. To an MS vial was sequentially mixed with JW4 (10 μM), NADH (0.5 mM), and NTR (40 μg/ml) in phosphate-buffered saline (PBS; 10 mM) (pH 7.4) into a total volume of 500 μl. The mixture in the vial was vortexed and incubated at 37°C with varying time control of 0 to 240 min. Structural mass analysis was performed using a Thermo Finnigan LCQ Deca XP MAX Mass instrument to observe any change in molecular mass. The experimental procedures were repeated for J268 for the analysis of enzymatic cleavage.

#### Procedure for the analysis of the optimal NTR concentration

An MS vial was sequentially mixed with JW4 (10 μM), NADH (0.5 mM), and NTR with a concentration range of 0 to 40 μg/ml in PBS (10 mM; pH 7.4) in a final volume of 500 μl. The mixture in the vial was vortexed and incubated at 37°C for 120 min before being subjected to structural mass analysis. The experimental procedures were repeated for analysis of J268 enzymatic cleavage.

### MS analysis for the click reaction

Two-millimeter MS vial was sequentially added J266 (10 μM), JQ1-CBT (10 μM), and TCEP (200 μM) in a solution of pure deionized H_2_O to give a total volume of 500 μl. The compounds in the vial were vortexed for a few seconds to homogenize the mixture and then heated at 37° to 40°C for 30, 60, 90, and 120 min to monitor the click reaction between cleaved J266 and JQ1-CBT over time. Structural mass analysis was performed using the Thermo Finnigan LCQ Deca XP MAX mass instrument. A correlative line plot was generated to observe the kinetics and spontaneity of the click reaction.

### System construction for simulation

All J252, ARV-825, J242, and T208 systems are based on the two protein-ligand complexes; one is JQ1-(S) binding with protein BRD4 BD1 [Protein Data Bank (PDB) ID 3MXF], and the other one is pomalidomide binding with protein CRBN (PDB ID 4CI3). Both the proteins and the ligands were constructed using the CHARMM-GUI tool. Parameters of proteins were based on the CHARMM36 force field, and parameters of the ligands were based on the CHARMM General Force Field. All the topological files were converted to GROMACS format using CHARMM-GUI force field converter. The system was solvated with transferable intermolecular potential 3P water molecules, and counterions were added to neutralize the system. The constructed protein/ligand systems were subjected molecular dynamics simulation using GROMACS 5.1.2 software. The LINCS algorithm was used to constrain bonds between heavy atoms and hydrogen to enable a time step of 2 fs. A 1.2-nm cutoff was used for van der Waals interaction and short-range electrostatic interaction calculations, and particle mesh Ewald method was implemented for long-range electrostatic calculations. Simulation temperature was maintained at 300 K using a V-rescale thermostat and 1-bar pressure using Parrinello-Rahman barostat. All simulations were run for 1 ns to equilibrate the systems.

### Cell lines and proliferation condition

Adherent cells were cultured in TPP® tissue culture flasks (25 cm^2^) at 37°C and 5% CO_2_. HeLa cells [American Type Culture Collection (ATCC)], HEK-293T cells (ATCC), human breast cancer MDA-MB-231 (ATCC), mouse breast cancer 4T1 cells (ATCC), and mouse melanoma cell line B16F1 (Manassas, VA, USA) were cultured in Dulbecco’s modified Eagle’s medium (DMEM) supplemented with 10% fetal bovine serum (FBS) and 1% penicillin/streptomycin at 37°C and 5% CO_2_ in a humidified atmosphere. All cells were routinely split one to two times per week when they were 90% confluent and were not used beyond passage 30.

### Protein degradation analysis by Western blot

HeLa cells and HEK-293T (1.5 × 10^6^ cells/ml) were treated with J266 (10 μM) for 6 hours before incubation with JQ1-CBT at the indicated concentration and time. This entire incubation was performed under normoxia conditions. For the NTR moiety–caged JW4, cells were treated with JW4 (10 μM) 6 hours before the addition of JQ1-CBT for subsequent 12 hours, entirely performed under hypoxic condition. Comparison of downstream effect between controls and drug treated cells were completely executed under hypoxic conditions. For processing, the cells were washed twice with cold PBS (pH 7.4) and lysed with radioimmunoprecipitation assay buffer supplemented with a protease inhibitor cocktail (Roche) and a phosphatase inhibitor cocktail (Cell Signaling Technologies) for 10 min at 4°C. Lysed cells were then collected and centrifuged at 13,500 rpm for 20 min at 4°C. The supernatant was collected, and the protein concentration was analyzed using the NanoDrop 2000/2000c Spectrophotometers. Lysate volumes were fine-tuned and were loaded onto a Mini-PROTEAN TGX 4 to 20% Precast Gel (Bio-Rad) and separated by SDS–polyacrylamide gel electrophoresis. Then, the gel was transferred onto a polyvinylidene difluoride membrane (100 V for 90 min at 4°C), washed twice with tris-buffered saline containing Tween 20 (TBS-T), and blocked with 1% bovine serum albumin (BSA) for 1 hour at room temperature. Next, the membrane was incubated overnight at 4°C with primary antibody, washed three times with TBS-T, and then incubated with goat anti-rabbit immunoglobulin G (IgG) (H + L) secondary antibody, horseradish peroxidase (#31460, Thermo Fisher Scientific; 1:20,000 dilution factor) for 1 hour. The membrane was supplemented with the Radiance Q chemiluminescent ECL substrate (Azure Biosystems) and was visualized using Amersham ImageQuant 800 biomolecular imager. Primary antibodies against BRD4 (#13440; 1:1000 dilution factor), HIF-1α (#36169; 1:1000 dilution factor), and glyceraldehyde-3-phosphate dehydrogenase (GAPDH; #5174; 1:1000 dilution factor) were purchased from Cell Signaling Technologies; c-Myc (#700648; 1:1000 dilution factor), PARP1 (#MA5-15031; 1:500 dilution factor), VEGF (#P802; 1:500 dilution factor), and CA-IX (#MA5-29076; 1:1000 dilution factor) were purchased from Thermo Fisher Scientific; BRD4 (ab243862; 1:1000 dilution factor) and β-tubulin (ab6046; 1:10,000 dilution factor) were purchased from Abcam.

### Immunofluorescence imaging of HIF-1α

HeLa cells were plated at a density of 8 × 10^4^ cells/ml in each well of eight-well ibidi dishes before the experiment. To sustain hypoxia-induced factor throughout drug treatment, drug incubation was performed entirely in hypoxic chamber. Initially, HeLa cells were treated with JW4 (10 μM) for 6 hours. After that, JQ1-CBT (10, 5, and 1 μM) was added to the corresponding wells and incubated for another 12 hours. Control cells were left untreated under normoxia condition in a separate ibidi dishes. Cells were fixed with 4% paraformaldehyde for 15 min, permeabilized with 0.25% TBS-T, and blocked with 1% BSA for 20 min at room temperature after drug treatment. All the different cell groups were then incubated with rabbit HIF-1α polyclonal antibody (PA1-16601, Thermo Fisher Scientific; 1:100 dilution factor) for 2 hours at room temperature. Then, the primary antibody–labeled cells were rinsed with PBS (2×) and were stained next with Alexa Fluor 488–goat anti-rabbit IgG secondary antibody (ab150077, Abcam; 1:1000 dilution factor) for 1 hour at room temperature. The cytoskeleton networks of cells were stained with Alexa Fluor 594 anti–α-tubulin antibody [DM1A]–microtubule marker (ab195889, Abcam; 1:200 dilution factor), and the nuclei were stained with Hoechst 33258 (1 μM). The fluorescence of the alleviated HIF-1α expression upon drug treatment was visualized using a Carl Zeiss LSM 800 microscope (Hoechst 33258, λ_ex_ = 405 nm and λ_em_ = 460/50 nm; Alexa Fluor 488, λ_ex_ = 488 nm and λ_em_ = 515/30 nm; Alexa Fluor 594, λ_ex_ = 561 nm and λ_em_ = 617/20 nm).

### Quantitative real-time polymerase chain reaction

Total RNA was extracted and purified with RNAzol RT (catalog no. 888-841-0900, Molecular Research Center) before being reverse-transcribed to cDNA with qScript cDNA SuperMix (157031, Quanta Biosciences). Polymerase chain reaction (PCR) was conducted with PrecisionFAST qPCR Master Mix (FASR-LR-SY, Primerdesign Ltd., UK) with use of Applied Biosystems StepOnePlus Real-Time PCR System (Life Technologies). The expression levels of respective target genes were normalized to GAPDH, and relative gene expressions were calculated using standard 2^−ΔΔCT^ method. Primers used in this study are listed in table S1.

### Cell viability assay

HeLa cells were seeded at density of 1 × 10^4^ cells/ml with a total volume of 100 μl of DMEM in 96-well plate overnight. Subsequently, a pre-set JW4 concentration of 10 μM was incubated under hypoxic condition for 6 hours before JQ1-CBT was added at varying concentrations for 24 hours under hypoxia conditions before being replaced with TOX-8 medium solution (0.3 mg/ml) and incubated for another 4 hours. Cell viability analysis was then analyzed using a Tecan Infinite M200 microplate reader with excitation at 560 nm and emission at 590 nm. The percentile of cell viability was determined by the ratio of the fluorescence intensity of the drug-treated cells relative to untreated controls.

### Apoptosis imaging and flow cytometry analysis

Dual-staining molecular probe with AnnV and PI (#88-8005-74, Thermo Fisher Scientific) to invigilate the apoptotic condition after drug treatment. In general, drug-treated cells were incubated with 5 μl of AnnV in 100 μl of 1× binding buffer for 15 min at room temperature in the dark. The staining medium was removed and without further washing, and 1 μl of PI staining solution in 100 μl of 1× binding buffer was added to the HeLa cells for another 15 min at room temperature. Visualization was performed using a Carl Zeiss LSM 800 confocal laser microscope [fluorescein isothiocyanate (FITC)–AnnV: Ex = 488 nm, Em = 520/21 nm; PI: Ex = 561, Em = 590/30 nm] for apoptosis imaging, or using a BD LSRFortessa X-20 flow cytometer for quantification of AnnV-PI staining in the different cell groups, control, and posttreatment.

### Zebrafish experiments

Wild-type zebrafish embryos at 12 hpf were incubated with the mixture of JW4 and JQ1-CBT or with J252, JW4, standard PROTAC ARV-825 (50 μM) under alternative hypoxia-normoxia conditions for 8 hours at 28°C. After that, embryos were incubated under normoxia conditions at 28°C for 16 hours before visualization of yolk extension phenotypes under a Zeiss LSM 800 confocal microscope using the bright-field channel. Embryos with JW4 + JQ1-CBT treatment under normoxia were used as controls.

For protein degradation analysis of zebrafish, JW4 and JQ1-CBT mixture or JQ1 in different concentrations was incubated with the zebrafish embryos at 24 hpf for 8 hours with 30-min alternating intervals of hypoxia-normoxia condition. Embryos without drug treatment were added as a control. Subsequently, embryos lysis was collected, and the BRD4 and HIF-1α protein levels were analyzed by immunoblotting as described above. Primary antibodies against HIF-1α (#NB100-134; 1:1000 dilution factor) were purchased from Novus.

Vasculature analysis of zebrafish was performed using embryos derived from natural crosses of the AB wild-type strain or *vhl^+/hu2117^* heterozygous mutants on the *fli1a:egfp^y1^* transgenic background (which fluorescently marks the vasculature). JW4 and JQ1-CBT were dissolved in dimethyl sulfoxide (DMSO) and were applied to embryos at an equimolar concentration of 10 μM, with J252 (10 μM) and JQ1 (10 μM) used for control experiments. The vascular plexus was imaged on a Zeiss LSM 800 confocal microscope. All experiments were conducted in the Nanyang Technological University (NTU) Zebrafish Facility under the Institutional Animal Care and Use Committee (IACUC) #A18002.

### Pharmacokinetic studies in mice

Male C57BL/6J mice (Beijing HFK Bioscience Co. Ltd.) were treated with a single dose of JW4, ARV-825, and JQ1-CBT at 5 mg/kg for intravenous tail vein injection studies. Mice were provided free access to food and water throughout the study. The compound was formulated for intravenous injection in 2% DMSO, 20% polyethylene glycol, molecular weight 400 (PEG-400), 4% Tween 80, and 74% saline. Blood was taken from animals by retro-orbital puncture under anesthesia with isoflurane. Blood collection were done at different time points: before administration (0 hours), 0.033, 0.083, 0.167, 0.25, 0.5, 1, and 2 hours for JW4 and ARV-825; 0.083, 0.25, 0.5, 1, 2, 4, 8, and 24 hours for JQ1-CBT after administration (*n* = 4). About 0.20 ml of blood was collected in EDTA-2K anticoagulant tube, placed on ice box, and centrifuged (5000 rpm at 4°C for 5 min) within 1 hour after collection. At least 150 μl of plasma was collected in a brown tube, stored in drikold temporarily, and then transferred to −80°C refrigerator (≤−80°C) for stocking. For the biodistribution experiment, the main organs of mice—heart, liver, spleen, lung, and kidney—were collected at different time points: 0.033, 0.5, and 2 hours for JW4, ARV-825; 2, 4, 8, and 24 hours for JQ1-CBT. Organ samples were homogenized in saline (1 g of sample to 3 ml of saline) for further analysis.

A 50-μl aliquot of plasma or homogenate was added with 150 μl of internal standard (tolbutamide, 200 ng/ml) in acetonitrile. The mixture was then vortexed for 3 min under 4°C and was centrifuged for 20 min at 14,000 rpm. The supernatant (120 μl) was transferred to a new plate. The solution (1 μl) was analyzed by LC–tandem MS (LC-MS8030, Shimadzu).

For pharmacokinetic samples, the concentration of the compound in the calibration standard curve and the quality control sample were determined by linear regression, which is based on the peak area ratio of the compound to the corresponding internal standard. The weighting factor is 1/*X*^2^. Lab Solution software was used for data collection and processing from the mass spectrometer. The calculation and processing of pharmacokinetic parameters were performed using WinNonlin 6.1, which is calculated in a noncompartmental model. On the basis of the analysis of blood drug concentration-time data of all pharmacokinetic animals, the following pharmacokinetic parameters were evaluated: *C*_max_, *T*_max_, and AUC_0-t_ (area under the curve). When using WinNonlin 6.1 to calculate the pharmacokinetic parameters, the AUC_0-t_ is calculated by the trapezoidal area method, and *T*_max_ and *C*_max_ are measured values.

### Protein degradation study in tumor mouse model

C57BL/6J mice and NCr nude mice were purchased from In Vivos Pte Ltd., Singapore. Animal care and procedures were performed under the guidelines of the IACUC (SingHealth IACUC, protocol number 1595). All mice were housed in an environmentally controlled room (22°C, 40 to 60% humidity, and a 12-hour light cycle).

The mouse melanoma cell line B16F10 was purchased from the ATCC (Manassas, VA, USA). Cells were cultured in DMEM (Gibco, USA) supplemented with 10% FBS (Gibco, USA), 2 mM l-glutamine (Gibco, USA), penicillin (100 U/ml), and streptomycin (100 μg/ml; Nacalai Tesque, Kyoto, Japan). The cell line was maintained at 37°C in a humidified atmosphere of 95% air and 5% CO_2_.

B16F10 cells at a density of 2 × 10^6^ cells were subcutaneously injected into the right flank of 6- to 8-week-old mice. Once tumors became palpable, intratumoral injection of buffer solution as vehicle, JQ1, JW4, and JQ1-CBT was performed at dosage of 5 mg/kg every 4 hours up to 8 hours. For intravenous injection, JW4 + JQ1-CBT, ARV-825, JQ1, or vehicle control (2% DMSO + 20% PEG-400 + 4% Tween 80 + 74% saline) was given at the dose of 5 mg/kg by intravenous injection every day for 7 days. Tumor sizes and animal weights were measured every day. Tumor volume (in cubic millimeters) = (length * width^2^)/2. Organs were collected after all the treatments, and the level of protein was analyzed by Western blot as described above. Histology and immunofluorescence staining were processed. Specifically, the resected tumors were fixed in 4% paraformaldehyde for 48 hours, washed with PBS, and gradually transferred to 15% sucrose, followed by 30% sucrose before embedding in OCT compound (Thermo Fisher Scientific, Waltham, MA, USA). Five-micrometer cryosections of tumor samples were dehydrated and blocked for 1 hour with a blocking buffer containing 2% BSA, 1% Tween 20, 3% Triton X-100, and horse serum before incubation with primary antibodies, followed by subsequent washing incubation with secondary antibodies. The primary antibodies used were Ki67 (catalog no. ab15580, Abcam, Cambridge, UK) and CD31 (catalog no. 553370, BD Biosciences, Franklin Lakes, NJ, USA). The secondary antibodies used were goat anti-rabbit IgG (H + L) cross-adsorbed secondary antibody, Alexa Fluor 594 (catalog no. A11012, Thermo Fisher Scientific, Waltham, MA, USA), and goat anti-rat IgG (H + L) cross-adsorbed secondary antibody, Alexa Fluor 488 (catalog no. A11006, Thermo Fisher Scientific, Waltham, MA, USA). The cell nuclei were counterstained with 4′,6-diamidino-2-phenylindole (DAPI). The slides were subsequently washed with PBS, mounted with Mowiol, and visualized under a Leica TCS SP8 confocal microscope and a stimulated emission depletion microscopy 3× inverted confocal microscope. Images were captured using the EVOS M5000 imaging system (Thermo Fisher Scientific, Waltham, MA, USA) and quantified using ImageJ.

HeLa xenografts tumors were established by injecting HeLa cells (1 × 10^6^) in 50% Matrigel into the 6- to 8-week-old female NCr nude mice. For in vivo efficacy experiments, when tumors reached 60 to 200 mm^3^, mice were randomized into groups. JW4 + JQ1-CBT, ARV-825, JQ1, or vehicle control (2% DMSO + 20% PEG_400_ + 4% Tween 80 + 74% saline) was given at the dose of 5 mg/kg by intravenous injection every day for 7 days. Tumor sizes and animal weights were measured every day. Tumor volume (in cubic millimeters) = (length * width^2^)/2.

## Supplementary Materials

### This PDF file includes:

Figs. S1 to S11 Table S1 Chemical synthetic procedures Instrumentation for chemical structural elucidation Source data 1 and 2

## Notes

View/request a protocol for this paper from *Bio-protocol*.
