# Supplementary material for: Hypoxia deactivates epigenetic feedbacks via enzyme-derived clicking proteolysis-targeting chimeras
Source: Sci Adv. 2022 Dec 14;8(50):eabq2216. doi: 10.1126/sciadv.abq2216 (PMC9750146; doi:10.1126/sciadv.abq2216)
Supplement: Supplementary file 1 — Figs. S1 to S11 Table S1 Chemical synthetic procedures Instrumentation for chemical structural elucidation Source data 1 and 2 [file sciadv.abq2216_sm.pdf]

Science Advances
AAAS

Supplementary Materials for

Hypoxia deactivates epigenetic feedbacks via enzyme-derived clicking proteolysis-targeting chimeras

Thang Cong Do *et al.*

Corresponding author: Bengang Xing, bengang@ntu.edu.sg; Xiaomeng Wang, xiaomeng.wang@duke-nus.edu.sg

Sci. Adv. **8**, eabq2216 (2022)
DOI: 10.1126/sciadv.abq2216

This PDF file includes:

Figs. S1 to S11
Table S1
Chemical synthetic procedures
Instrumentation for chemical structural elucidation
Source data 1 and 2

Supplementary Text

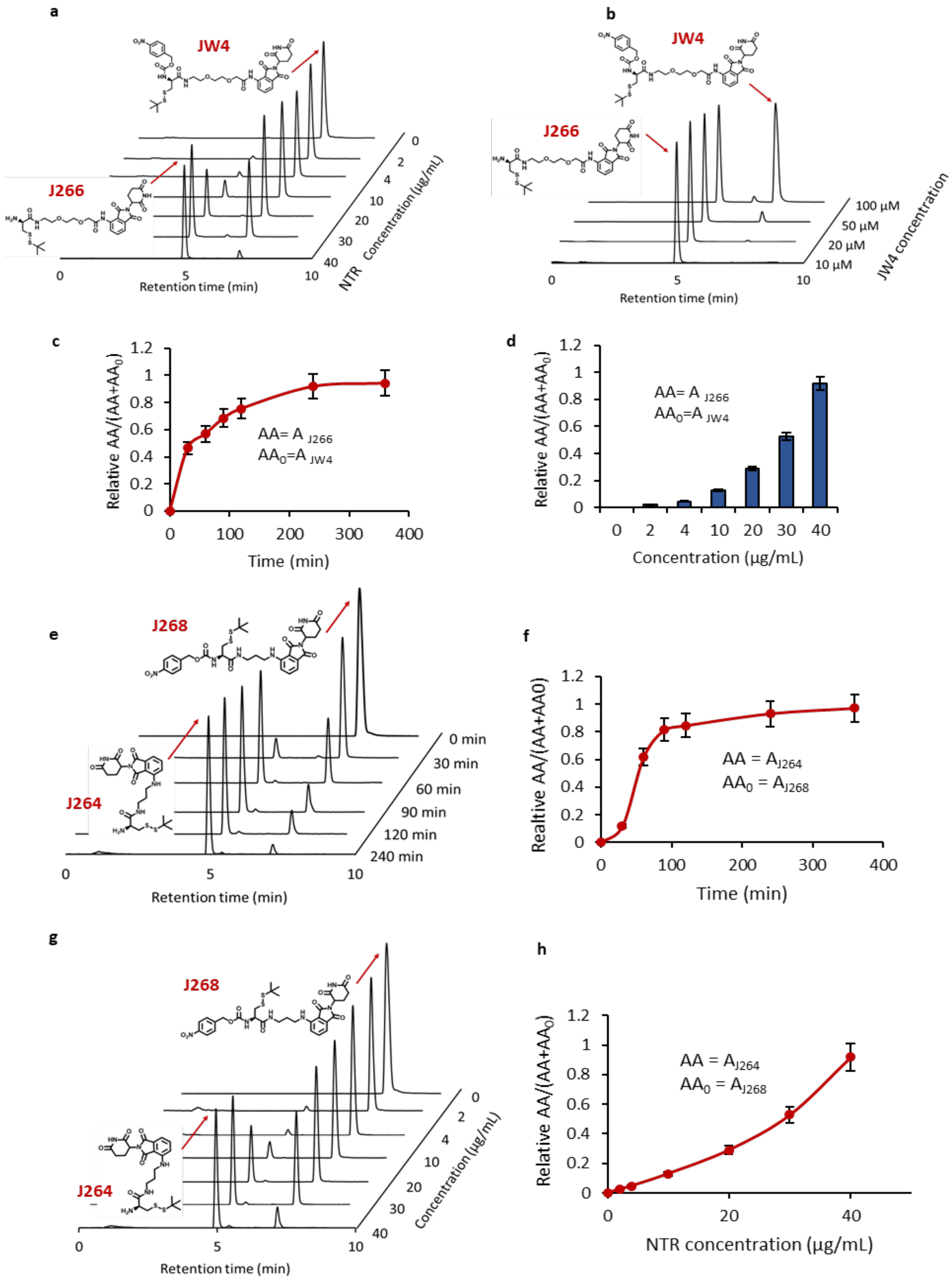

Fig. S1. **Dynamic NTR cleavage of ENCTACs components**. **a**, LC-MS spectra of NTR uncaging JW4 (10 μM) to form J266 at different concentration of NTR enzyme in PBS (pH 7.4, 10 mM). **b**, LC-MS spectra of NTR uncaging of JW4 substrate at various concentrations to form J266 in NTR enzyme (40 μg/ml) in 3 h. **c**, Ratio-metric peak areas of J266 relative to JW4 over multiple time points. **d**, Ratio-metric peak areas of J266 relative to JW4 over multiple concentration of NTR enzyme for 2 h. **e**, LC-MS spectra of time-dependent NTR uncaging of J268 (10 μM) to form J264 in NTR enzyme in PBS (pH 7.4, 10 mM). **f**, Ratio-metric peak areas of J264 relative to J268 over multiple time points in **e**. **g**, LC-MS spectra of NTR concentration-dependent uncaging of J268 (10 μM) to form J264 in PBS (pH 7.4, 10 mM). **h**, Ratio-metric peak areas of J264 relative to J268 over multiple concentrations of the NTR enzyme during 2 h.

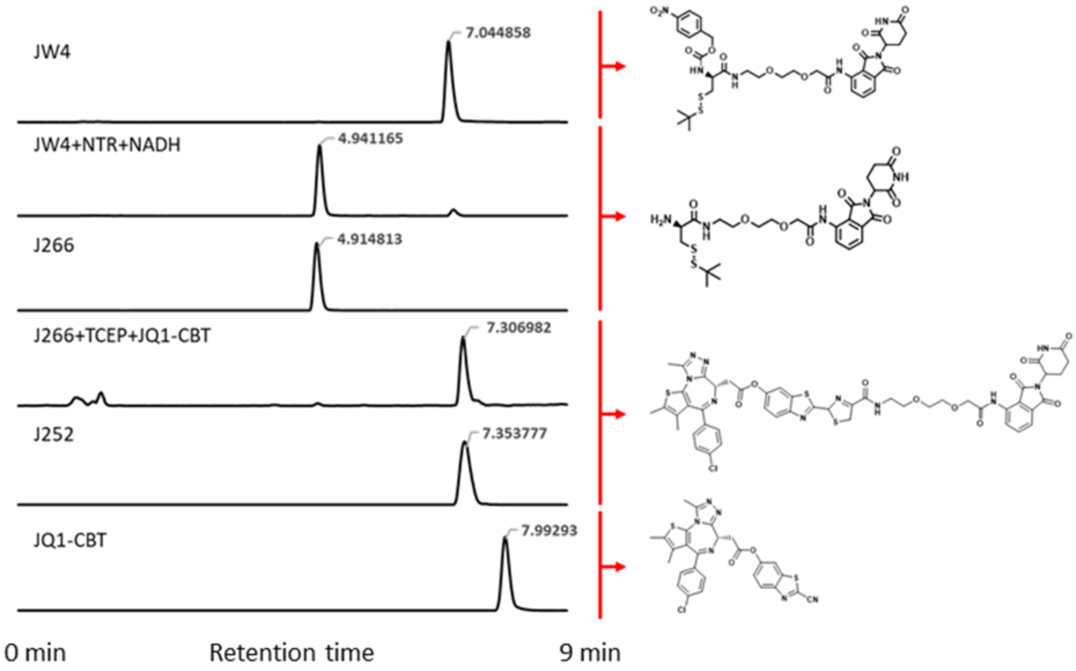

Fig. S2. One-pot continuous cleavage and formation of click-J252. LC-MS spectra of continuous one-pot NTR uncaging of JW4 (10 μM) in NTR enzyme (40 μg/ml) and subsequent cleavage of J266 by TCEP to form the click product-J252 in the presence of JQ1-CBT (10 μM) (reference spectra of pre-synthesized J266 and J252 were added).

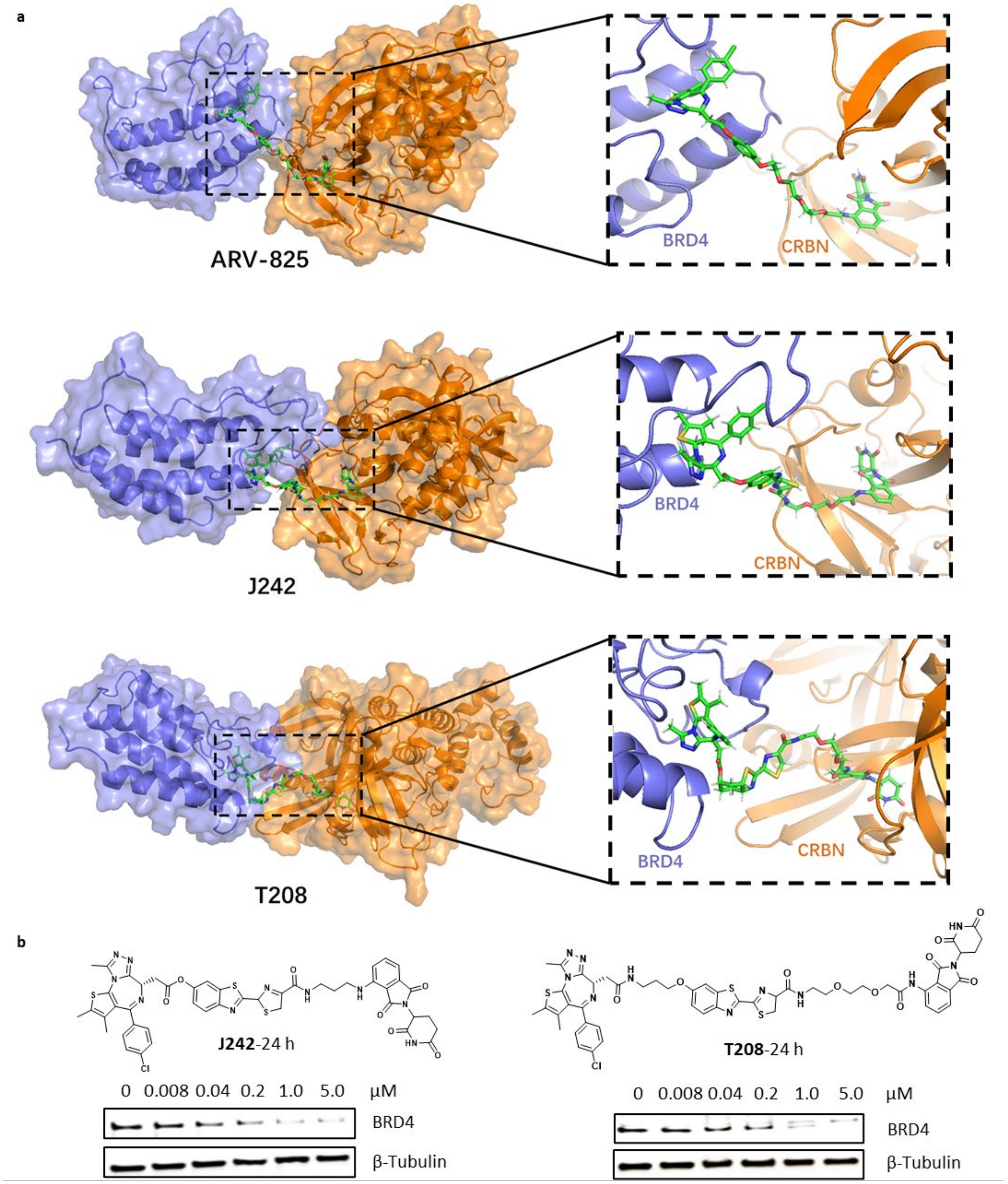

Fig. S3. **Molecular dynamics simulation and BRD4 degradation after treatment with J242 and T208.** a, ARV-825, J242 and T208 interacting with CRBN (gold) and BRD4 (blue) proteins. (snapshot at 1 ns) b, Western blot analysis of BRD4 protein after treatment with J242 and T208 for 24 h at the indicated concentration and β-tubulin as an internal control.

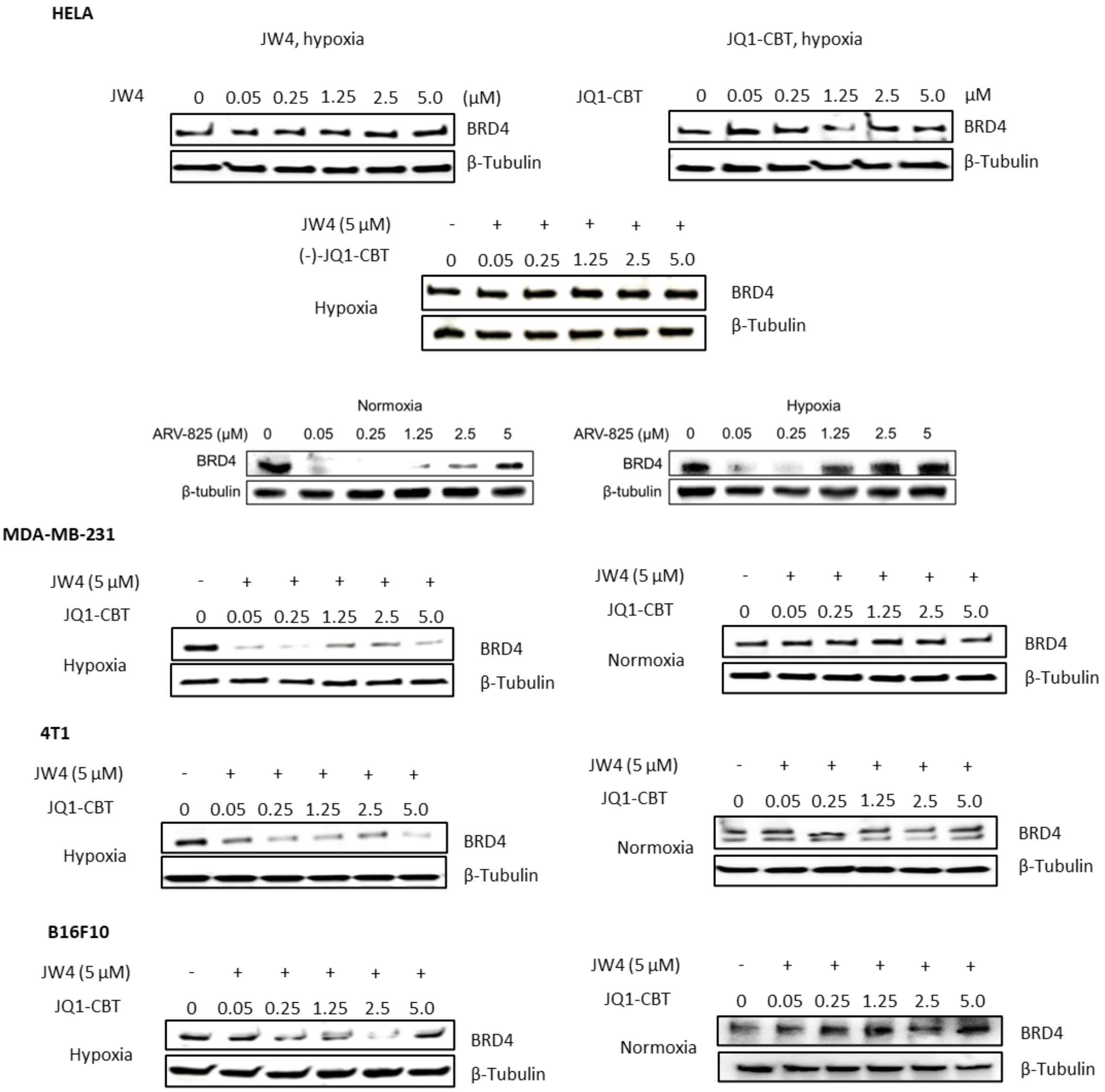

Fig. S4. Immunoblot for analysis of BRD4 levels under different control conditions. Immunoblot for BRD4 levels of HeLa cells after 12 h treatment with separated ENCTACs fragments (JW4 or JQ1-CBT) or combined JW4 and inactive stereoisomer (-)-JQ1-CBT. ARV-825 was used as positive controls in treatment of HeLa cells under hypoxia and normoxia condition with indicated concentration for 12 h. Immunoblot for BRD4 levels of MDA-MB-231, 4T1, B16F10 cells treated with a combination of JW4 and JQ1-CBT under hypoxia or normoxia conditions. β-tubulin was used as an internal control.

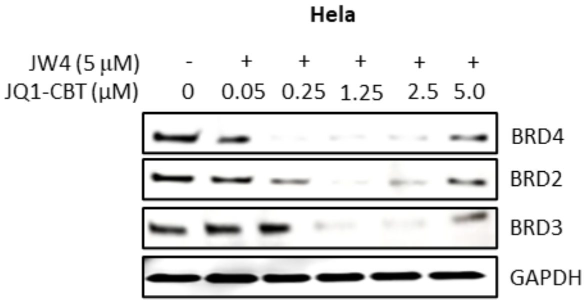

Fig. S5. Western blot analysis of BRD4, BRD2 and BRD3 levels after ENCTAC treatment with indicated concentration in hypoxic-treated HeLa cells.

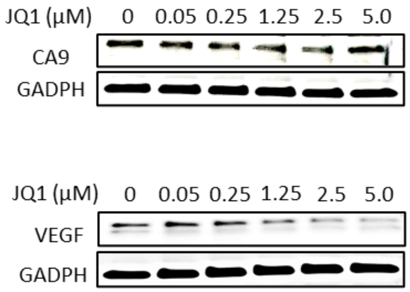

Fig. S6. **Western blot analysis of VEGF and CA9 levels after (+)-JQ1 treatment** with indicated concentration in hypoxic-treated HeLa cells.

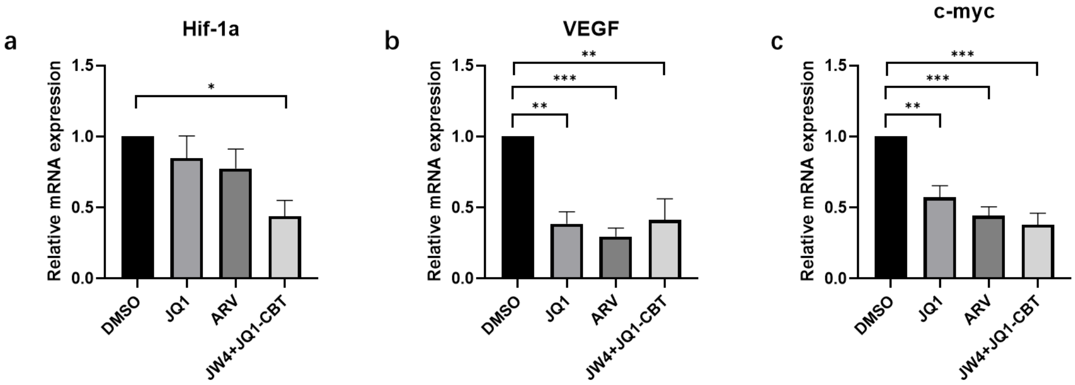

Fig. S7. mRNA expression levels of HIF-1α, VEGF and c-Myc after JQ1, ARV-825 or ENCTAC treatments. Disparate levels of mRNA expression was analyzed by one-way ANOVA followed by post-hoc turkey test.

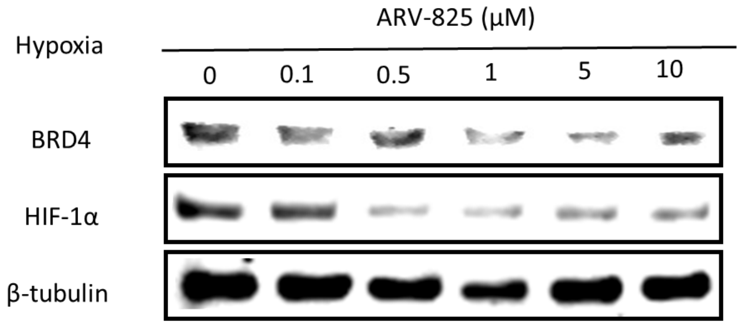

Fig. S8. Analysis of ARV-825 effect on BRD4 degradation in zebrafish. Western blot analysis of BRD4 and HIF-1α protein levels in zebrafish after hypoxia treatment with different concentrations of ARV-825 (24 h).

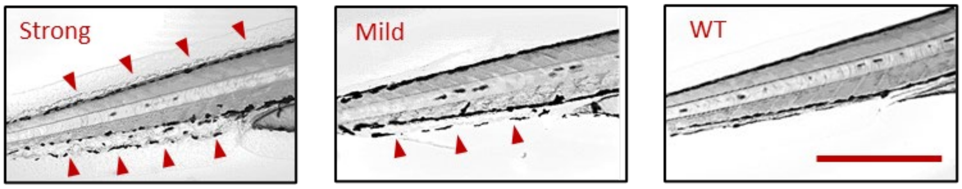

Fig. S9. Brightfield imaging of VHL mutant transgenic zebrafish or wild type zebrafish with different level of vascularization due to angiogenesis. The strong, mild, wild type (WT) was classified based on the order of blood vessels as indicated in arrows coloured in red. Scale bar: 500 µm.

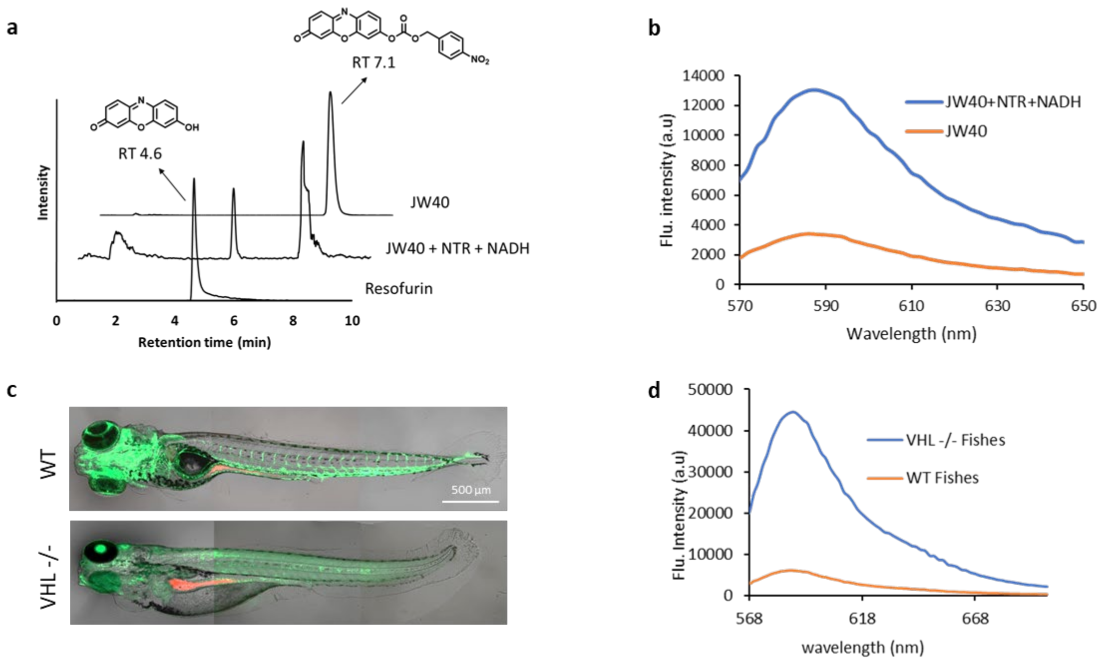

Fig. S10. **JW40 fluorescent dye for NTR detection in hypoxic zebrafish larvae**. **a**, LC-MS spectra of NTR uncaging of JW40 (10 µM) in NTR enzyme (40 µg/ml) to produce resorufin dye. **b**, Fluorescence spectra of JW40 (10 µM) in NTR enzyme (40 µg/ml) in PBS (pH 7.4, 10 mM) during 8 h incubation at 37 °C and JW40 (10 µM) without enzyme treatment. **c**, Confocal imaging of $VHL^{-/-}$ transgenic zebrafish or wide type zebrafish after treatment with JW40 (50 µM) for 12 h. VHL mutant larvae showed significant enhancement in red fluorescence from NTR uncaging of JW40. Green: blood vessel tracker, λ_{ex}: 488 nm, λ_{em}: 520/30 nm; red: λ_{ex}: 564 nm, λ_{em}: 610/30 nm. **d**, Fluorescence spectra of the incubation medium of $VHL^{-/-}$ transgenic zebrafish or wide type zebrafish after 48 h treatment of JW40 (50 µM) showing an enhancement of the resorufin signal, which is due to the stronger NTR uncaging of $VHL^{-/-}$ larvae.

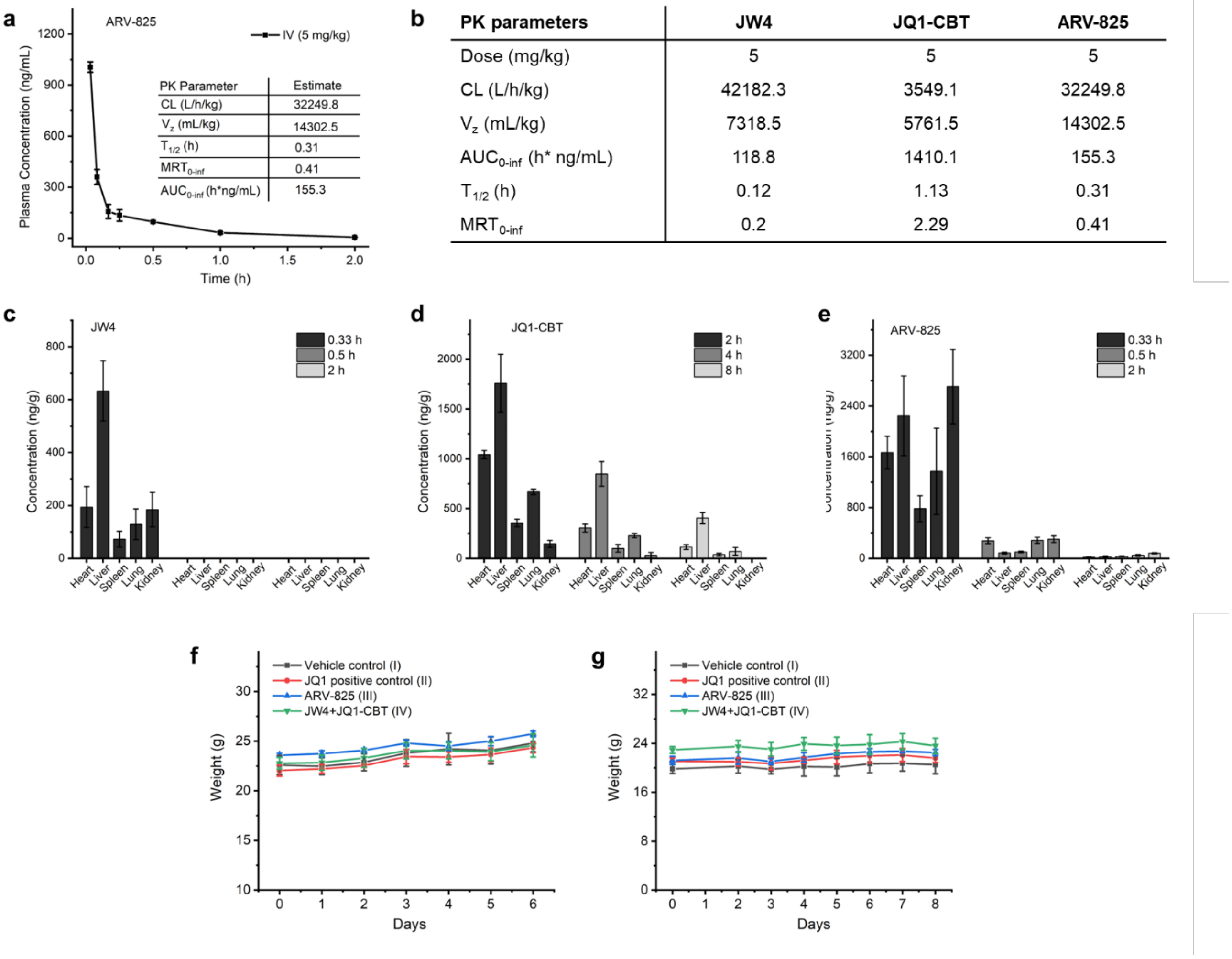

Fig. S11. Pharmacokinetic analysis and body weight of tumour bearing mouse during treatments. a, Pharmacokinetic (PK) analysis of ARV-825 in mouse. b, Calculated pharmacokinetic parameters for i.v. administration of JW4, JQ1-CBT and ARV-825. CL, clearance; V_z, volume in steady-state; $T_{1/2}$, half-life; MRT_{inf}, mean residence time extrapolated to infinity; AUC, area under the curve. c-e, Biodistribution of the JW4 (c) JQ1-CBT (d), and ARV-825 (e) at different time. Data represent the mean ± s.d. (n = 4). f, Body weight of the melanoma tumour bearing mouse under different treatments as indicated for 6 days. g, Body weight of the HeLa tumour bearing mouse under different treatments as indicated for 8 days.

Table S1. Details of human primers used for qRT-PCR

Gene (Human)	Forward Primer Sequence	Reverse Primer Sequence
C-MYC	5'-CCTGGTGCTCCATGAGGAGAC-3'	5'-CAGACTCTGACCTTTTGCCAGG-3'
VEGFA	5'-TTGCCTTGCTGCTCTACCTCCA-3'	5'-GATGGCAGTAGCTGCGCTGATA-3'
HIF1A	5'-TATGAGCCAGAAGAACTTTTAGGC-3'	5'-CACCTCTTTTGGCAAGCATCCTG-3'
GAPDH	5' GGTCTCCTCTGACTTCAACA 3'	5' AGCCAAATTCGTTGTCATAC 3'

General chemical synthetic procedures. Reagent-grade chemical reagents were purchased from Sigma-Aldrich, Thermo Fisher Scientific, MedChemExpress, and InnoChem. All chemical reactions were performed at ambient condition unless otherwise stated. Reactions were monitored by different means. Thin layer chromatography (TLC) was performed on TLC silica gel 60 F_{254} glass plates covered with approx. 0.2 mm silica gel. Ultraviolet light as the medium of TLC visualization. Mass spectra were measured on a ThermoFinnigan LCQ Fleet MS instrument and a ThermoFinnigan LCQ Deca XP MAX instrument for electrospray ionization (ESI) measurements. Compounds were purified over high-performance liquid chromatography (HPLC, Shimadzu) system using Alltima C-18 column of 250×10 mm at a flow rate of 3 mL min^{-1}. Purified compounds in H_2O were solidified in N_2 liquid and were freeze-dried in Labconco™ FreeZone™ 2.5 L Freeze Dryer.

Instrumentation for chemical structural elucidation. Proton NMR (^{1}H-NMR) and proton-decoupled carbon-13 NMR ($^{13}C\{^{1}H\}$-NMR) spectra were measured on a Bruker Avance III 400 (BBFO 400) Ultrashield Plus 400 MHz magnet with auto-tunable BBFO probe (5 mm). Spectra were recorded in accordance with the downfield ppm of tetramethylsilane. Calibration was performed with reference to the deuterium NMR solvent (CD_3OD: 3.31 [methanol], DMSO-d_6: 2.50 [dimethyl sulphoxide]) and carbon NMR solvent (CD_3OD: 49.00 [methanol], DMSO-d_6: 39.52 [dimethyl sulphoxide]). MestReNova (v14.0) was used to process all the molecular NMR analysis. The relative number of protons were integrated, and the coupling constants were unified as Hertz (Hz). Chemical shifts (δ) of molecular structure were reported, multiplicity (s, singlet; d, doublet; t, triplet; q, quartet; p, pentet; m, multiplet),

1. Preparation of varying linker length clicked-PROTAC molecule.

1.1) Synthesis of J242 and J252.

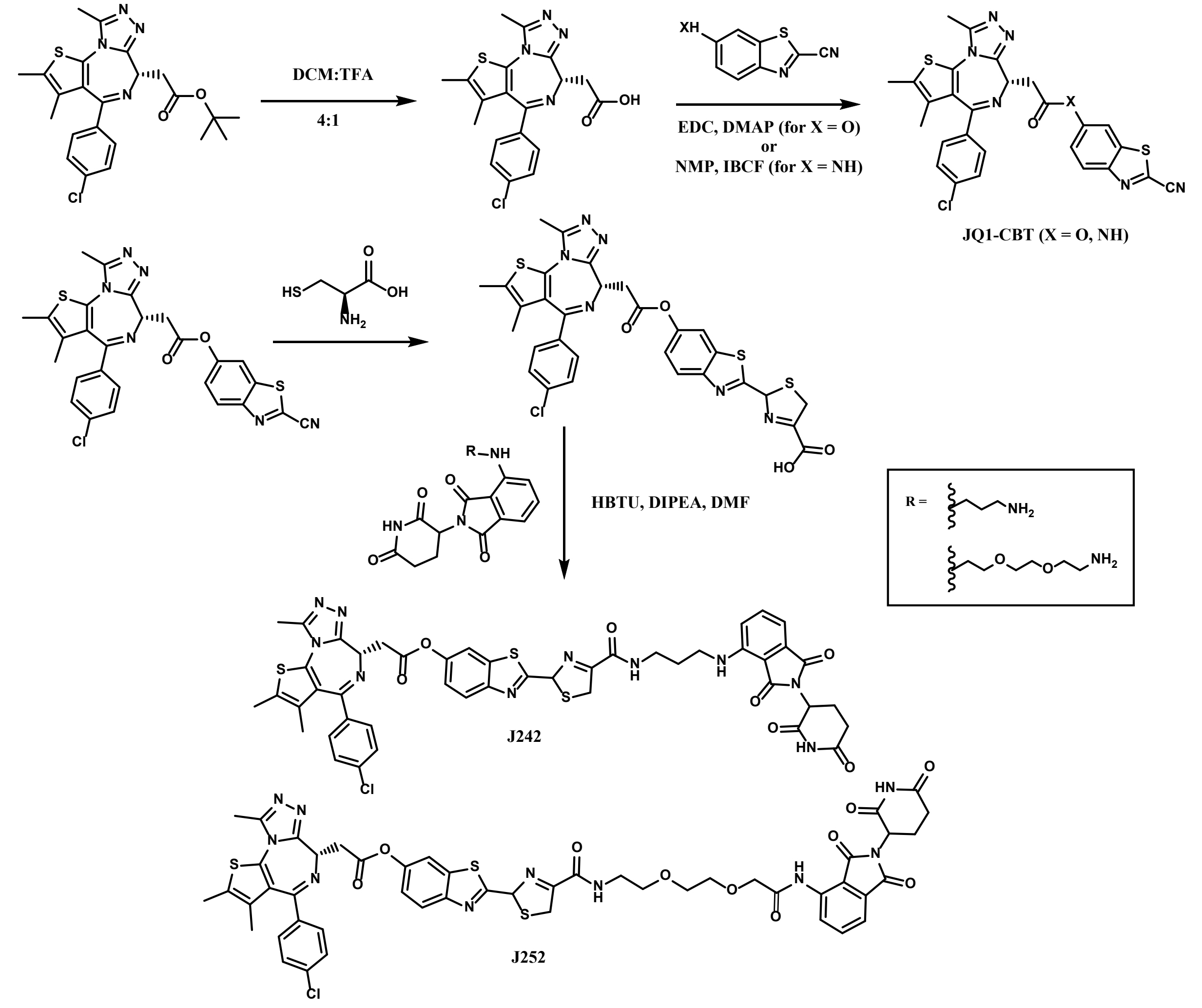

Figure S12. Synthesis of J242 and J252

1.1.1) Synthesis of JQ1-COOH.

To a 50-mL round bottom flask was added (+)-JQ1 (40 mg, 0.0998 mmol), which is subsequently dissolved in a mixture of 1:1 – DCM:TFA (4 mL). The reaction was stirred at r.t for 20 minutes, which was then extracted with DI water (1x) and DCM (2x), dried in $MgSO_4$, filtered and evaporated in vacuo to yield the deprotected-(+)-JQ-1. The organic extract was used for subsequent synthetic steps without further purification. ESI-MS: $[M]^+$ 401.20

1.1.2) Synthesis of JQ1-CBT.

To the DCM dissolution of deprotected-(+)-JQ-1 (18 mg, 0.045 mmol) was stirred together with 4-dimethylaminopyridine (2.7 mg, 0.0225 mmol) and 6-hydroxybenzothiazole-2-carbonitrile (31.7 mg, 0.18 mmol) for 30 minutes at room temperature. Thereafter, 1-ethyl-3-(3-dimethylaminopropyl)carbodiimide (9.4 mg, 0.049 mmol) pre-dissolved in DMF was dropwise added into the solution mixture and was left to stir for 4 hours at room temperature. The resultant crude product was purified over reversed-phase preparative HPLC (30 - 100%, v/v, $MeCN/H_2O$) and was lyophilized to yield JQ1-CBT (X = O) as a pale-yellowish solid (14.8 mg, 58.8%). ^{1}H NMR

(400 MHz, Methanol-d4) δ 8.25 (d, J = 9.0 Hz, 1H), 8.05 (d, J = 2.3 Hz, 1H), 7.54 (dd, J = 9.0, 2.3 Hz, 1H), 7.49 (d, J = 8.6 Hz, 2H), 7.44 (d, J = 8.7 Hz, 2H), 4.74 (t, J = 7.2 Hz, 1H), 3.92 – 3.85 (d, 2H), 2.72 (s, 3H), 2.45 (s, 3H), 1.70 (s, 3H). ^{13}C NMR (100 MHz, MeOD) δ 171.99, 166.60, 156.60, 152.39, 152.26, 151.45, 139.41, 138.16, 138.01, 137.73, 133.61, 133.45, 132.01, 131.91, 131.35, 129.90, 126.58, 124.25, 116.45, 113.83, 53.62, 37.51, 14.39, 12.51, 10.05. ESI-MS: $[M+H]^+$ 559.28. HRMS: *m/z*: $[M+H]^+$ calcd for $C_{27}H_{20}N_6O_2S_2Cl$: 559.0778; found: 559.0773. For JQ1-CBT (X=NH): to the THF dissolution of deprotected (+)-JQ-1 (10 mg, 0.025 mmol) was stirred together with isobutyl chloroformate (IBCF) (10.24 mg, 0.075 mmol) and N -methyl morpholine (NMP) 15.17 mg, 0.15 mmol) under N_2 at 0 °C for 30 minutes. Thereafter, 2-cyano-6-aminobenzothiazole (17.5 mg, 0.1 mmol) was added dropwise into the mixture and was stirred overnight to room temperature. The resultant crude product was purified over reversed-phase preparative HPLC (30 - 100%, v/v, MeCN/H_2O) and was lyophilized to yield JQ1-CBT (X = NH) as a pale-brown solid (5.3 mg, 38.1%). ^{1}H NMR (400 MHz, DMSO-d6) δ 10.85 (s, 1H), 8.78 (d, J = 2.0 Hz, 1H), 8.22 (d, J = 9.0 Hz, 1H), 7.79 (dd, J = 9.0, 2.1 Hz, 1H), 7.47 (d, J = 8.8 Hz, 2H), 7.41 (d, J = 8.7 Hz, 2H), 4.63 (dd, J = 7.8, 6.5 Hz, 1H), 3.59 (d, J = 13.5 Hz, 2H), 2.61 (s, 3H), 2.42 (s, 3H), 1.63 (s, 3H). ^{13}C NMR (400 MHz, DMSO) δ 169.87, 163.81, 155.44, 150.44, 148.06, 140.09, 137.25, 137.15, 135.76, 135.41, 132.75, 131.31, 130.60, 130.37, 130.05, 128.98, 125.33, 121.18, 114.05, 111.65, 54.13, 39.19, 14.51, 13.16, 11.74. ESI-MS: $[M+H]^+$ 558.30. HRMS: m/z: $[M+H]^+$ calcd for C27H20N7OS2Cl: 558.0938; found: 558.0941.

1.1.3) Synthesis of JQ1-CBT-Cys.

To DMF-dissolved JQ1-CBT (4 mg, 0.0072 mmol) was added L-cysteine (8 mg, 0.066 mmol) in solution of H_2O with the solvent proportion of 1:1. The reaction was stirred at room temperature for 2 hours to favour the reaction completion. The resultant crude product was extracted with DI water (1x) and DCM (2x), dried in $MgSO_4$, filtered and evaporated *in vacuo* to yield J240. The organic extract was used in subsequent step without further purification. ^{1}H NMR (400 MHz, MeOD) δ 8.13 (d, *J* = 8.0 Hz, 1H), 7.93 (d, *J* = 2.2 Hz, 1H), 7.50 (d, *J* = 8.0 Hz, 2H), 7.46 – 7.39 (m, 3H), 5.43 (t, *J* = 9.2 Hz, 1H), 4.77 (t, *J* = 7.2 Hz, 1H), 3.87 (d, *J* = 7.2 Hz, 2H), 3.80 (dd, *J* = 9.2, 2.3 Hz, 2H), 2.75 (s, 3H), 2.46 (s, 3H), 1.71 (s, 3H).^{13}C NMR (101 MHz, MeOD) δ 173.15, 171.09, 167.52, 166.67, 162.75, 156.60, 154.71, 152.31, 151.15, 138.24, 138.00, 137.90, 133.69, 133.42, 132.10, 132.06, 131.39, 129.92, 125.83, 122.93, 116.37, 79.59, 54.77, 37.45, 35.97, 14.39, 12.93, 11.57. ESI-MS: $[M]^+$ 663.05.

1.1.4) Synthesis of J242.

To synthesize J242, the crude J240 (4 mg, 0.00603 mmol) was added HBTU (18.36 mg, 0.0484 mmol) and the reaction was left to stir for 30 mins at room temperature. After that, pomalidomide-C_3-NH_2 (2.2 mg, 0.00663 mmol) was dissolved in DMF and was dropwise added into the reaction mixture, with subsequent addition of N,N-diisopropylethylamine (8.4 µL, 0.0484 mmol) to react for 6 hours at ambient condition. The resulting product was purified over reversed-phase preparative HPLC (30 - 100%, v/v, MeCN/H_2O) and was lyophilized to yield a yellowish solid (2.2 mg, 37.4%). ^{1}H NMR (600 MHz, DMSO) δ 11.08 (s, 1H), 8.27 (t, *J* = 5.7 Hz, 1H), 8.22 (d, *J* = 8.9 Hz, 1H), 8.06 (t, *J* = 2.1 Hz, 1H), 7.52 (d, *J* = 8.9 Hz, 2H), 7.48 (d, *J* = 8.5 Hz, 2H), 7.40 (dd, *J* = 8.9, 2.3 Hz, 1H), 7.08 (d, *J* = 8.6 Hz, 1H), 7.00 (d, *J* = 6.7 Hz, 1H), 5.35 (t, *J* = 9.3 Hz, 1H), 5.04 (dd, *J* = 12.8, 5.4 Hz, 2H), 4.67 – 4.62 (m, 1H), 3.84 – 3.69 (m, 1H), 3.27 – 3.23 (m, 2H), 2.90 – 2.84 (m, 2H), 2.63 (s, 3H), 2.61 – 2.57 (m, 1H), 2.41 (s, 3H), 2.04 – 2.00 (m, 1H), 1.77 – 1.73 (m, 2H), 1.63 (s, 3H). ^{13}C NMR (151 MHz, DMSO) δ 172.86, 170.14, 169.63, 169.11, 168.84, 167.34, 164.27, 163.67, 161.31, 154.60, 150.52, 150.18, 149.17, 146.28, 136.62, 136.27, 135.45, 132.40, 130.91, 130.29, 129.95, 129.51, 128.62, 124.83, 121.97, 118.12, 117.13, 115.78, 110.42, 109.21, 79.35, 53.46, 48.55, 34.74, 31.00, 28.60, 22.18, 14.09, 12.70, 11.34. ESI-MS: $[M+H]^+$ 975.29. HRMS: *m/z*: $[M+H]^+$ calcd for $C_{46}H_{40}N_{10}O_7S_3Cl$: 975.1932; found: 975.1908.

1.1.5) Synthesis of J252.

Synthesis of J252 follows the same procedure as J242, please refer to the synthetic protocol as shown above. ^{1}H NMR (600 MHz, DMSO) δ 11.14 (s, 1H), 10.33 (s, 1H), 8.68 (d, *J* = 8.4 Hz, 1H), 8.19 (d, *J* = 8.9 Hz, 1H), 8.00 (t, *J* = 2.4

Hz, 1H), 7.84 – 7.78 (m, 1H), 7.58 (dd, J = 7.3, 3.6 Hz, 1H), 7.59 – 7.57 (m, 4H), 7.39 – 7.35 (m, 1H), 5.33 (td, J = 9.1, 2.4 Hz, 1H), 5.16 (dd, J = 12.9, 5.4 Hz, 1H), 4.66 (t, J = 7.2 Hz, 1H), 4.19 (s, 2H), 3.86 – 3.69 (m, 8H), 3.54 – 3.52 (m, 2H), 3.36 – 3.30 (m, 2H), 2.99 – 2.96 (m, 1H), 2.93 – 2.83 (m, 2H), 2.64 (s, 3H), 2.41 (s, 3H), 2.13 – 2.02 (m, 1H), 1.63 (s, 3H). ^{13}C NMR (151 MHz, DMSO) δ 172.77, 169.78, 169.53, 169.35, 169.16, 168.26, 166.70, 164.33, 163.76, 161.20, 154.52, 150.48, 150.32, 149.12, 136.52, 136.10, 135.95, 135.49, 132.24, 131.28, 131.09, 130.32, 130.00, 129.62, 128.61, 124.79, 124.36, 121.91, 118.31, 116.02, 115.69, 79.14, 70.74, 70.20, 69.51, 68.84, 53.39, 48.99, 48.62, 36.57, 30.96, 28.26, 21.99, 14.09, 12.72, 11.33. ESI-MS: $[M+H]^+$ 1063.32. HRMS: *m/z*: $[M+H]^+$ calcd for $C_{49}H_{44}N_{10}O_{10}S_3Cl$: 1063.2093; found: 1063.2057

1.1.6) Synthesis of inactive stereoisomer (-)-JQ1-CBT

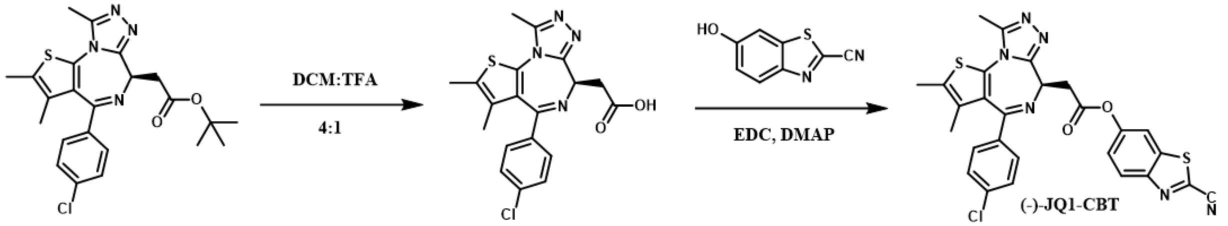

To the DCM dissolution of deprotected (-)-JQ-1 (18 mg, 0.045 mmol) was stirred together with 4-dimethylaminopyridine (2.7 mg, 0.0225 mmol) and 6-hydroxybenzothiazole-2-carbonitrile (31.7 mg, 0.18 mmol) for 30 minutes at room temperature. Thereafter, 1-ethyl-3-(3-dimethylaminopropyl)carbodiimide (9.4 mg, 0.049 mmol) pre-dissolved in DMF was dropwise added into the solution mixture and was left to stir for 4 hours at room temperature. The resultant crude product was purified over reversed-phase preparative HPLC (30 - 100%, v/v, MeCN/H_2O) and was lyophilized to yield (-)-JQ1-CBT as a pale-yellowish solid (12.9 mg, 51.2%). ^{1}H NMR (400 MHz, DMSO-d_6) δ 8.33 (d, J = 9.0 Hz, 0H), 8.21 (d, J = 2.3 Hz, 0H), 7.56 – 7.44 (m, 2H), 4.65 (t, J = 7.2 Hz, 0H), 3.89 – 3.73 (m, 1H), 2.63 (s, 1H), 2.41 (s, 1H), 1.63 (s, 1H). ^{13}C NMR (400 MHz, DMSO) δ 169.92, 164.14, 154.99, 150.64, 150.58, 149.97, 138.20, 137.04, 136.85, 135.89, 132.80, 131.38, 130.71, 130.40, 129.96, 129.04, 125.87, 123.61, 116.43, 113.76, 53.85, 37.05, 14.52, 13.14, 11.76. ESI-MS: $[M+H]^+$ 559.17.

1.2) Synthesis of T208.

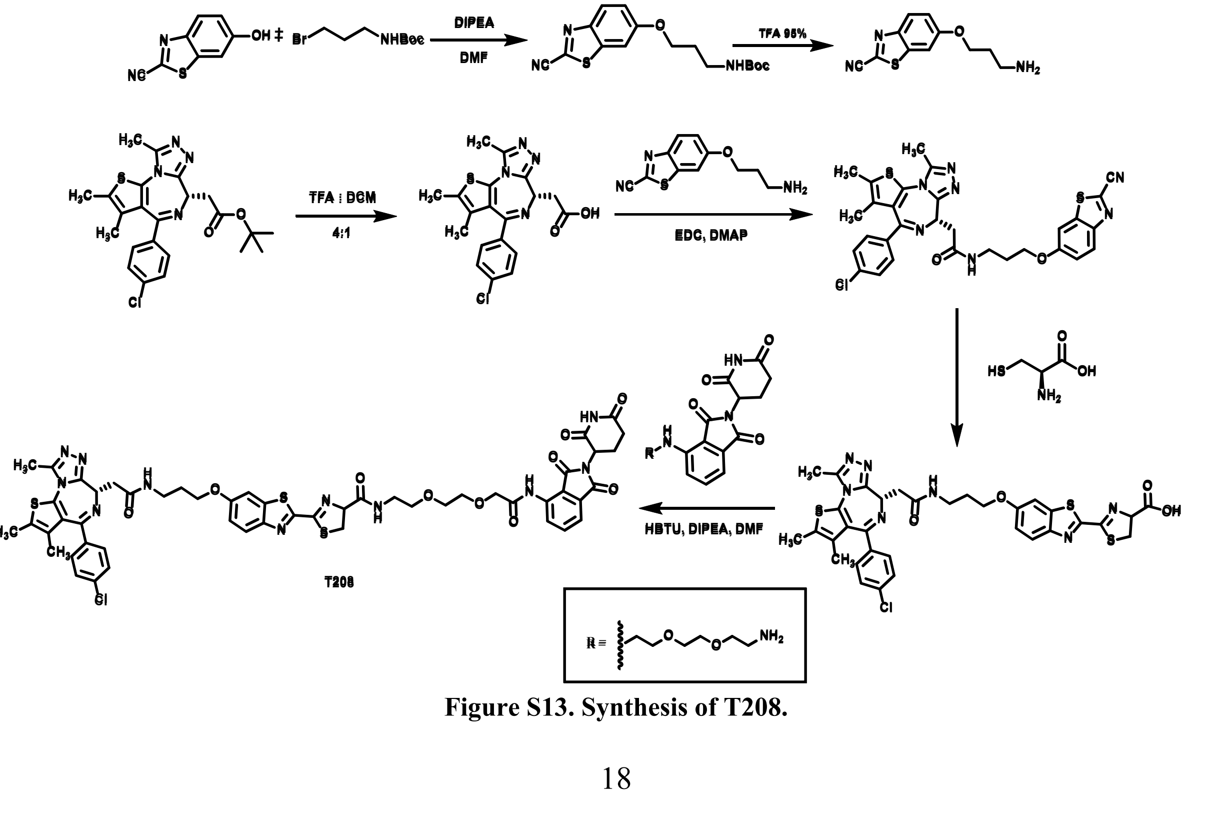

Figure S13. Synthesis of T208.

1.2.1) Synthesis of BocNH-C_3-CBT.

To a stirred solution of 6-hydroxybenzo[d]thiazole-2-carbonitrile (50 mg, 0.284 mmol) in DMF (1 mL), 2 M solution of *N*-Ethyldiisopropylamine in 1-methyl-2-pyrrolidinone (171 µl, 0.341 mmol) was added. The mixture was stirred for 10 min, then the solution of *tert*-butyl (3-bromopropyl)carbamate (134 mg, 0.570 mmol) in DMF (1 mL) was added to the mixture. The reaction solution was stirred at r.t for 16 h. The crude product was purified by preparative HPLC (30%-100% acetonitrile in H_2O). The product-containing fractions were dried by lyophilisation to afford **BocNH-C_3-CBT** as yellow solid (23.7 mg, 25%). ^1H NMR (400 MHz, Methanol-d_4) δ 8.04 (d, J = 9.1 Hz, 1H), 7.62 (d, J = 2.5 Hz, 1H), 7.28 (dd, J = 9.1, 2.5 Hz, 1H), 4.13 (t, J = 6.1 Hz, 2H), 3.26 (t, J = 6.8 Hz, 2H), 2.00 (p, J = 6.4 Hz, 2H), 1.42 (s, 9H). ^{13}C NMR (100 MHz, MeOD) δ 161.34, 158.56, 148.05, 138.98, 134.92, 126.40, 120.16, 114.19, 105.22, 80.00, 67.51, 54.79, 38.27, 30.55, 28.74. ESI-MS: $[M+H]^+$ 234.04

1.2.2) Synthesis of NH_2-C_3-CBT.

To a stirred solution of **BocNH-C_3-CBT** (23.7 mg, 71.0 µmol) in CH_2Cl_2 (1 mL), TFA (0.5 mL, 6.53 mmol) was added. The reaction mixture was stirred for 1 h at r.t.. The crude product was purified by preparative HPLC (30%-100% acetonitrile in H_2O). The product-containing fractions were dried by lyophilisation to afford **NH_2-C_3-CBT** as light yellow liquid (14.3 mg, 86%).^1H NMR (400 MHz, Methanol-d_4) δ 8.09 (d, *J* = 9.1 Hz, 1H), 7.69 (d, *J* = 2.5 Hz, 1H), 7.34 (dd, *J* = 9.1, 2.5 Hz, 1H), 4.25 (t, *J* = 5.8 Hz, 2H), 3.19 (t, *J* = 7.3 Hz, 2H), 2.22 (ddd, *J* = 13.0, 7.3, 5.7 Hz, 2H). ^{13}C NMR (100 MHz, MeOD) δ 160.79, 148.35, 138.98, 135.40, 126.57, 120.01, 114.12, 105.46, 67.08, 38.45, 28.22. ESI-MS: $[M+H]^+$ 234.04

1.2.3) Synthesis of JQ1-C_3-CBT: To a stirred solution of **NH_2-C_3-CBT** (14.3 mg, 61.3 µmol) and **JQ1-COOH** (29.5 mg, 73.6 µmol) in DMF (800 µL), 4-dimethylaminopyridine (0.750 mg, 6.13 µmol) was added into the solution. 1-Ethyl-3-(3-dimethylaminopropyl)carbodiimide (14.1 mg, 73.6 µmol) was then added into the solution. The reaction mixture was stirred for 16 h at r.t. The reaction mixture was stirred for 1 h at r.t. The crude product was purified by preparative HPLC (30%-100% acetonitrile in H_2O). The product-containing fractions were dried by lyophilisation to afford **JQ1-C_3-CBT** as light-yellow solid (11.7 mg, 31%). ^1H NMR (400 MHz, Methanol-d_4) δ 8.05 (d, *J* = 9.1 Hz, 1H), 7.64 (d, *J* = 2.5 Hz, 1H), 7.44 – 7.36 (m, 2H), 7.34 – 7.21 (m, 3H), 4.65 (dd, *J* = 8.7, 5.6 Hz, 1H), 4.27 – 4.10 (m, 2H), 3.58 – 3.36 (m, 4H), 2.69 (s, 3H), 2.48 – 2.37 (m, 3H), 2.11 (t, *J* = 6.4 Hz, 2H), 1.66 (s, 3H). ^{13}C NMR (100 MHz, MeOD) δ 173.56, 166.46, 162.06, 156.94, 152.57, 149.07, 139.02, 138.10, 137.78, 134.99, 133.52, 133.45, 132.11, 131.98, 131.29, 129.75, 126.44, 120.25, 113.57, 105.39, 66.58, 56.06, 40.87, 37.25, 30.59, 13.98, 12.48, 11.57. ESI-MS: $[M+H]^+$ 616.28

1.2.4) Synthesis of JQ1-C_3-CBT-Cys. To a stirred solution of **JQ1-C3-CBT** (11.7 mg, 19.0 µmol) in DMF (800 µL), *L*-cysteine (2.30 mg, 19.0 µmol) was added. The reaction mixture was heated to 40 ºC and stirred for 2 h. The reaction mixture was then cooled down to r.t and purified by preparative HPLC (30%-100% acetonitrile in H_2O). The product-containing fractions were dried by lyophilisation to afford **JQ1-C_3-CBT-Cys** as white solid (8.61 mg, 63%). ^1H NMR (400 MHz, MeOD) δ 7.92 (d, *J* = 12.0 Hz, 1H), 7.53 (d, *J* = 4.0 Hz, 1H), 7.39 (d, *J* = 8.0 Hz, 2H), 7.28 (d, *J* = 8.0 Hz, 2H), 7.18 (dd, *J* = 12.0 Hz, 4.0 Hz, 1H), 5.40 (t, *J* = 8.0 Hz, 1H), 4.64 (t, *J* = 8.0 Hz, 1H), 4.21 – 4.11 (m, 2H), 3.81 – 3.73 (m, 2H), 3.52 – 3.40 (m, 4H), 2.68 (s, 3H), 2.43 (s, 3H), 2.13 – 2.07 (m, 2H), 1.66 (s, 3H). ^{13}C NMR (101 MHz, MeOD) δ 173.27, 172.86, 167.63, 166.46, 160.24, 159.45, 156.94, 152.30, 148.85, 139.06, 138.13, 137.75, 133.50, 133.44, 132.12, 131.97, 131.27, 129.76, 125.78, 118.70, 105.84, 79.48, 67.33, 55.13, 38.72, 37.27, 35.88, 30.20, 14.38, 12.92, 11.57. ESI-MS: $[M+H]^+$ 720.29

1.2.5) Synthesis of T208. To a stirred solution of **JQ1-C_3-CBT-Cys** (4.60 mg, 6.39 µmol) and HBTU (3.63 mg, 9.59 µmol) in DMF (500 µL), **Poma-$(PEG)_2$-NH_2.HCl** (2.90 mg, 6.39 µmol) was added. 2 M solution of *N*-Ethyldiisopropylamine in 1-methyl-2-pyrrolidinone (4.75 µL, 9.59 µmol) was then added into the solution. The

reaction mixture was then stirred at r.t for 18 h. The crude product was purified by preparative HPLC (30%-100% acetonitrile in H_2O). The product-containing fractions were dried by lyophilisation to afford **T208** as white solid (3.07 mg, 43%). ESI-MS: $[M+H]^+$ 1120.32

^{1}H NMR (600 MHz, DMSO) δ 11.14 (s, 1H), 10.33 (s, 1H), 8.68 (dd, J = 8.4, 2.6 Hz, 1H), 8.34 (t, J = 5.6 Hz, 1H), 8.01 – 7.96 (m, 1H), 7.85 – 7.80 (m, 1H), 7.59 (d, J = 7.2 Hz, 1H), 7.45 – 7.36 (m, 4H), 7.21 – 7.14 (m, 1H), 5.29 (td, J = 9.3, 2.4 Hz, 1H), 5.18 – 5.11 (m, 1H), 4.53 (t, J = 7.1 Hz, 1H), 4.20 – 4.08 (m, 4H), 3.81 – 3.59 (m, 10H), 3.33 – 3.30 (m, 2H), 3.25 (t, J = 7.1 Hz, 2H), 2.94 – 2.84 (m, 2H), 2.61 (s, 3H), 2.39 (s, 3H), 2.12 – 2.02 (m, 2H), 1.97 – 1.94 (m, 2H), 1.58 (s, 3H).
^{13}C NMR (151 MHz, DMSO) δ 172.90, 172.31, 170.03, 169.91, 169.61, 169.52, 168.49, 166.93, 164.76, 163.44, 158.44, 155.39, 150.17, 147.26, 137.41, 136.93, 136.68, 136.05, 135.52, 132.43, 131.47, 131.03, 130.28, 130.13, 129.88, 128.63, 124.90, 124.48, 118.49, 117.55, 105.37, 79.21, 70.99, 70.41, 69.74, 69.04, 66.18, 54.09, 49.19, 37.87, 35.52, 34.84, 31.11, 29.07, 22.20, 21.06, 14.12, 12.76, 11.40.
HRMS: *m/z*: $[M+H]^+$ calcd for $C_{52}H_{51}N_{11}O_{10}S_3Cl$: 1120.2671; found: 1120.2678.

2. Preparation of nitroreductase (NTR)-responsive fragment molecule.

2.1) Pomalidomide-C3-NTR

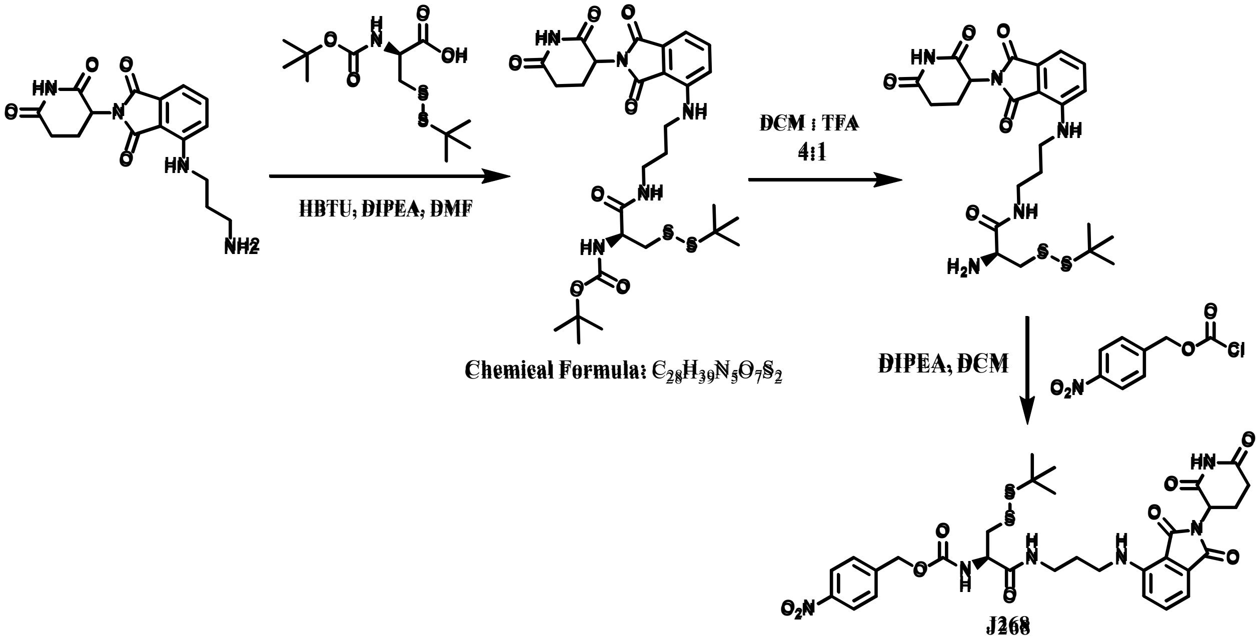

Figure S14. Synthesis of Pomalidomide-C3-NTR (J268).

2.1.1) Synthesis of J260

To a 50-mL round bottom flask was added Boc-Cys(StBu)-OH (6.75 mg, 0.0218 mmol) and HBTU (27.6 mg, 0.073 mmol) in DMF, which was then left to stir for 30 mins at room temperature. Thereafter, pomalidomide-C_3-NH_2 (6 mg, 0.0182 mmol) was dissolved in DMF and was dropwise added into the reaction mixture, followed by the addition of N,N-diisopropylethylamine (12.9 µL, 0.073 mmol) to stir at 50 °C for 4 hours. The resulting crude product was then purified over reversed-phase preparative HPLC (30 - 100%, v/v, MeCN/H_2O) and was lyophilized to yield J260 as a yellowish solid (6.7 mg, 59.3%). H-NMR and C-NMR ESI-MS: $[M]^+$ 622.15. ^1H NMR (400 MHz, Methanol-d4) δ 7.52 (dd, J = 8.5, 7.0 Hz, 1H), 7.03 (t, J = 7.7 Hz, 2H), 5.05 (dd, J = 12.5, 5.4 Hz, 1H), 4.31 (t, J = 7.0 Hz, 1H), 3.37 (t, J = 6.8 Hz, 2H), 3.35 – 3.26 (m, 4H), 3.10 (dd, J = 13.4, 5.8 Hz, 1H), 3.00 – 2.80 (m, 2H), 2.79 – 2.65 (m, 2H), 2.11 (ddq, J = 10.3, 5.5, 2.2 Hz, 1H), 1.84 (p, J = 6.8 Hz, 2H), 1.44 (s, 9H), 1.33 (s, 9H). ^{13}C NMR (100 MHz, MeOD) δ 174.69, 173.33, 171.63, 170.68, 169.32, 157.54, 148.04, 137.23, 133.92, 118.07, 111.84, 111.19, 80.90, 55.80, 50.18, 43.48, 40.88, 37.95, 32.22, 30.21, 30.03, 28.70, 23.79. ESI-MS: $[M]^+$ 622.15 $[M+2H]^{2+}$ 1242.68.

2.1.2) Synthesis of J264

The as-synthesized J260 was dissolved in a solution mixture of DCM:TFA (4:1) and was stirred for 20 mins at room temperature. The crude was then extracted with DI H_2O (1x) and DCM (2x), dried in $MgSO_4$, filtered and evaporated in vacuo. Further purification was performed by reversed-phase preparative HPLC (30 - 100%, v/v, MeCN/H_2O) and was lyophilized to yield J264 as a yellowish solid (5.4 mg, 96.3%). H-NMR and C-NMR ESI-MS: $[M]^+$ 522.32. ^1H NMR (400 MHz, Methanol-d4) δ 7.56 (dd, J = 8.6, 7.1 Hz, 1H), 7.07 (dd, J = 7.8, 4.5 Hz, 2H), 5.06 (dd, J = 12.5, 5.5 Hz, 1H), 4.06 (dd, J = 7.2, 6.1 Hz, 1H), 3.42 (dd, J = 6.9, 4.6 Hz, 2H), 3.40 – 3.33 (m, 2H), 3.27 – 3.05 (m, 2H), 2.93 – 2.81 (m, 1H), 2.80 – 2.65 (m, 2H), 2.18 – 2.06 (m, 1H), 1.90 (p, J = 6.7 Hz, 2H), 1.35 (s, 9H). ^{13}C NMR (100 MHz, MeOD) δ 174.67, 171.69, 170.79, 169.27, 168.64, 148.02, 137.31, 133.97, 118.01, 112.00, 111.30, 54.03, 50.19, 42.33, 41.00, 38.43, 32.19, 30.07, 29.82, 23.80. ESI-MS: $[M]^+$ 522.32.

2.1.3) Synthesis of J268

To the synthesized J264 (3.2 mg, 0.00614 mmol) was added with solution mixture of p-nitrobenzyl chloroformate (2.65 mg, 0.0123 mmol) and N,N-diisopropylethylamine (4.4 µL, 0.0246 mmol) dissolved in DCM. The reaction was completed upon 2 hours of stirring at room temperature. The resultant crude product was the purified over reversed-phase preparative HPLC (30 - 100%, v/v, MeCN/H_2O) and was lyophilized to yield J268 as a yellowish solid (2.8 mg, 65.1%). H-NMR and C-NMR ESI-MS: $[M]^+$ 701.14. ^{1}H NMR (400 MHz, Methanol-d_4) δ 8.19 – 8.14 (m, 2H), 7.57 (d, J = 8.5 Hz, 2H), 7.50 (dd, J = 8.6, 7.1 Hz, 1H), 7.00 (dd, J = 7.9, 6.0 Hz, 2H), 5.23 (d, J = 2.5 Hz, 2H), 5.05 (dd, J = 12.5, 5.4 Hz, 1H), 4.43 – 4.32 (m, 1H), 3.05 (ddd, J = 70.6, 13.5, 7.1 Hz, 2H), 2.89 – 2.79 (m, 1H), 2.79 – 2.65 (m, 2H), 2.12 (td, J = 7.0, 6.4, 3.0 Hz, 1H), 1.83 (h, J = 6.6 Hz, 2H), 1.32 (s, 9H). ^{13}C NMR (100 MHz, MeOD) δ 174.73, 173.01, 171.66, 170.67, 169.34, 157.82, 148.81, 147.98, 145.93, 137.22, 133.91, 129.01, 128.25, 124.56, 124.45, 118.03, 111.82, 111.13, 66.36, 56.27, 50.18, 43.12, 40.85, 37.98, 32.21, 30.20, 29.99, 23.79. ESI-MS: $[M]^+$ 701.14.

2.2) Pomalidomide-PEG-NTR

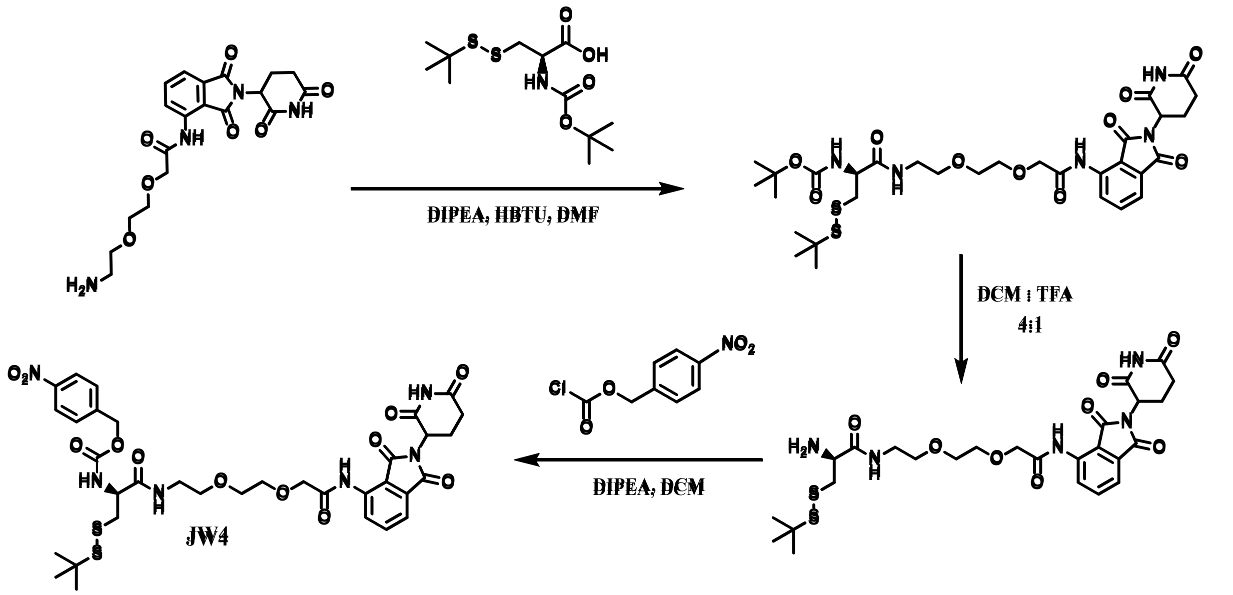

Figure S15. Synthesis of JW4.

2.2.1) Synthesis of J262.

To a 50-mL round bottom flask was added Boc-Cys(StBu)-OH (8.9 mg, 0.0287 mmol), HBTU (36.3 mg, 0.096 mmol) in DMF, which was then left to stir for 30 mins at room temperature. Thereafter, pomalidomide-PEG$_2$-NH_2 (10 mg, 0.0239 mmol) was dissolved in DMF and was dropwise added into the reaction mixture. Subsequently, N,N-diisopropylethylamine (17 µL, 0.096 mmol) was added dropwise and the reaction mixture was left to react at 50 °C for 4 hours. The resulting crude product was then purified over reversed-phase preparative HPLC (30 - 100%, v/v, MeCN/H_2O) and was lyophilized to yield J262 as a white solid (9.3 mg, 54.8%). ^{1}H NMR (400 MHz, Methanol-d4) δ 8.81 (d, J = 8.5 Hz, 1H), 7.81 (t, J = 7.9 Hz, 1H), 7.61 (d, J = 7.3 Hz, 1H), 5.16 (dd, J = 12.6, 5.4 Hz, 1H), 4.30 (s, 1H), 4.22 (s, 2H), 3.88 – 3.71 (m, 4H), 3.61 (t, J = 5.6 Hz, 2H), 3.42 (tq, J = 19.2, 6.9, 6.2 Hz, 2H), 3.09 (dd, J = 13.5, 5.4 Hz, 1H), 2.95 – 2.84 (m, 2H), 2.83 – 2.64 (m, 2H), 2.28 – 2.11 (m, 1H), 1.43 (s, 9H), 1.30 (s, 9H). ^{13}C NMR (100 MHz, MeOD) δ 174.71, 173.14, 171.60, 171.35, 169.90, 168.34, 157.56, 137.50, 137.39, 132.99, 125.96, 119.70, 117.89, 80.99, 72.63, 71.74, 71.25, 70.49, 55.58, 54.79, 50.58, 43.56, 40.44, 32.13, 30.18, 28.67, 23.65. ESI-MS: $[M+H]^+$ 710.09 $[M+Na]^+$ 732.30.

2.2.2) Synthesis of J266.

The as-synthesized J262 was dissolved in a solution mixture of DCM:TFA (4:1) and was stirred for 20 mins at room temperature. The crude was then evaporated *in vacuo* and was further purified by reversed-phase preparative HPLC (30 - 100%, v/v, MeCN/H_2O) and was lyophilized to yield J266 as a white solid (7.3 mg, 91.4%). ^1H NMR (400 MHz, Methanol-d4) δ 8.82 (d, J = 8.4 Hz, 1H), 7.82 (t, J = 7.9 Hz, 1H), 7.62 (d, J = 7.3 Hz, 1H), 5.16 (dd, J = 12.3, 5.0 Hz, 1H), 4.22 (s, 2H), 4.10 – 4.00 (m, 1H), 3.88 – 3.74 (m, 4H), 3.64 (d, J = 6.0 Hz, 2H), 3.57 – 3.34 (m, 2H), 3.25 – 3.01 (m, 2H), 2.90 (ddd, J = 18.5, 13.8, 5.2 Hz, 1H), 2.83 – 2.67 (m, 2H), 2.18 (d, J = 12.7 Hz, 1H), 1.32 (s, 9H). ^{13}C NMR (100 MHz, MeOD) δ 174.72, 171.56, 169.98, 168.55, 168.22, 137.55, 137.42, 133.04, 125.93, 119.71, 117.88, 72.62, 71.77, 71.38, 70.37, 53.88, 50.58, 42.22, 40.81, 32.12, 30.14, 30.04, 23.64. ESI-MS: $[M+H]^+$ 610.30.

2.2.3) Synthesis of JW4

To the synthesized J266 (10 mg, 0.0164 mmol) was added with solution mixture of p-nitrobenzyl chloroformate (7.1 mg, 0.0328 mmol) and N,N-diisopropylethylamine (22.8 µL, 0.1312 mmol) dissolved in DCM. The reaction was completed upon 2 hours of stirring at room temperature. The resultant crude product was purified over reversed-phase preparative HPLC (30 - 100%, v/v, MeCN/H_2O) and was lyophilized to yield JW4 as a white solid (5.4 mg, 41.9%). ^1H NMR (400 MHz, Methanol-d4) δ 8.78 – 8.69 (m, 1H), 8.16 – 8.10 (m, 2H), 7.74 (dd, J = 8.5, 7.3 Hz, 1H), 7.54 (d, J = 4.8 Hz, 1H), 7.54 – 7.50 (m, 2H), 5.15 – 5.13 (m, 1H), 4.55 (s, 2H), 4.35 (dt, J = 9.1, 4.9 Hz, 1H), 4.16 (s, 2H), 3.79 – 3.67 (m, 4H), 3.55 (d, J = 5.5 Hz, 2H), 3.38 – 3.33 (m, 2H), 3.17 – 3.06 (m, 2H), 2.93 – 2.84 (m, 2H), 2.82 (d, J = 5.3 Hz, 1H), 2.76 – 2.66 (m, 2H), 2.63 (s, 1H), 2.18 – 2.09 (m, 1H), 1.26 – 1.23 (m, 9H). ^{13}C NMR (100 MHz, MeOD) δ 173.15, 171.35, 170.10, 169.93, 168.56, 166.85, 156.36, 147.45, 144.40, 136.18, 135.90, 131.66, 127.68, 126.76, 124.50, 123.05, 118.17, 116.50, 106.68, 71.23, 70.38, 69.87, 69.07, 64.94, 54.69, 49.21, 41.95, 39.12, 30.76, 28.78, 22.30. ESI-MS: $[M+H]^+$ 789.15. HRMS: *m/z*: $[M+H]^+$ calcd for $C_{34}H_{41}N_6O_{12}S_2$: 789.2224; found: 789.2234.

ESI-MS Spectra
Synthesis of J242 and J252

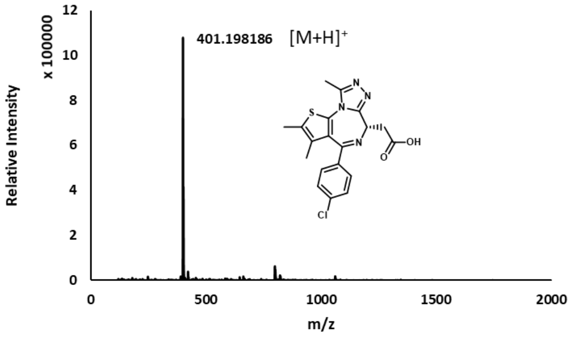

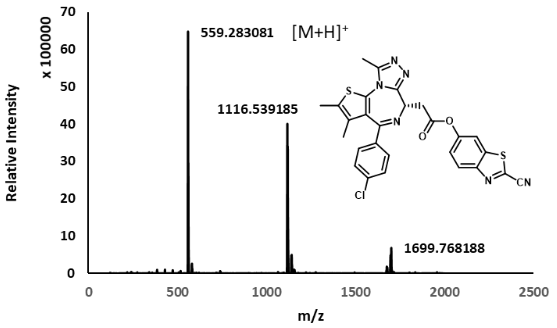

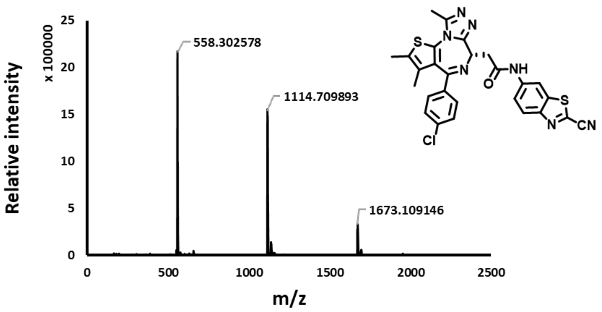

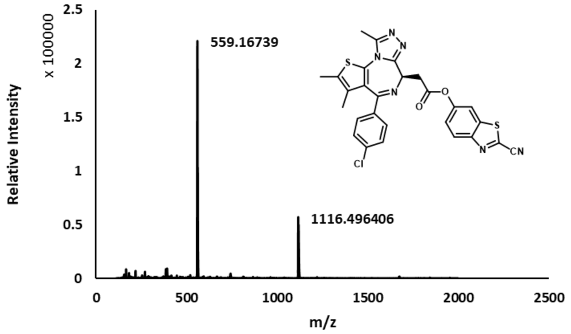

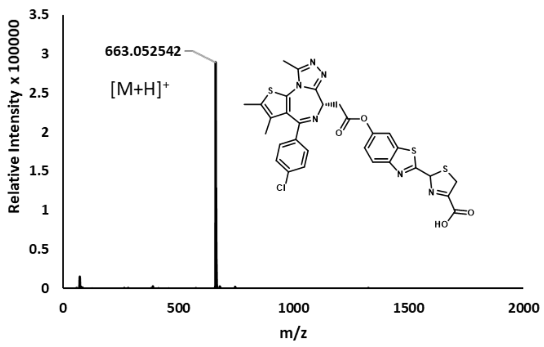

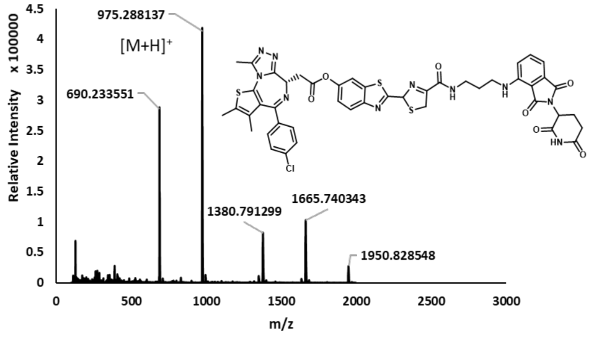

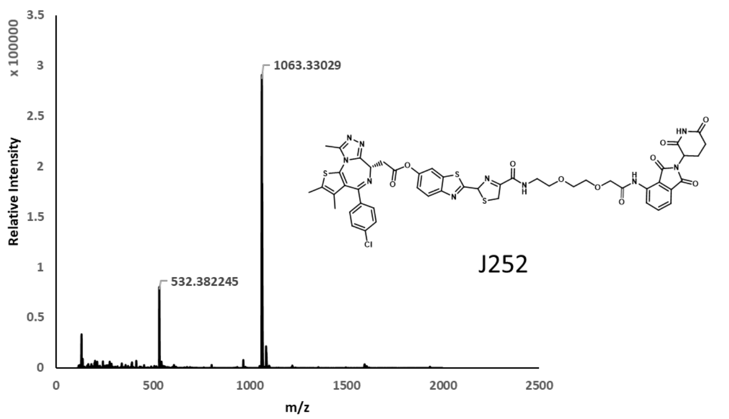

Synthesis of T208

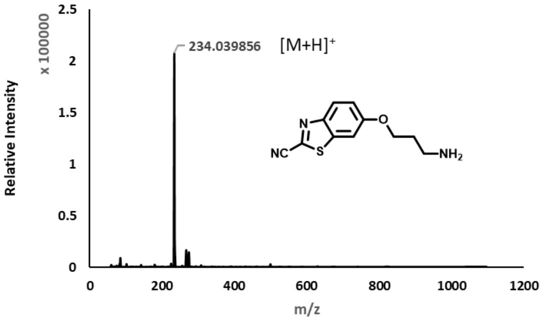

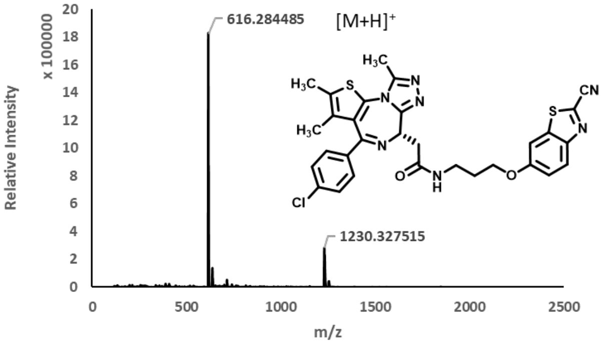

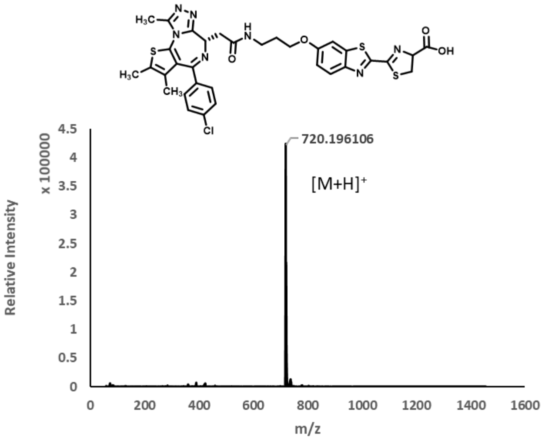

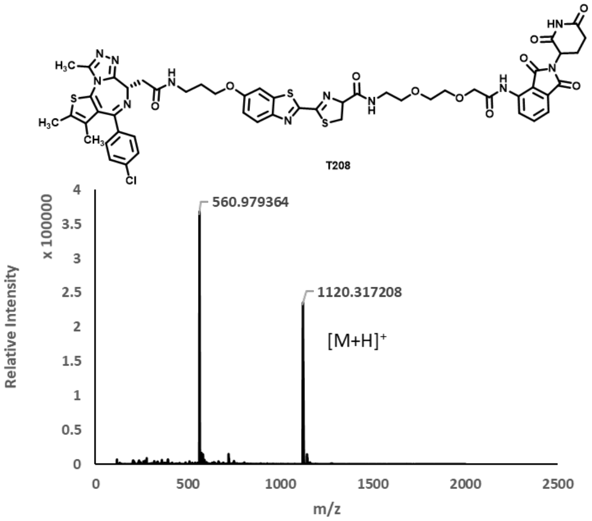

Synthesis of nitroreductase-responsive fragment molecule.

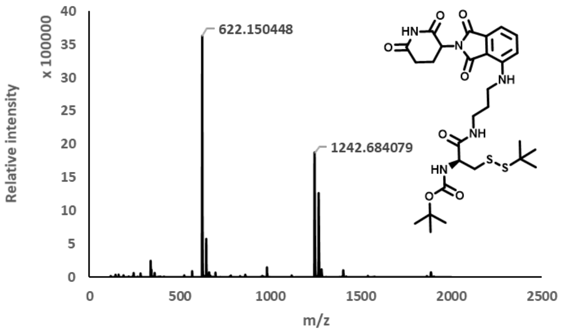

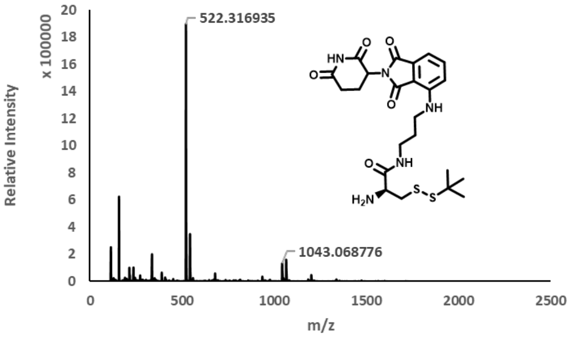

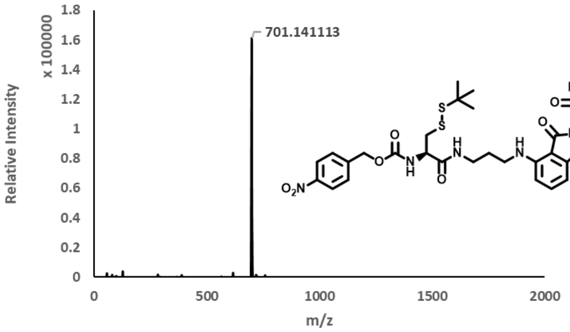

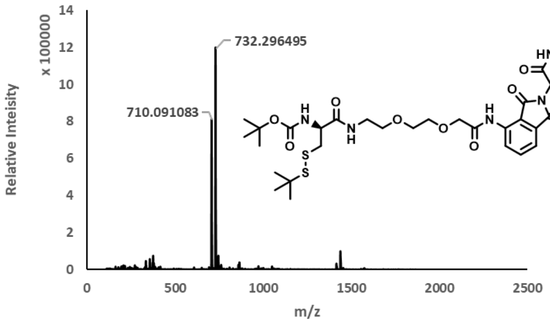

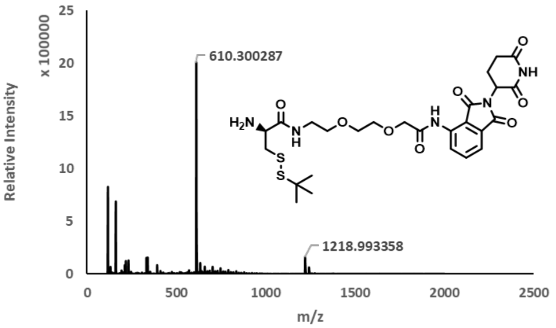

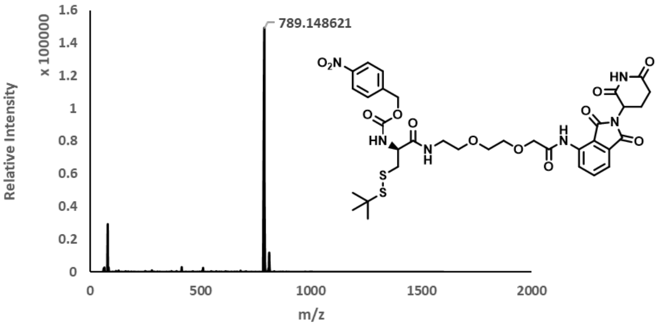

HRMS Spectra

JW4

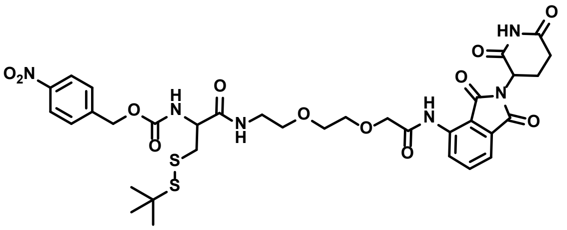

HRMS: *m/z*: $[M+H]^+$ calcd for $C_{34}H_{41}N_6O_{12}S_2$: 789.2224; found: 789.2234

Elemental Composition Report Page 1

Single Mass Analysis
Tolerance = 5.0 PPM / DBE: min = -1.5, max = 50.0
Element prediction: Off
Number of isotope peaks used for i-FIT = 2

Monoisotopic Mass, Even Electron Ions
266 formula(e) evaluated with 1 results within limits (all results (up to 1000) for each mass)
Elements Used:
C: 0-34 H: 0-41 N: 0-6 O: 0-12 S: 0-2
C34H40N6O12S2
GK-jw4 5 (0.121) Cm (5:8)

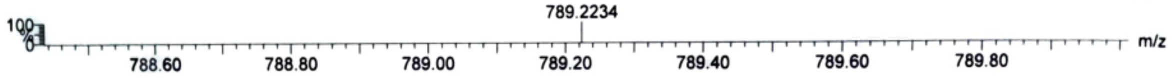

Minimum:				-1.5				
Maximum:		5.0	5.0	50.0				
Mass	Calc. Mass	mDa	PPM	DBE	i-FIT	Norm	Conf(%)	Formula
789.2234	789.2224	1.0	1.3	17.5	37.5	n/a	n/a	C34 H41 N6 O12 S2

JQ1-CBT (X = O)

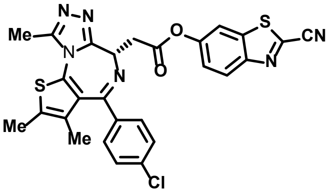

HRMS: *m/z*: $[M+H]^+$ calcd for $C_{27}H_{20}N_6O_2S_2Cl$: 559.0778; found: 559.0773

Page 1

Elemental Composition Report

Single Mass Analysis

Tolerance = 5.0 PPM / DBE: min = -1.5, max = 50.0
Element prediction: Off
Number of isotope peaks used for i-FIT = 2

Monoisotopic Mass, Even Electron Ions
52 formula(e) evaluated with 1 results within limits (all results (up to 1000) for each mass)
Elements Used:
C: 0-27 H: 0-20 N: 0-6 O: 0-2 S: 0-2 Cl: 1-1
C27H19ClN6O2S2
GK-jq1cbt 5 (0.121) Cm (2:10)

1: TOF MS ES+
1.11e+007

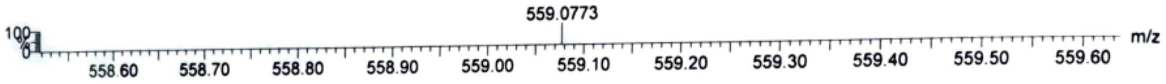

Minimum:				-1.5				
Maximum:		5.0	5.0	50.0				

Mass	Calc. Mass	mDa	PPM	DBE	i-FIT	Norm	Conf(%)	Formula
559.0773	559.0778	-0.5	-0.9	20.5	39.4	n/a	n/a	C27 H20 N6 O2 S2 Cl

JQ1-CBT (X = NH)

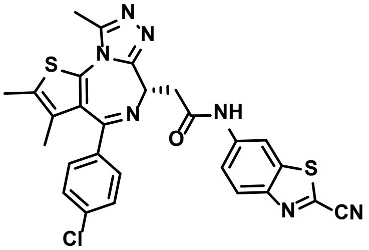

HRMS: *m/z*: $[M+H]^+$ calcd for $C_{27}H_{20}N_7OS_2Cl$: 559.0778; found: 558.0941

Single Mass Analysis

Tolerance = 5.0 PPM / DBE: min = -1.5, max = 50.0
Element prediction: Off
Number of isotope peaks used for i-FIT = 2

Monoisotopic Mass, Even Electron Ions
135 formula(e) evaluated with 1 results within limits (all results (up to 1000) for each mass)
Elements Used:
C: 27-29 H: 0-39 N: 4-7 O: 1-9 S: 0-2 Cl: 1-1
C27H20N7OS2Cl
lsh-jq1-nh-cbt 15 (0.290) Cm (15:23)

1: TOF MS ES+
7.58e+007

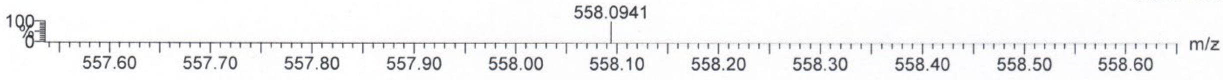

Minimum:				-1.5				
Maximum:		5.0	5.0	50.0				

Mass	Calc. Mass	mDa	PPM	DBE	i-FIT	Norm	Conf(%)	Formula
558.0941	558.0938	0.3	0.5	20.5	43.2	n/a	n/a	C27 H21 N7 O S2 Cl

J252

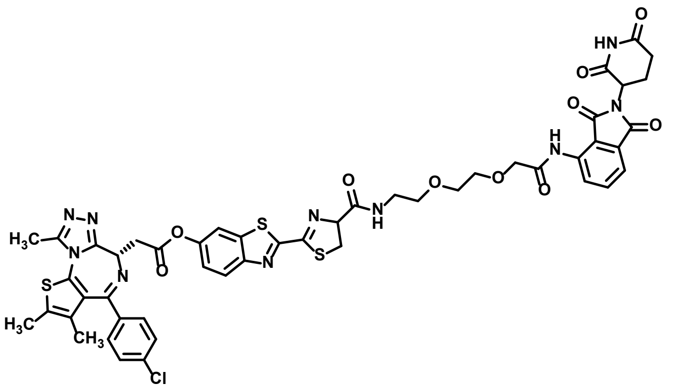

HRMS: *m/z*: $[M+H]^+$ calcd for $C_{49}H_{44}N_{10}O_{10}S_3Cl$: 1063.2093; found: 1063.2057

Elemental Composition Report Page 1

Single Mass Analysis
Tolerance = 10.0 PPM / DBE: min = -1.5, max = 50.0
Element prediction: Off
Number of isotope peaks used for i-FIT = 2

Monoisotopic Mass, Even Electron Ions
462 formula(e) evaluated with 1 results within limits (all results (up to 1000) for each mass)
Elements Used:
C: 0-49 H: 0-44 N: 0-10 O: 0-10 S: 0-3 Cl: 1-1
C49H43ClN10O10S3
GK-j252_2 15 (0.290)

1: TOF MS ES+
7.57e+003

1063.2057

1062.25 1062.50 1062.75 1063.00 1063.25 1063.50 1063.75 1064.00 1064.25 m/z

Minimum:				-1.5				
Maximum:		5.0	10.0	50.0				
Mass	Calc. Mass	mDa	PPM	DBE	i-FIT	Norm	Conf(%)	Formula
1063.2057	1063.2093	-3.6	-3.4	32.5	26.1	n/a	n/a	C49 H44 N10 O10 S3 Cl

J242

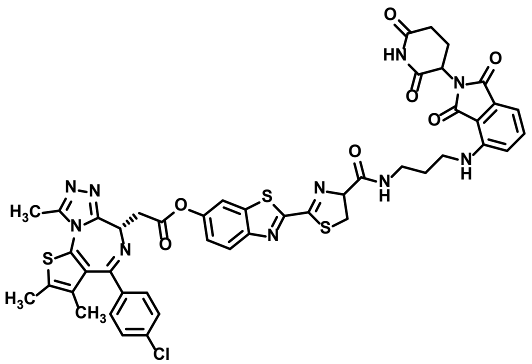

HRMS: *m/z*: $[M+H]^+$ calcd for $C_{46}H_{40}N_{10}O_7S_3Cl$: 975.1932; found: 975.1908

Elemental Composition Report **Page 1**

Single Mass Analysis
Tolerance = 5.0 PPM / DBE: min = -1.5, max = 50.0
Element prediction: Off
Number of isotope peaks used for i-FIT = 2

Monoisotopic Mass, Even Electron Ions
331 formula(e) evaluated with 1 results within limits (all results (up to 1000) for each mass)
Elements Used:
C: 0-46 H: 0-40 N: 0-10 O: 0-7 S: 0-3 Cl: 1-1
C46H39ClN10O7S3
GK-j242 100 (1.788)

1: TOF MS ES+
1.42e+004

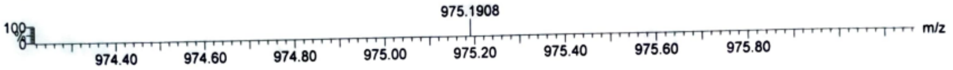

Minimum:				-1.5					
Maximum:		5.0	5.0	50.0					
Mass	Calc. Mass	mDa	PPM	DBE	i-FIT	Norm	Conf(%)	Formula
975.1908	975.1932	-2.4	-2.5	31.5	27.6	n/a	n/a	C46 H40 N10 O7 S3 Cl

T208

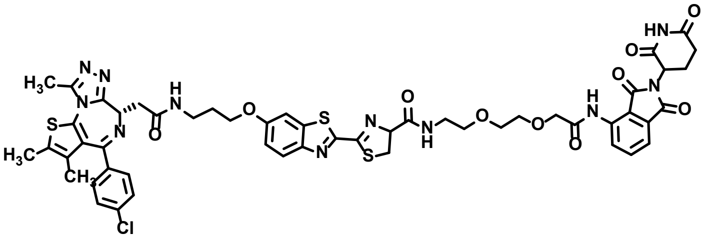

HRMS: *m/z*: $[M+H]^+$ calcd for $C_{52}H_{51}N_{11}O_{10}S_3Cl$: 1120.2671; found: 1120.2678

Elemental Composition Report **Page 1**

Single Mass Analysis
Tolerance = 5.0 PPM / DBE: min = -1.5, max = 50.0
Element prediction: Off
Number of isotope peaks used for i-FIT = 2

Monoisotopic Mass, Even Electron Ions
506 formula(e) evaluated with 1 results within limits (all results (up to 1000) for each mass)
Elements Used:
C: 0-52 H: 0-51 N: 0-11 O: 0-10 S: 0-3 Cl: 1-1
C52H50ClN11O10S3
GK-t208 7 (0.155) Cm (7:15)

1: TOF MS ES+
1.59e+006

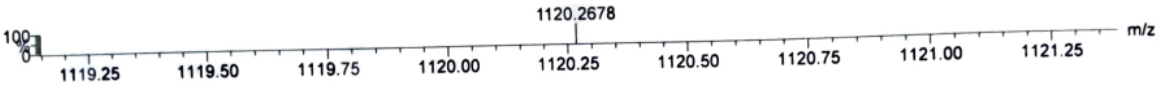

Minimum:				-1.5				
Maximum:		5.0	5.0	50.0				
Mass	Calc. Mass	mDa	PPM	DBE	i-FIT	Norm	Conf(%)	Formula
1120.2678	1120.2671	0.7	0.6	32.5	36.6	n/a	n/a	C52 H51 N11 O10 S3 Cl

^{1}H and ^{13}C NMR spectra

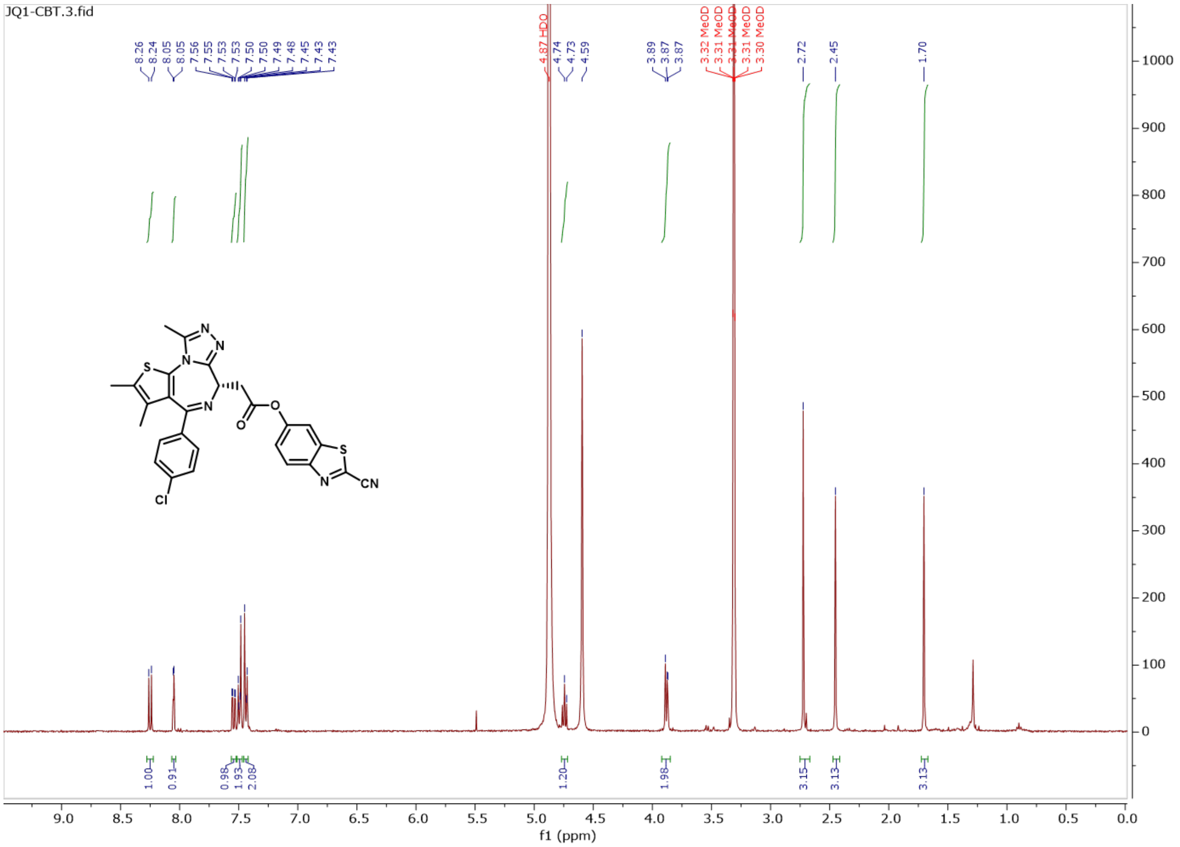

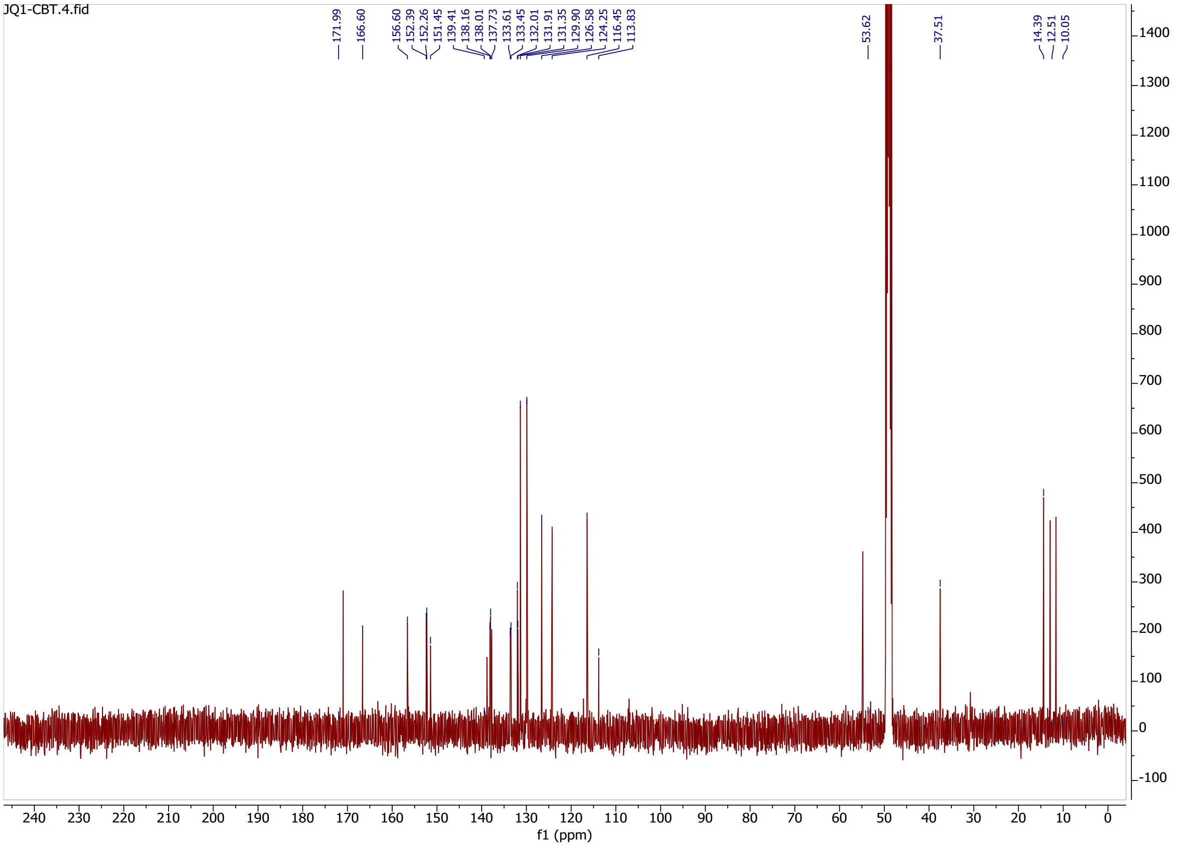

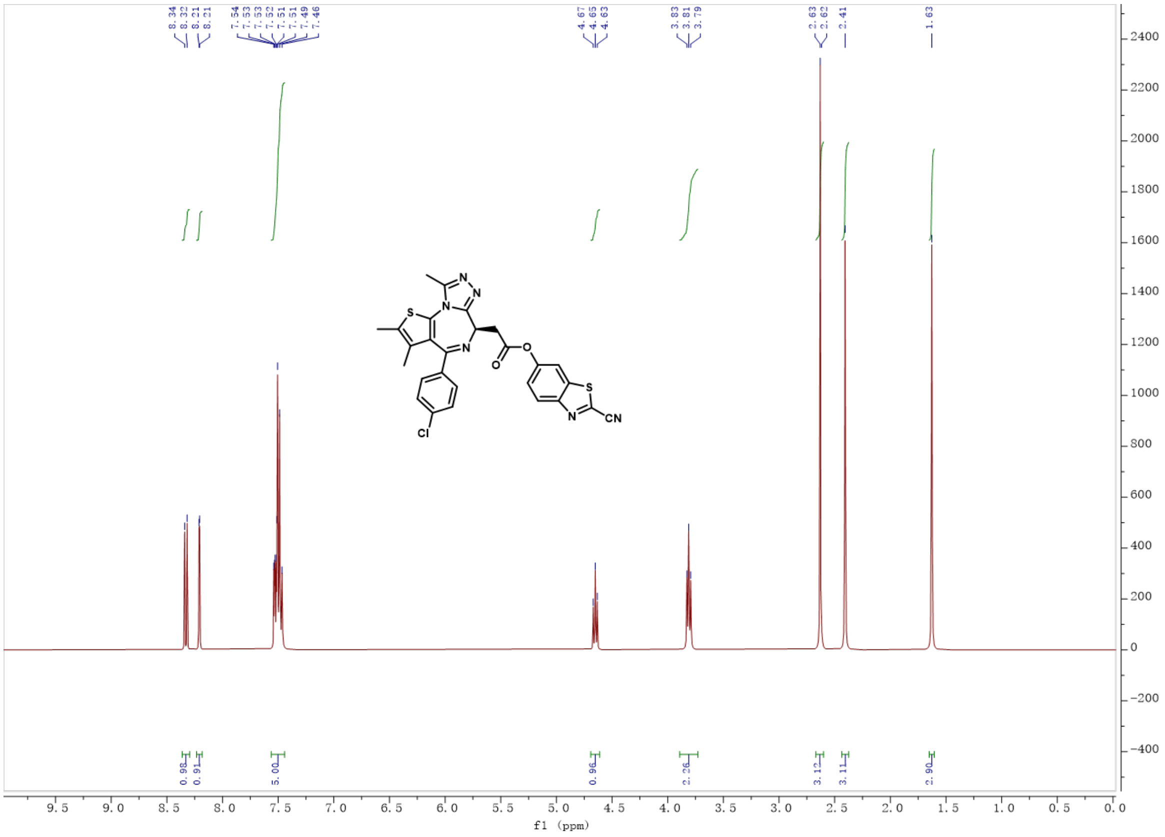

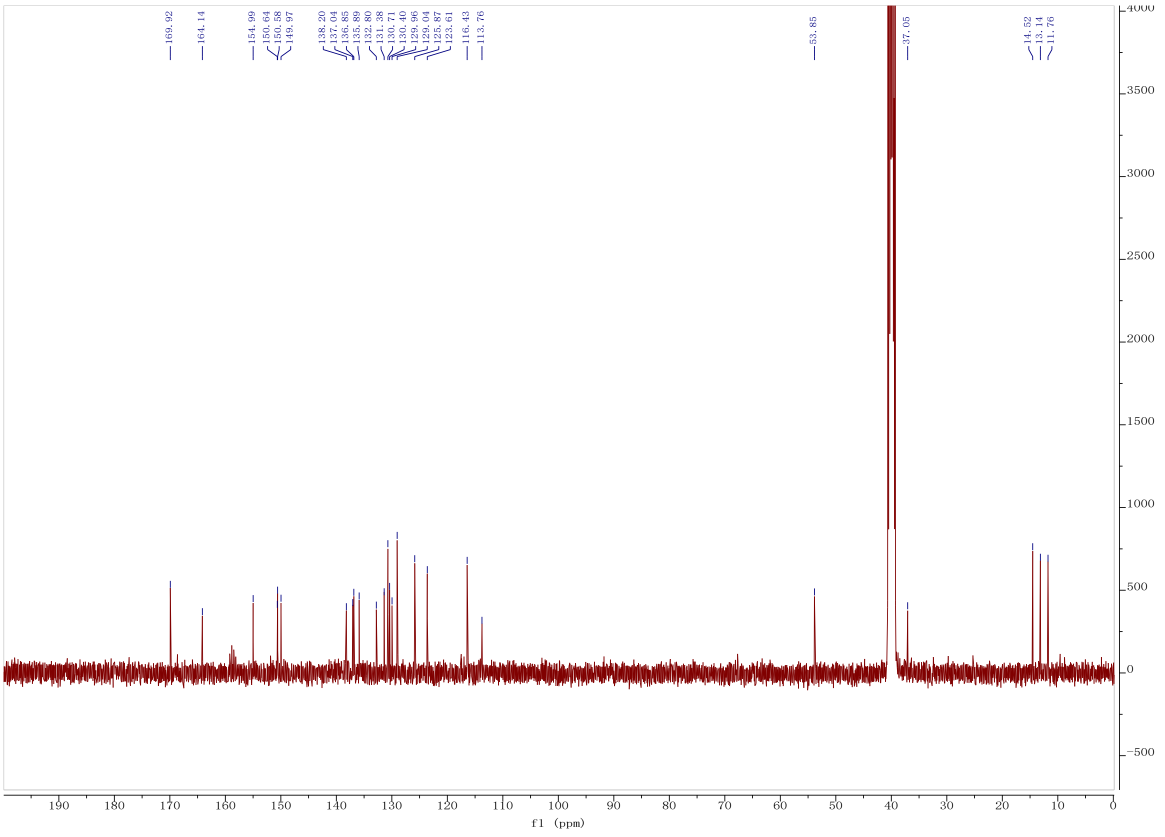

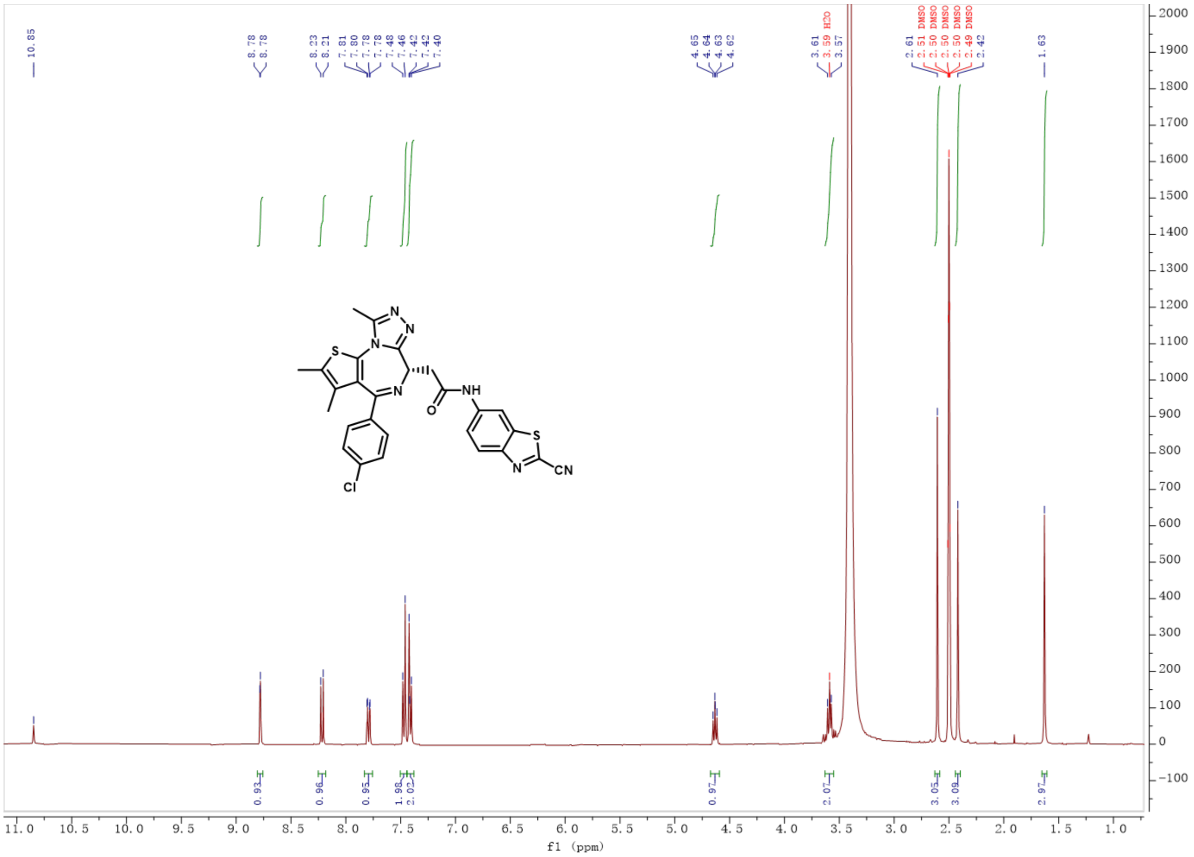
10.85
8.78
8.78
8.23
8.21
7.81
7.80
7.78
7.78
7.48
7.46
7.42
7.42
7.40
4.65
4.64
4.63
4.62
3.61
3.59 H2O
3.57
2.61
2.51 DMSO
2.50 DMSO
2.50 DMSO
2.50 DMSO
2.49 DMSO
2.42
1.63
0.93
0.96
0.95
1.98
2.02
0.97
2.07
3.05
3.09
2.97
S
N
N
N
N
O
NH
S
N
CN
Cl
f1 (ppm)

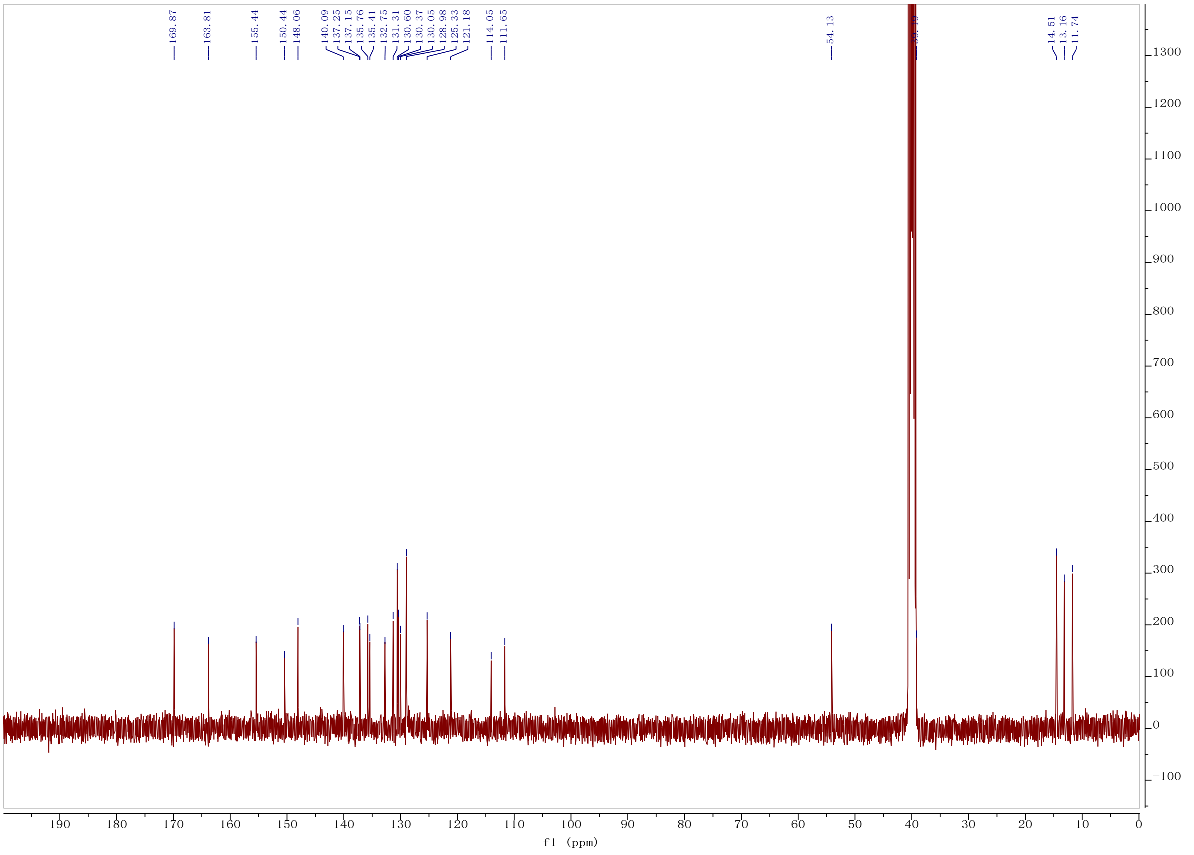
169.87
163.81
155.44
150.44
148.06
140.09
137.25
137.15
135.76
135.41
132.75
131.31
130.60
130.37
130.05
128.98
125.33
121.18
114.05
111.65
54.13
14.51
13.16
11.74
f1 (ppm)

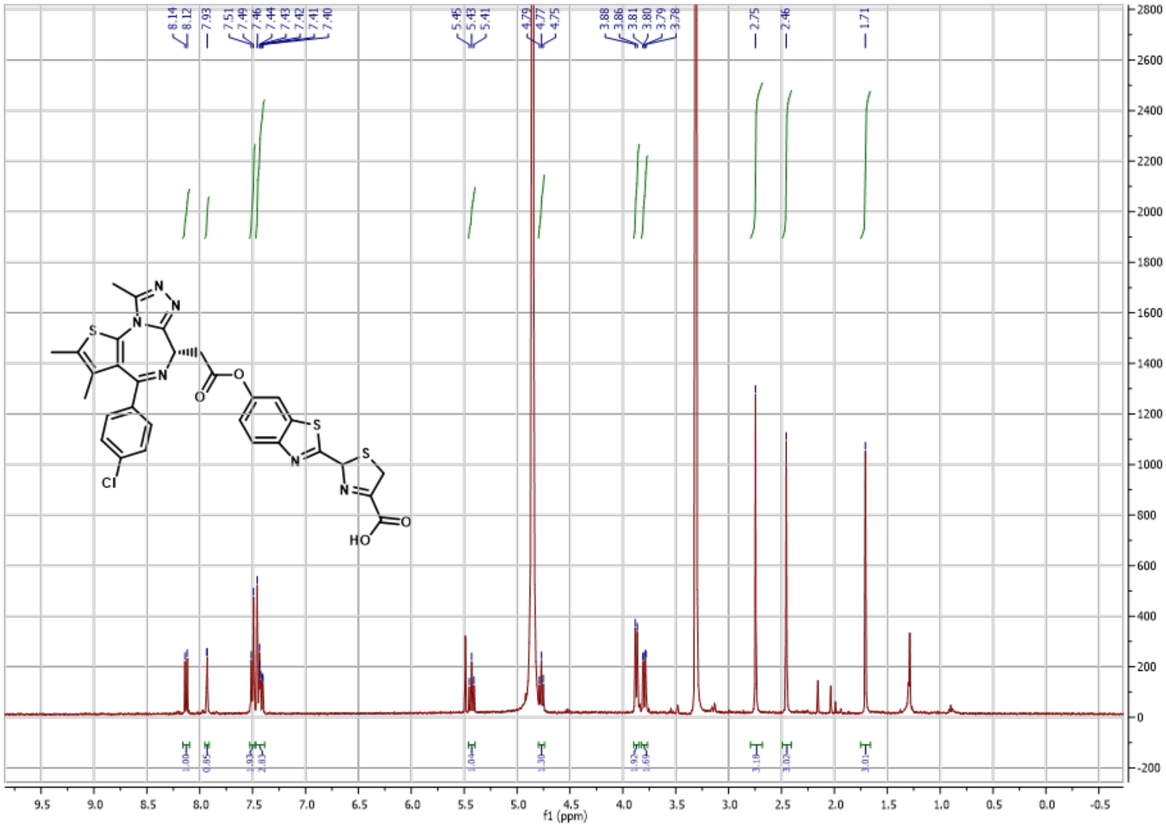

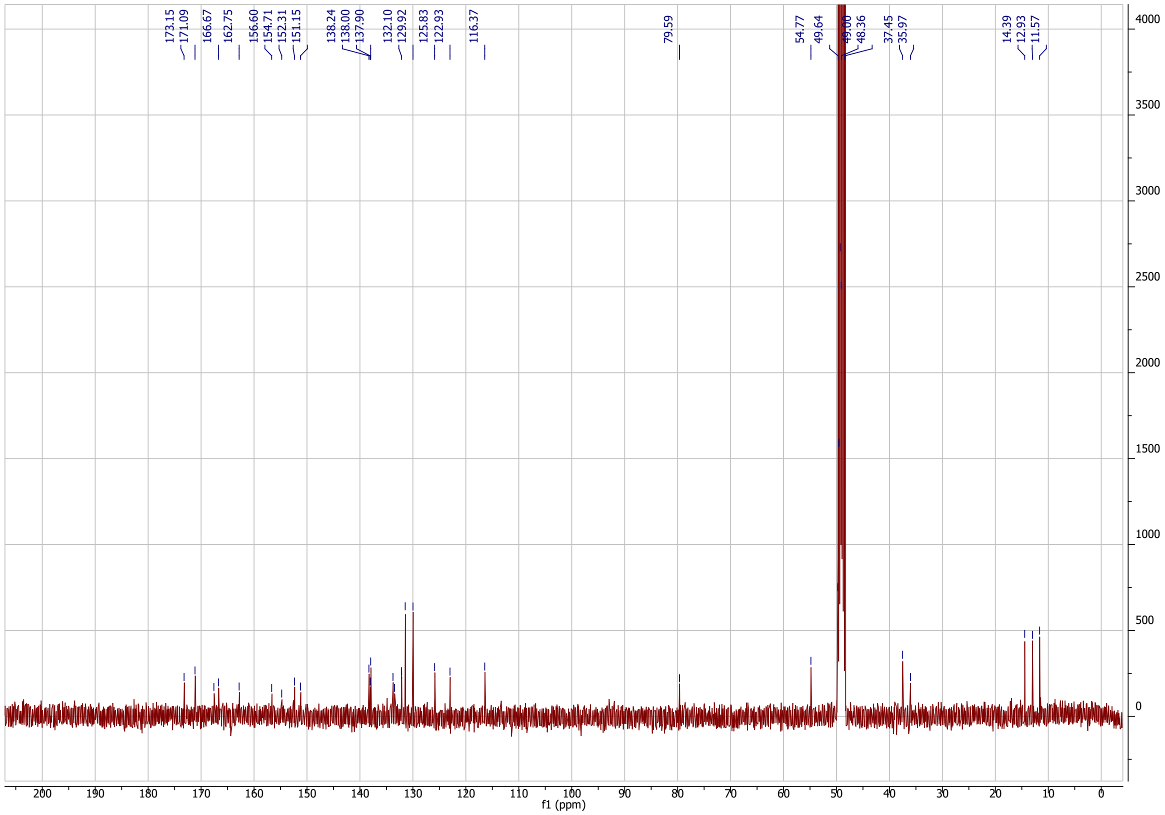

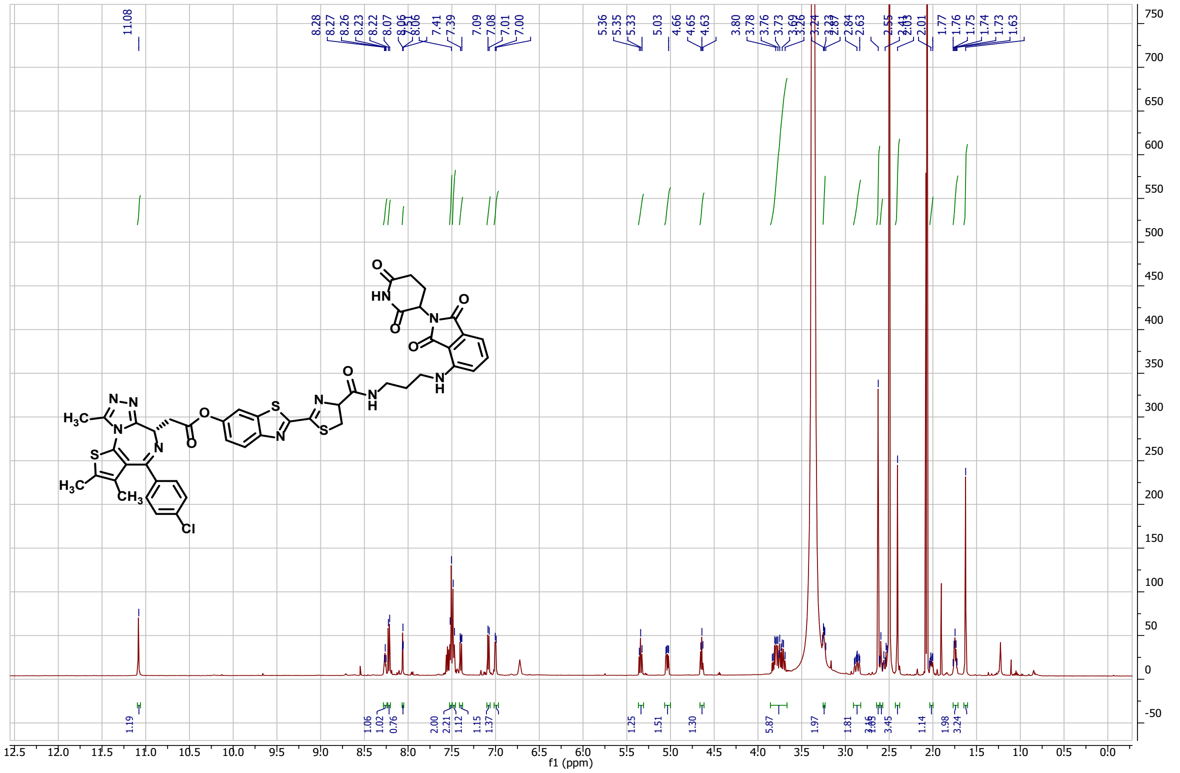
11.08
8.28
8.27
8.26
8.23
8.22
8.07
7.41
7.39
7.09
7.08
7.01
7.00
5.36
5.35
5.33
5.03
4.66
4.65
4.63
3.80
3.78
3.76
3.73
2.84
2.63
2.55
2.01
1.77
1.76
1.75
1.74
1.73
1.63
H3C
N
S
CH3
Cl
O
NH
HN
f1 (ppm)

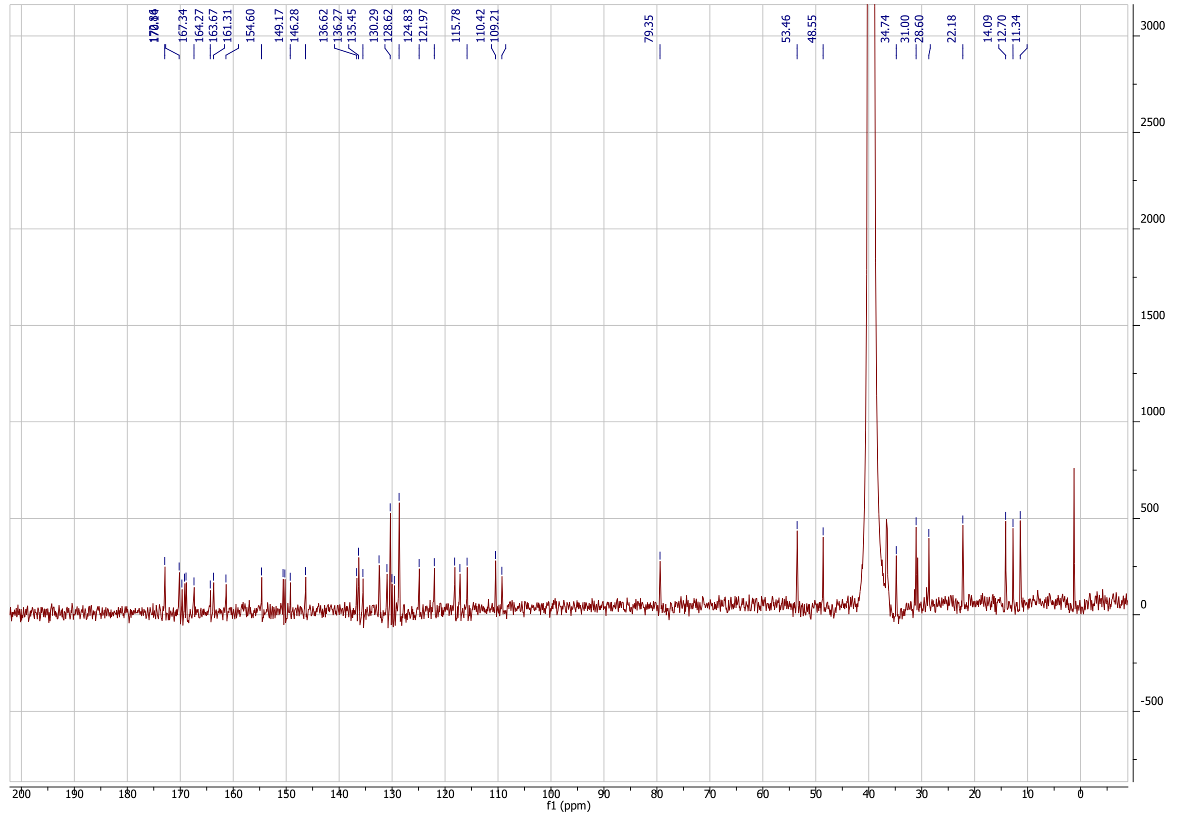
167.34
164.27
163.67
161.31
154.60
149.17
146.28
136.62
136.27
135.45
130.29
128.62
124.83
121.97
115.78
110.42
109.21
79.35
53.46
48.55
34.74
31.00
28.60
22.18
14.09
12.70
11.34
f1 (ppm)

J242 ^{13}CNMR

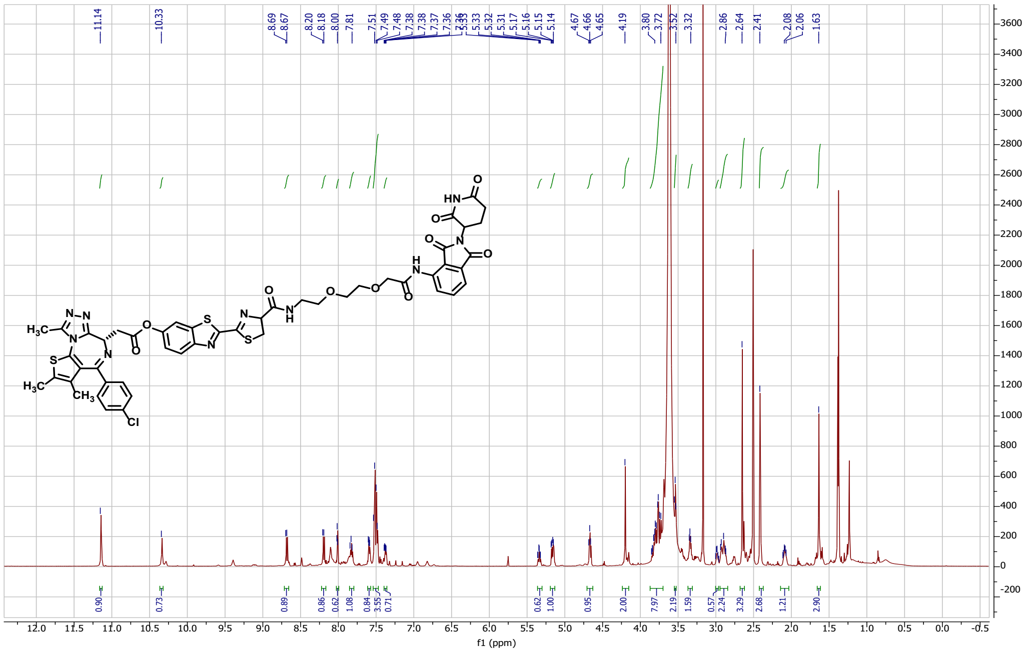

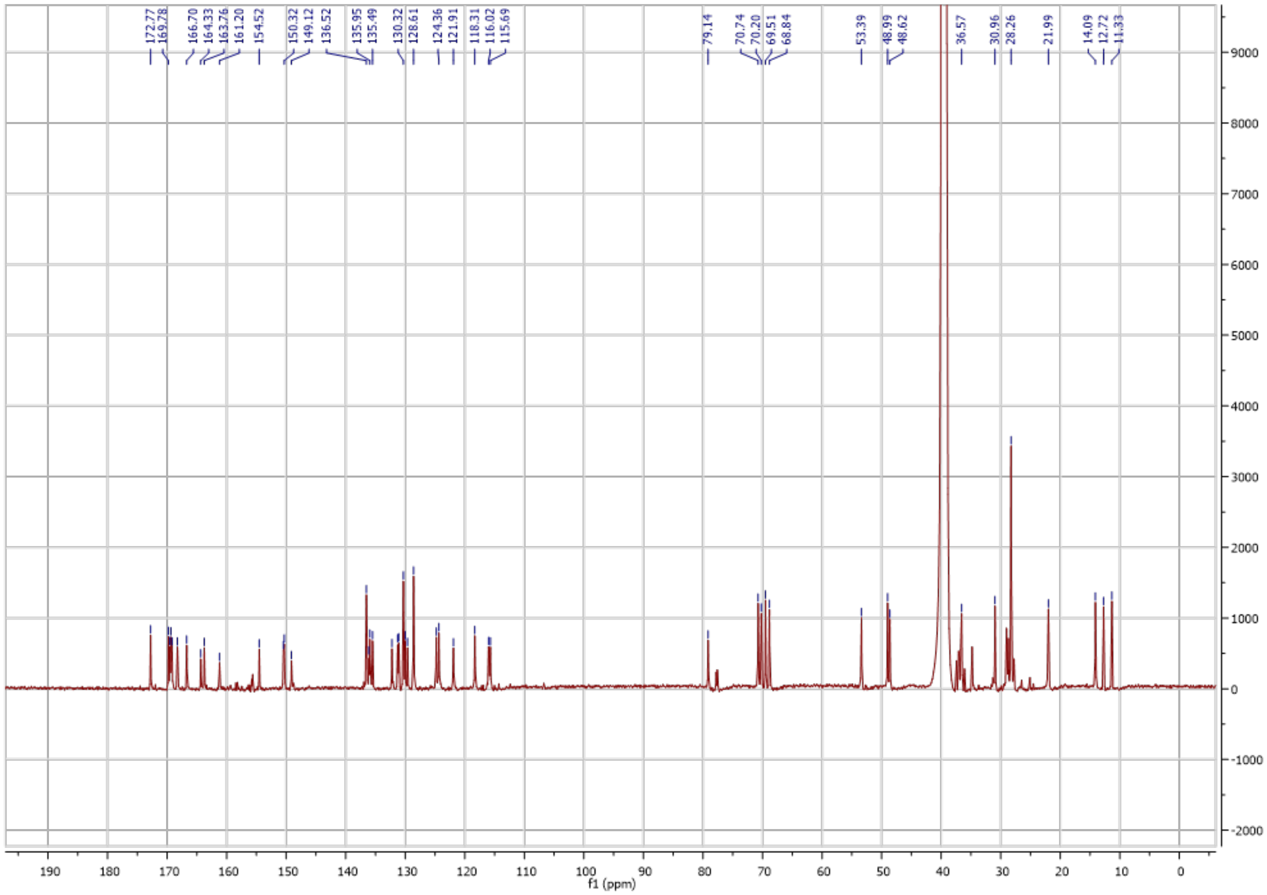

J252 ^{13}C NMR

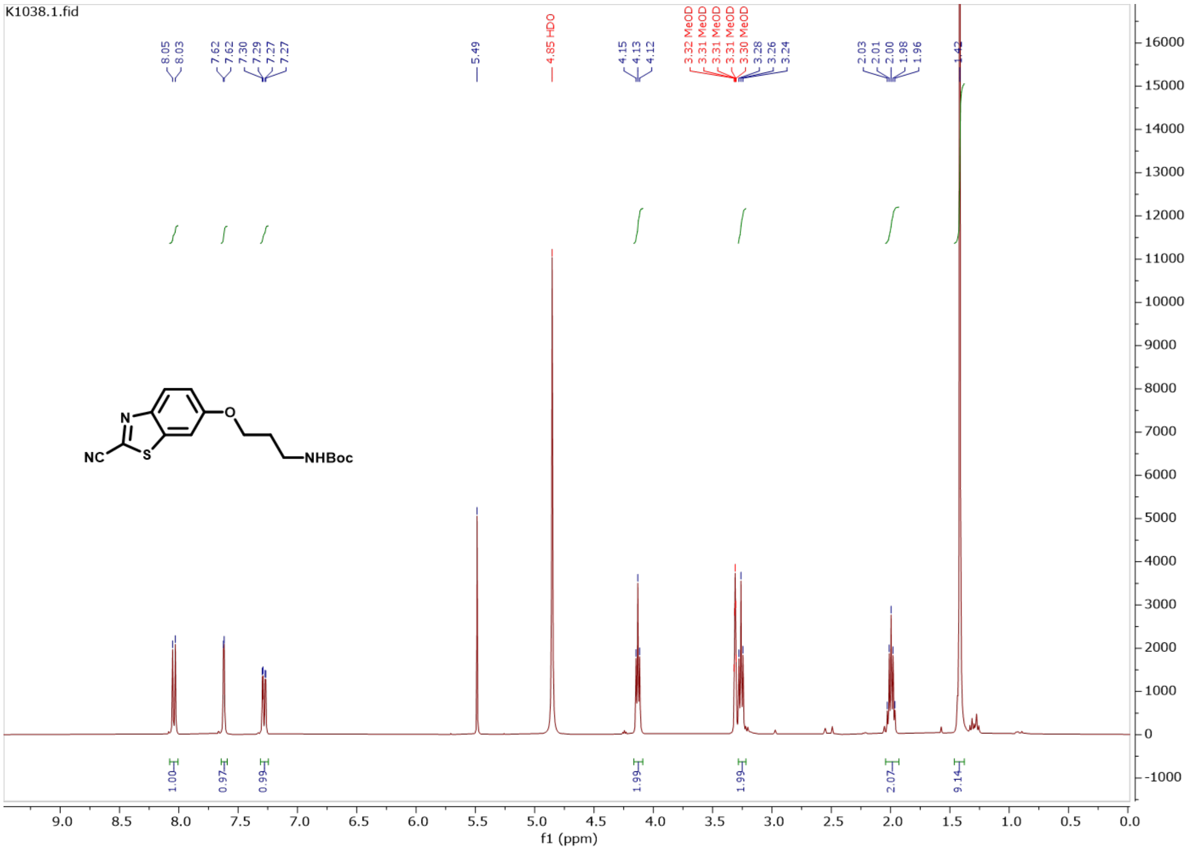
K1038.1.fid
NC
N
S
O
NHBoc
f1 (ppm)

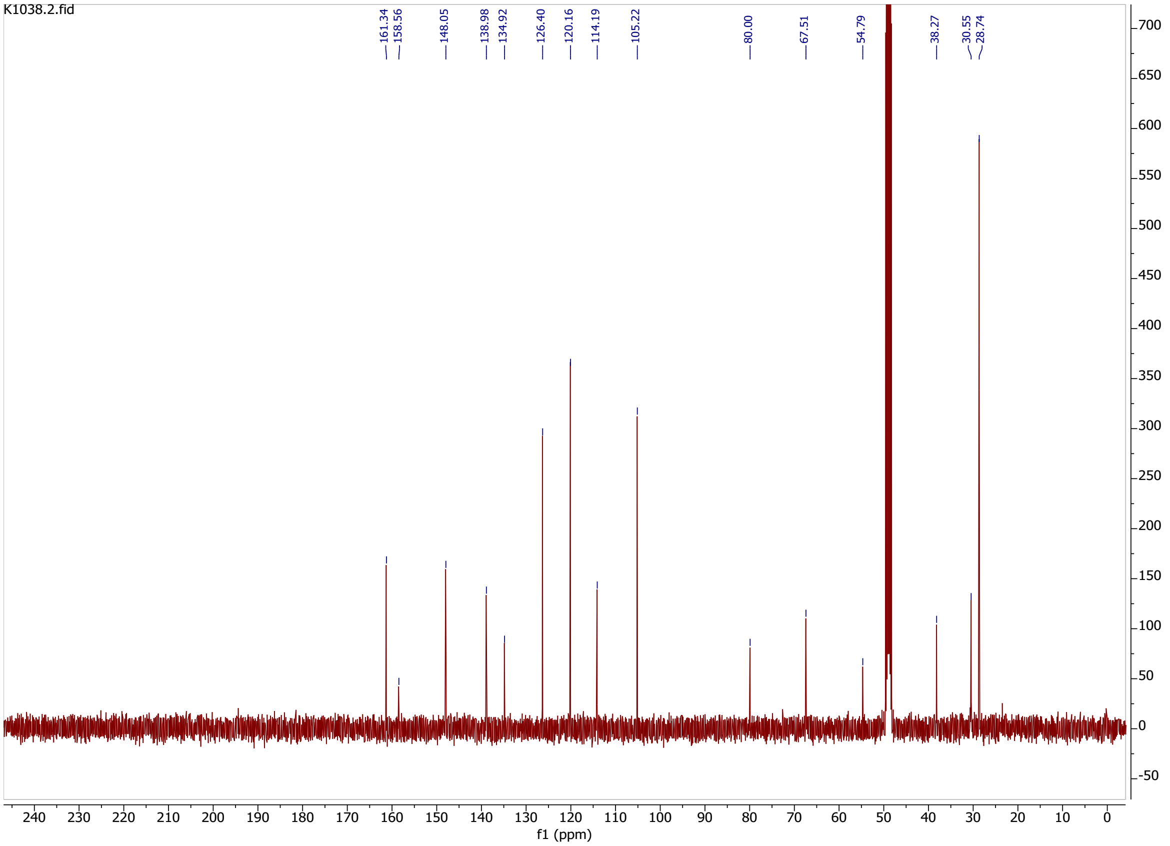
K1038.2.fid
f1 (ppm)

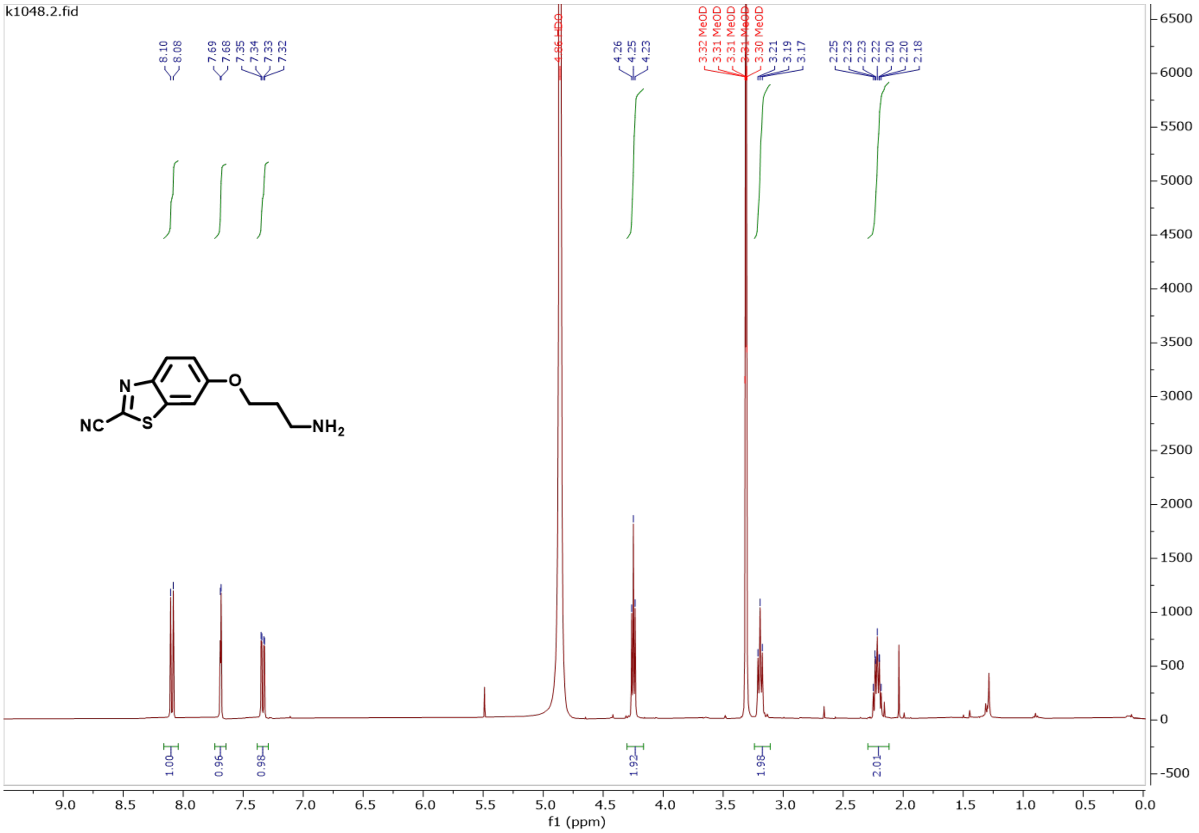
k1048.2.fid
8.10
8.08
7.69
7.68
7.35
7.34
7.33
7.32
4.86 HDO
4.26
4.25
4.23
3.32 MeOD
3.31 MeOD
3.31 MeOD
3.31 MeOD
3.30 MeOD
3.21
3.19
3.17
2.25
2.23
2.23
2.22
2.20
2.20
2.18
N
NC
S
O
NH2
1.00
0.96
0.98
1.92
1.98
2.01
f1 (ppm)

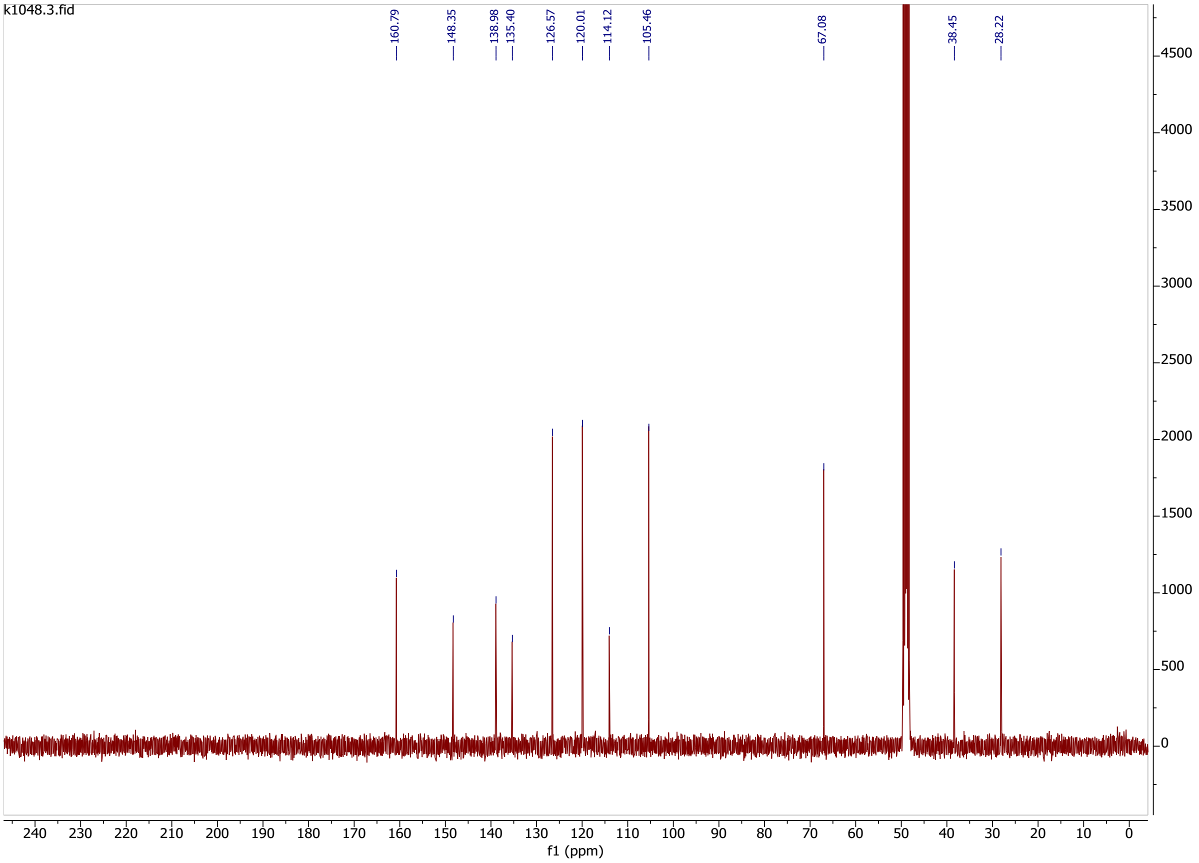
k1048.3.fid
160.79
148.35
138.98
135.40
126.57
120.01
114.12
105.46
67.08
38.45
28.22
f1 (ppm)

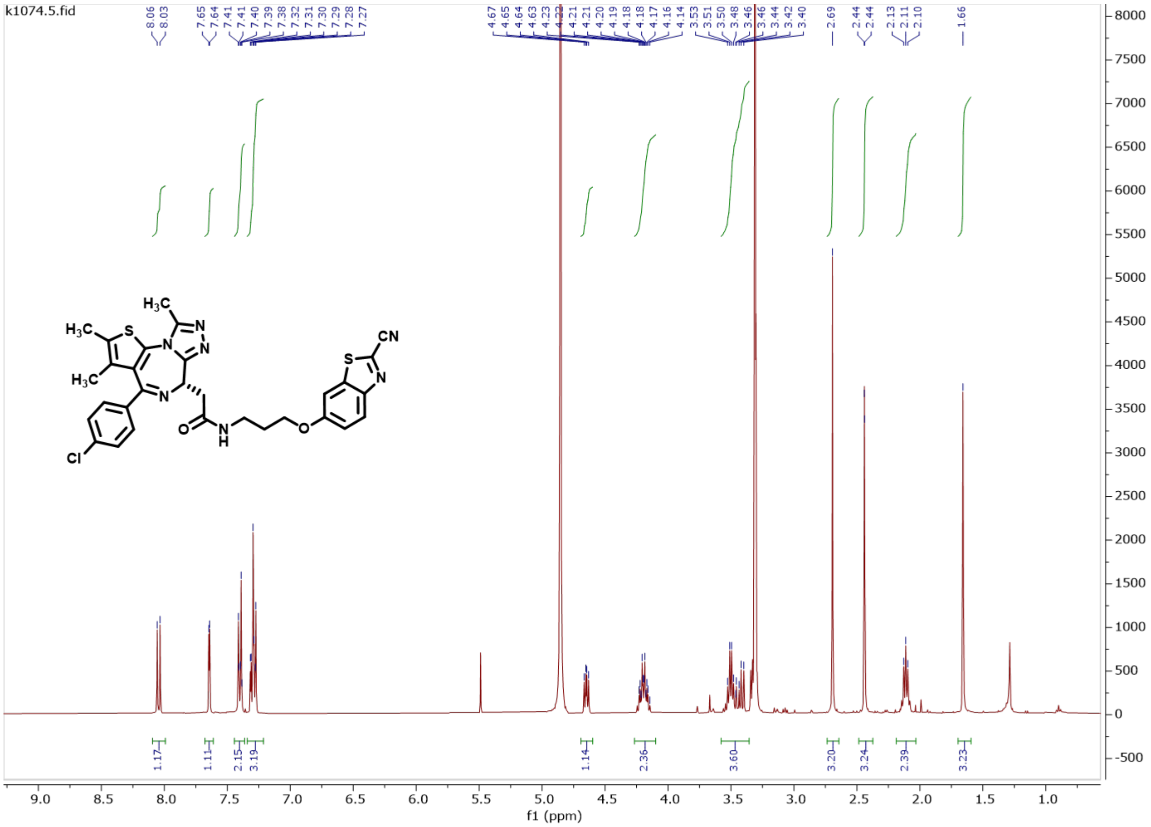
k1074.5.fid
f1 (ppm)

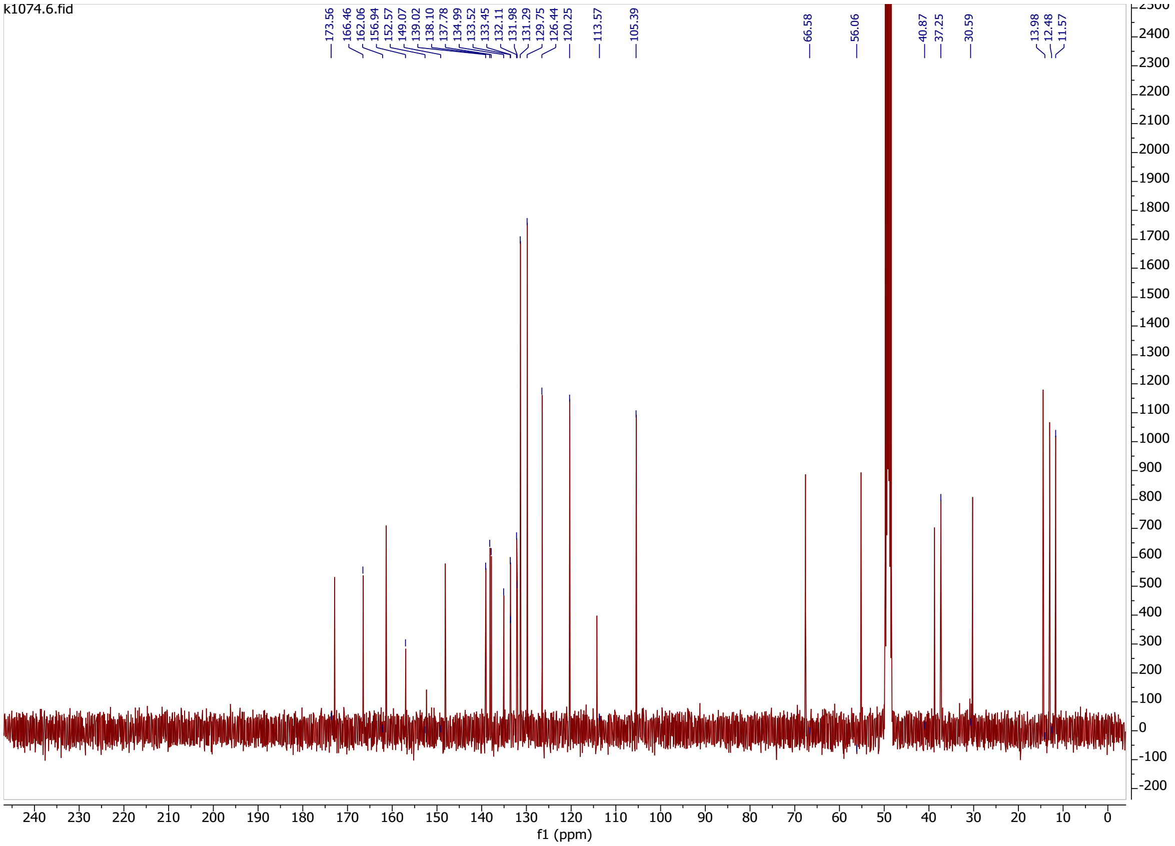
k1074.6.fid
173.56
166.46
162.06
156.94
152.57
149.07
139.02
138.10
137.78
134.99
133.52
133.45
132.11
131.98
131.29
129.75
126.44
120.25
113.57
105.39
66.58
56.06
40.87
37.25
30.59
13.98
12.48
11.57
f1 (ppm)

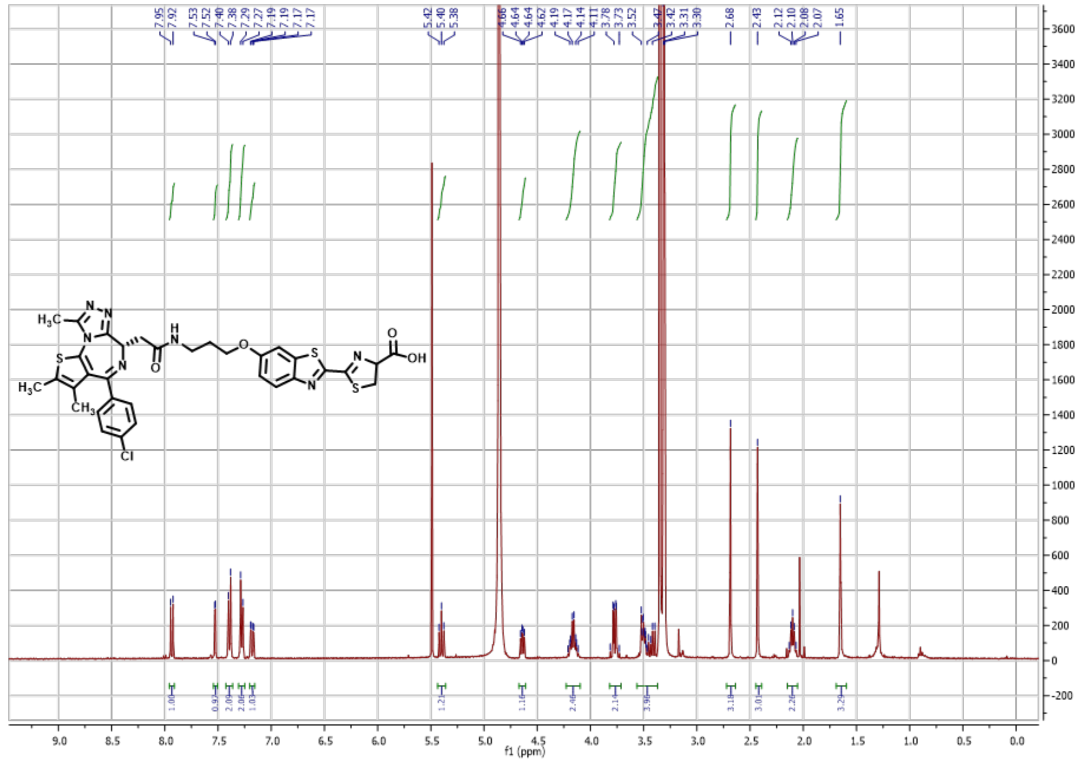

^{1}H NMR of JQ1-C3-CBT-Cys

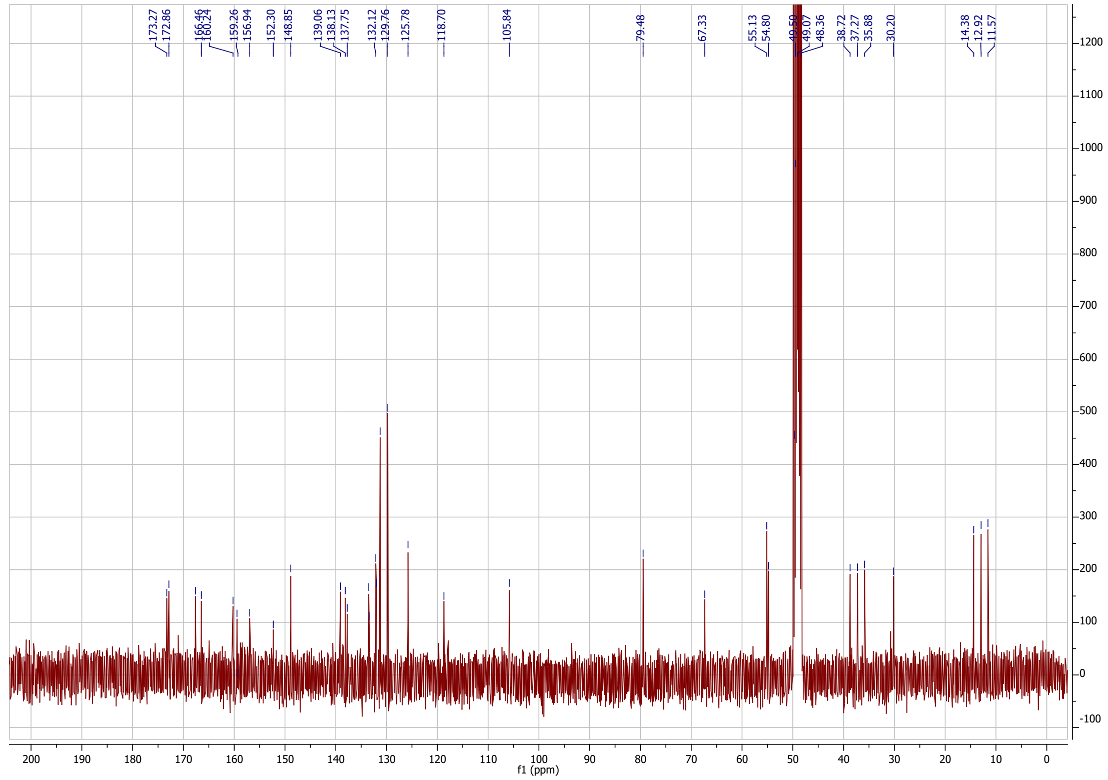

^{13}C NMR of JQ1-C3-CBT-Cys

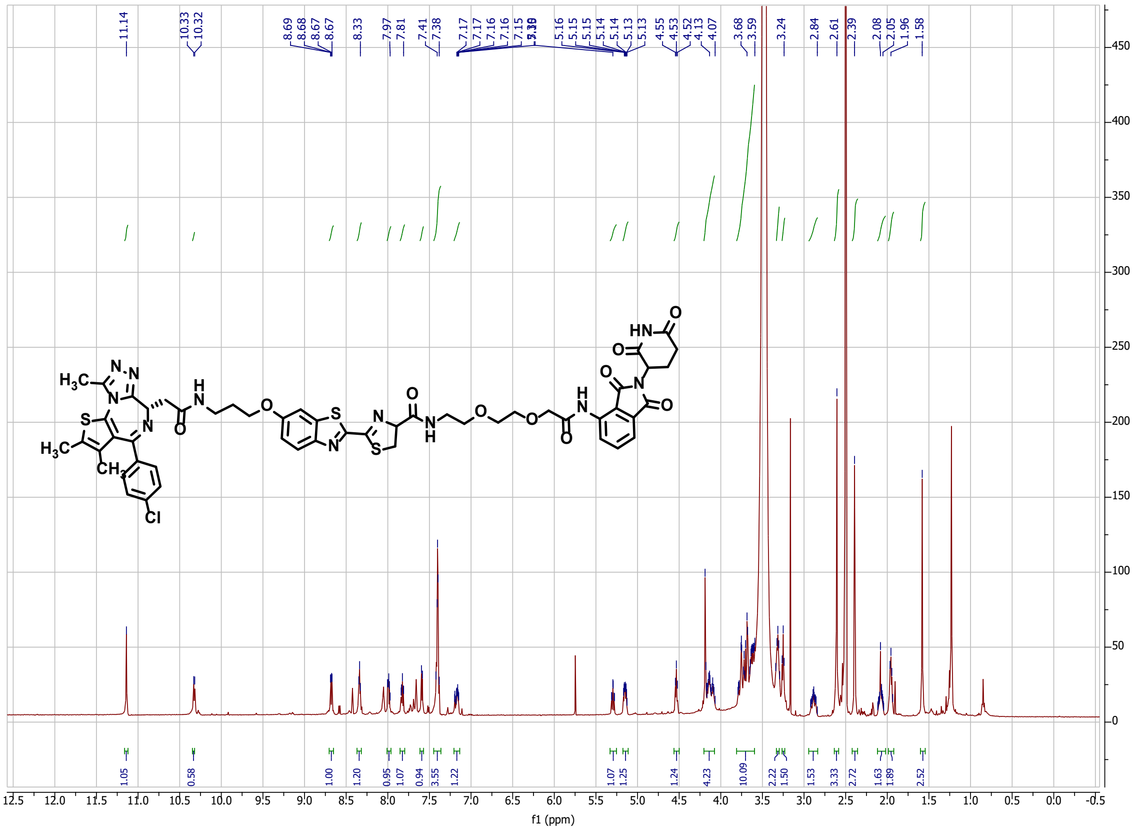

^{1}H NMR of T208

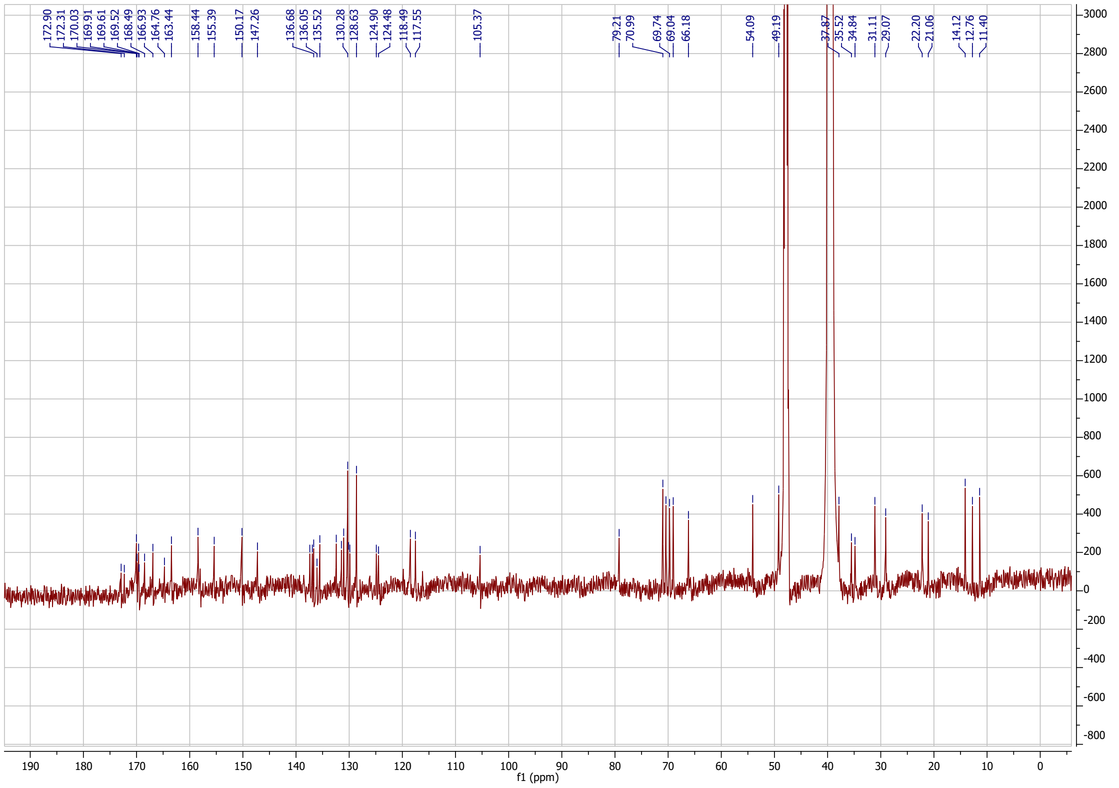

^{13}C NMR of T208

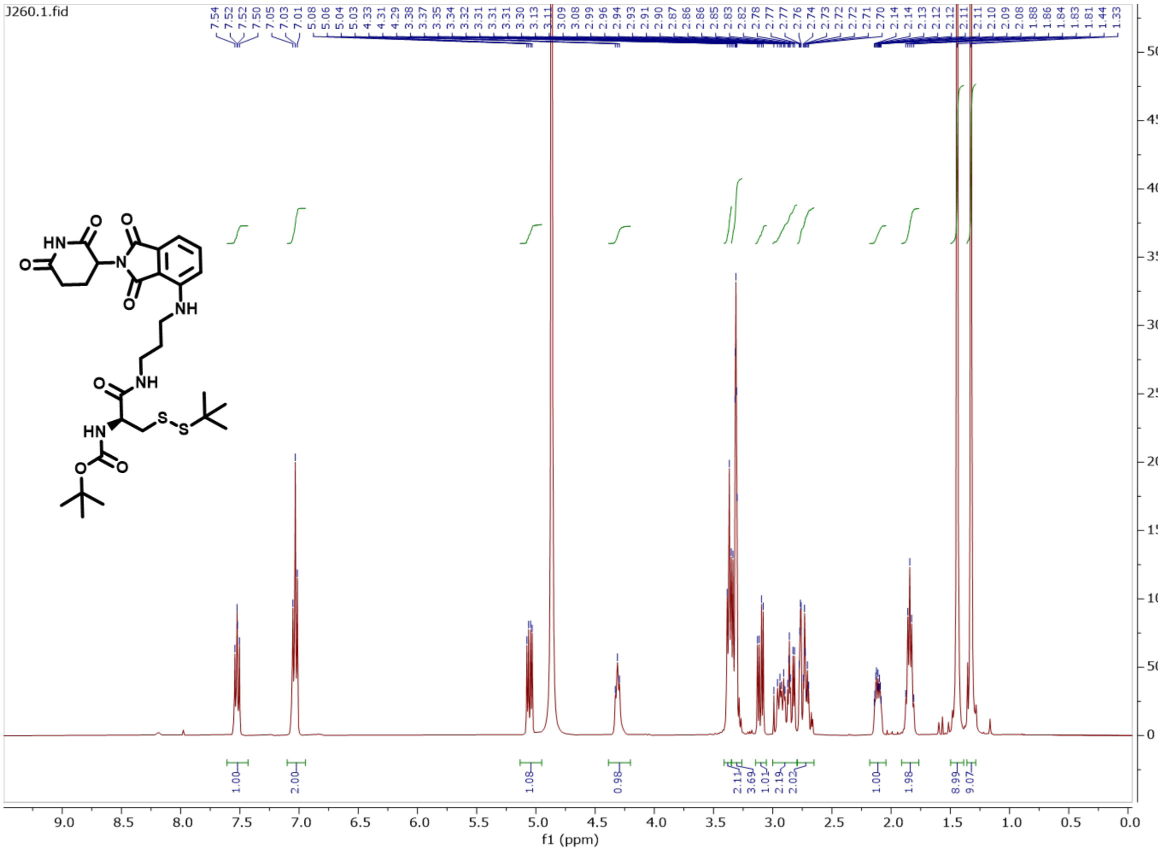
J260.1.fid
7.54 7.52 7.52 7.50 7.05 7.03 7.01 5.08 5.06 5.04 5.03 4.33 4.31 4.29 3.38 3.37 3.35 3.34 3.32 3.31 3.31 3.31 3.30 3.13 3.11 3.09 3.08 2.99 2.96 2.94 2.93 2.91 2.90 2.87 2.86 2.85 2.83 2.82 2.78 2.77 2.77 2.76 2.74 2.73 2.72 2.72 2.71 2.70 2.14 2.14 2.13 2.12 2.12 2.11 2.11 2.10 2.09 2.08 1.88 1.86 1.84 1.83 1.81 1.44 1.33
HN
O
N
NH
S
1.00 2.00 1.08 0.98 2.11 3.69 1.01 2.19 2.02 1.00 1.98 8.99 9.07
f1 (ppm)

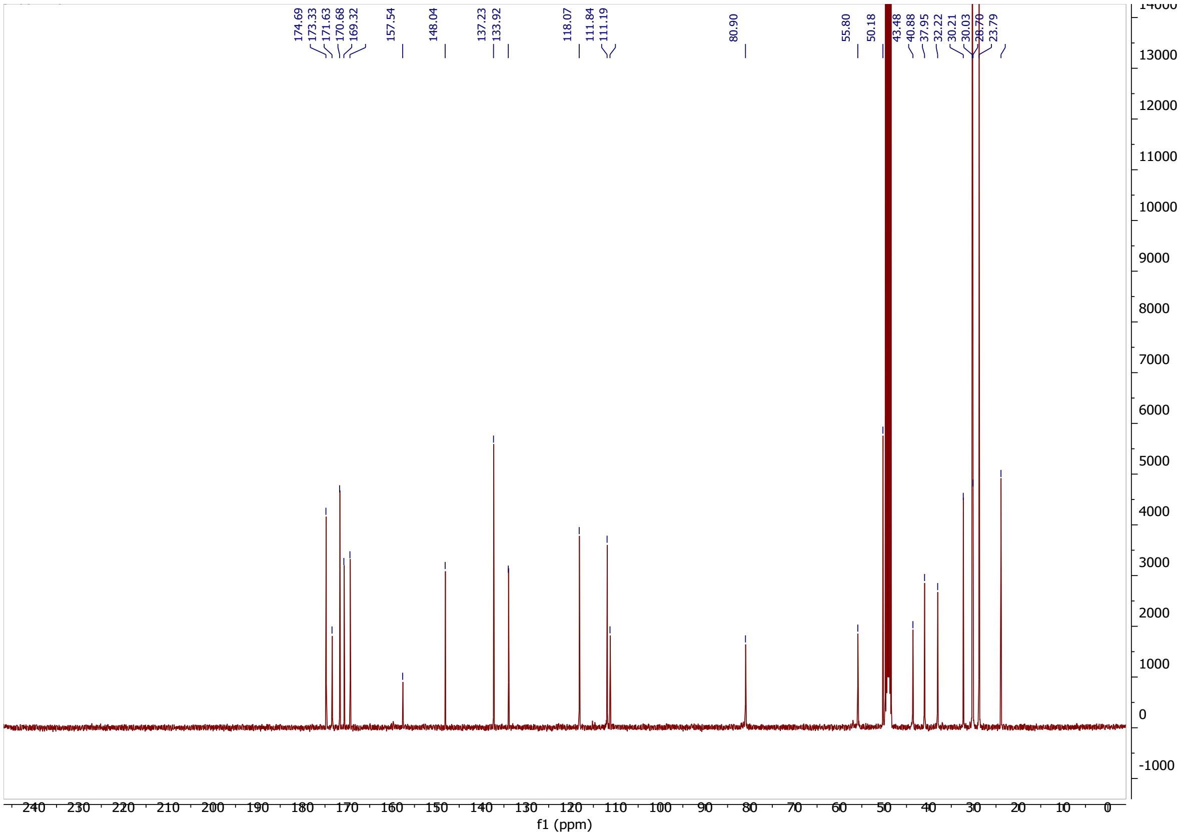
174.69 173.33 171.63 170.68 169.32 157.54 148.04 137.23 133.92 118.07 111.84 111.19 80.90 55.80 50.18 43.48 40.88 37.95 32.22 30.21 30.03 28.70 23.79
f1 (ppm)

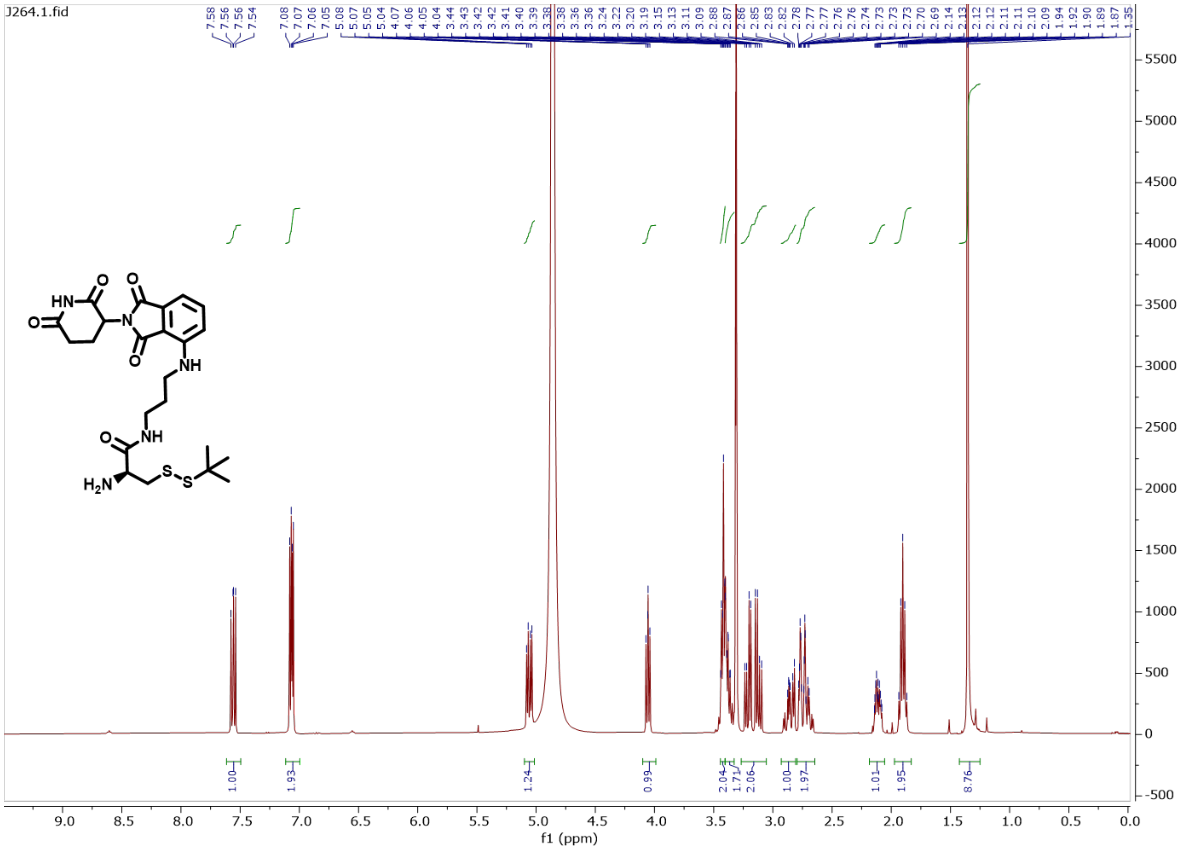
J264.1.fid
7.58 7.56 7.56 7.54 7.08 7.07 7.06 7.05 5.08 5.07 5.05 5.04 4.07 4.06 4.05 4.04 3.44 3.43 3.42 3.41 3.40 3.39 3.38 3.38 3.36 3.36 3.24 3.22 3.20 3.19 3.15 3.13 3.11 3.09 2.88 2.87 2.86 2.85 2.83 2.82 2.78 2.77 2.77 2.76 2.74 2.73 2.73 2.73 2.70 2.69 2.14 2.13 2.12 2.12 2.11 2.11 2.10 2.09 1.94 1.92 1.90 1.89 1.87 1.35
1.00 1.93 1.24 0.99 2.04 1.71 2.06 1.00 1.97 1.01 1.95 8.76
HN
O
N
NH
H_2N
S
f1 (ppm)

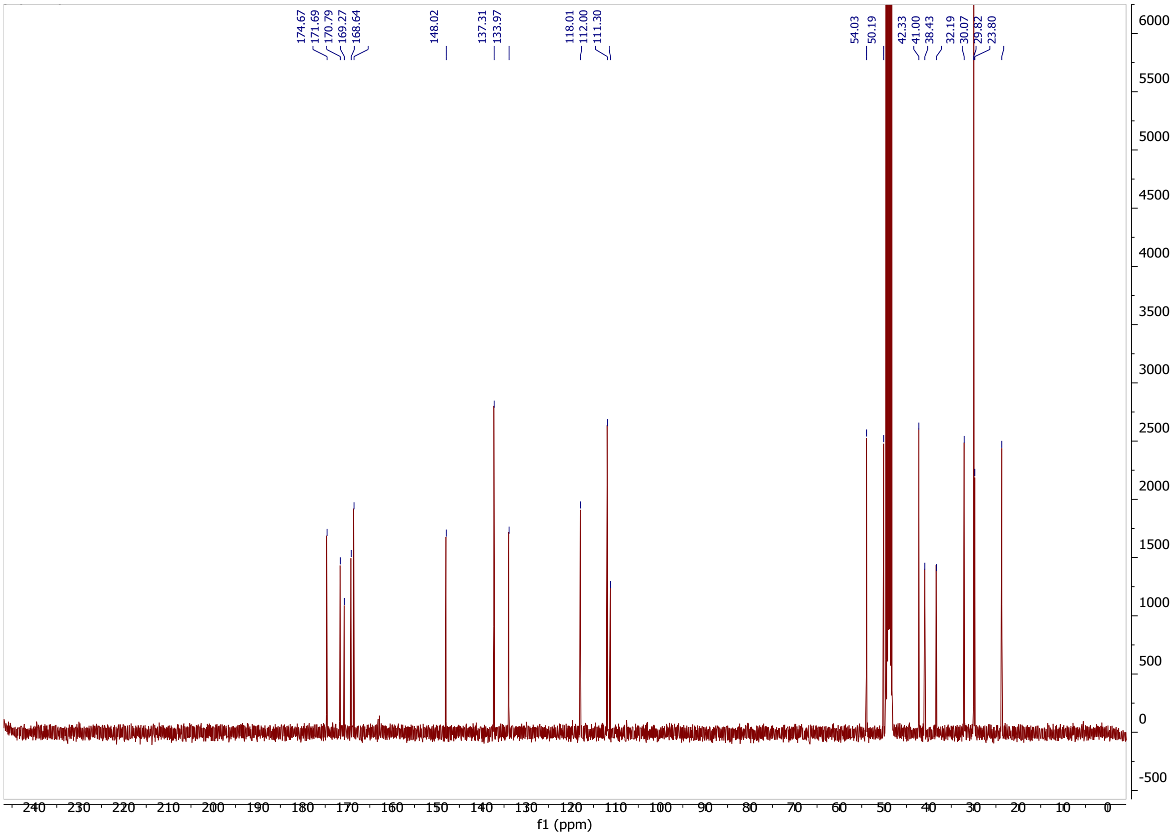
174.67 171.69 170.79 169.27 168.64 148.02 137.31 133.97 118.01 112.00 111.30 54.03 50.19 42.33 41.00 38.43 32.19 30.07 29.82 23.80
f1 (ppm)

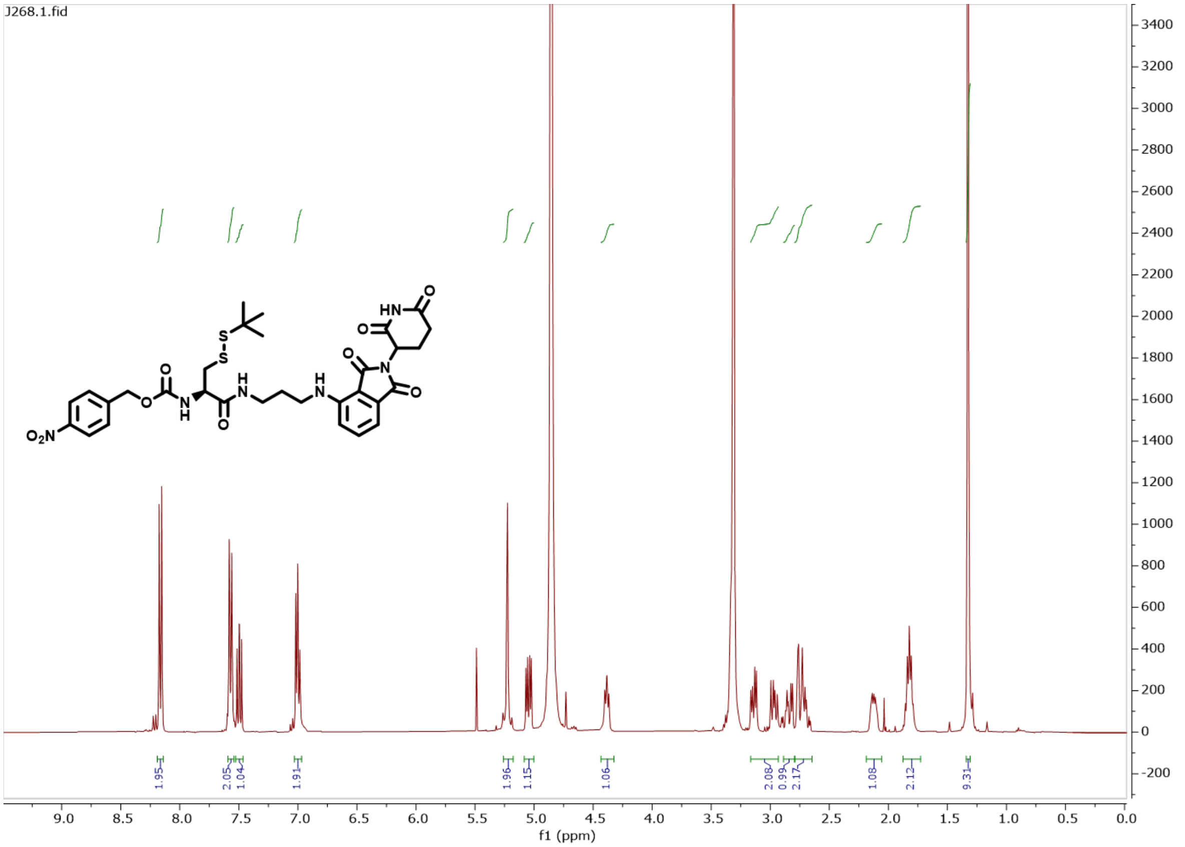
J268.1.fid
1.95
2.05
1.04
1.91
1.96
1.15
1.06
2.08
0.99
2.17
1.08
2.12
9.31
f1 (ppm)

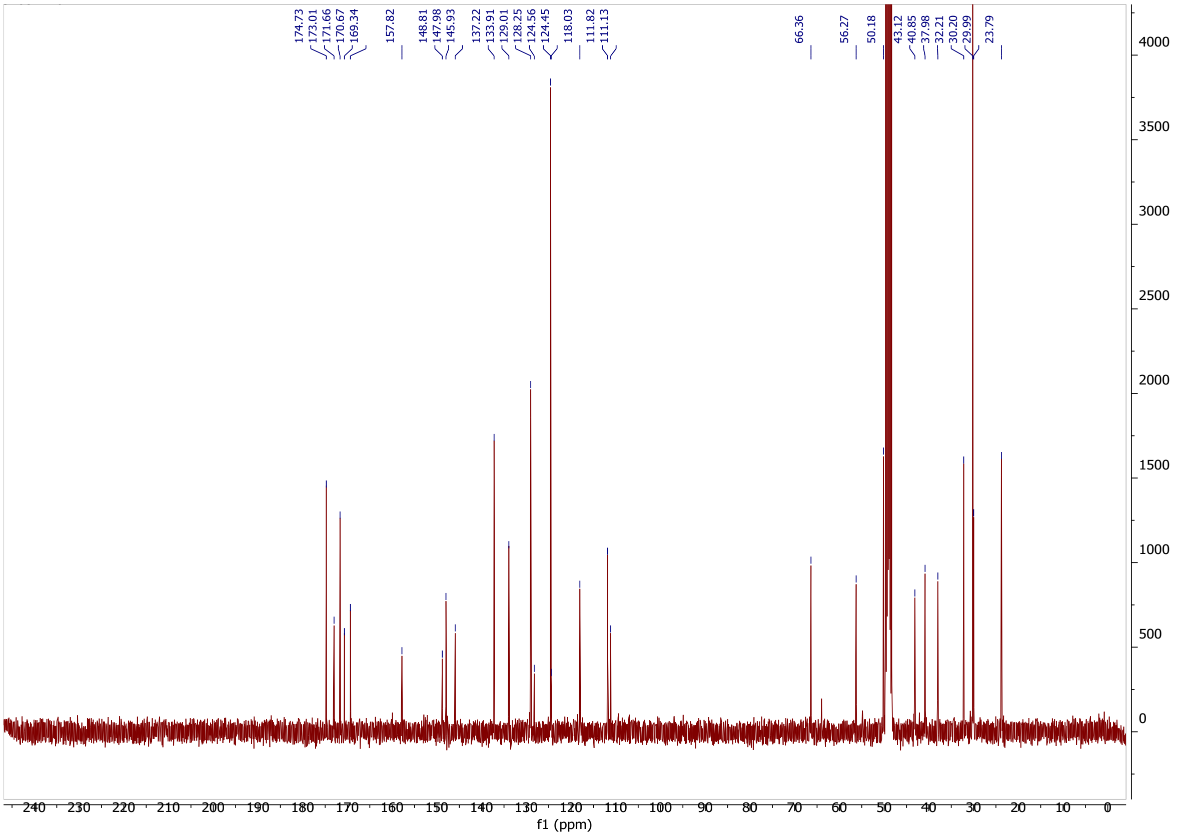
174.73
173.01
171.66
170.67
169.34
157.82
148.81
147.98
145.93
137.22
133.91
129.01
128.25
124.56
124.45
118.03
111.82
111.13
66.36
56.27
50.18
43.12
40.85
37.98
32.21
30.20
29.99
23.79
f1 (ppm)

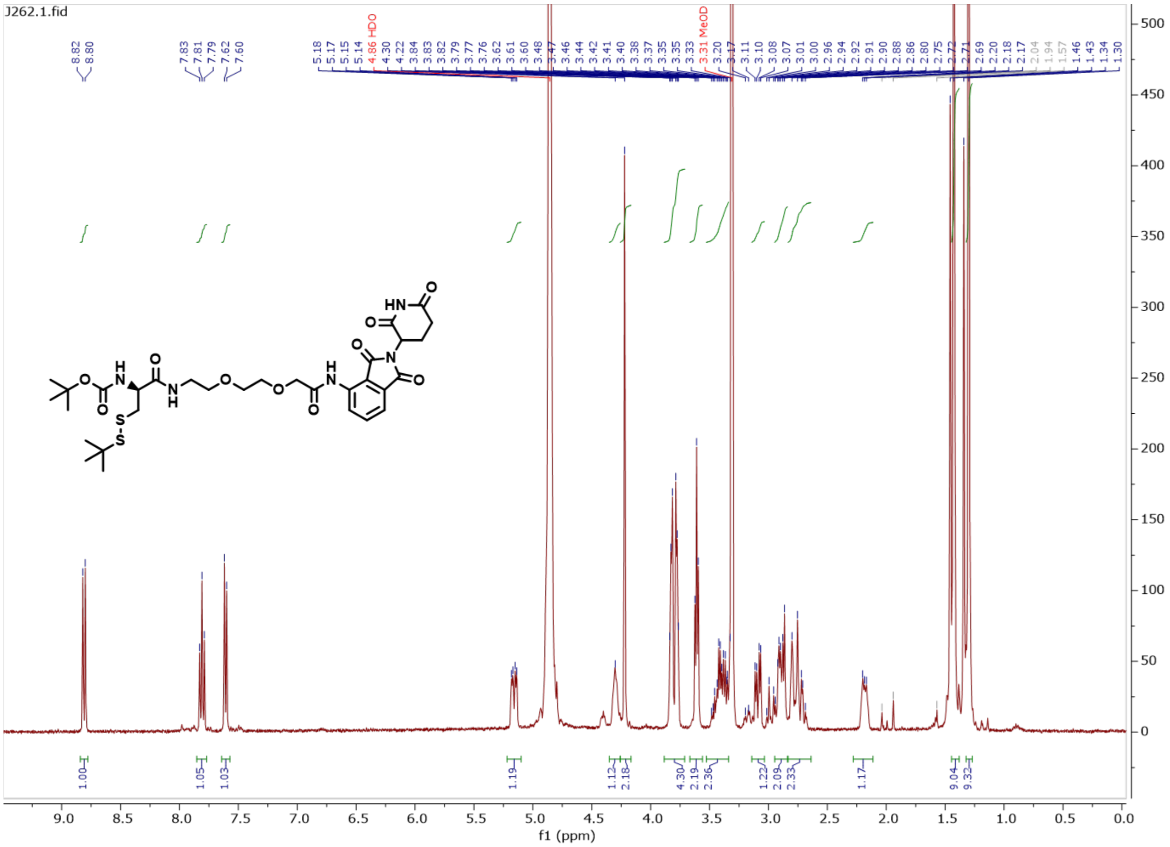
J262.1.fid
8.82 8.80
7.83 7.81 7.79 7.62 7.60
5.18 5.17 5.15 5.14 4.86 HDO 4.30 4.22 3.84 3.83 3.82 3.79 3.77 3.76 3.62 3.61 3.60 3.48 3.47 3.46 3.44 3.42 3.41 3.40 3.38 3.37 3.35 3.35 3.33 3.31 MeOD 3.20 3.17 3.11 3.10 3.08 3.07 3.01 3.00 2.96 2.94 2.92 2.91 2.90 2.88 2.86 2.80 2.75 2.72 2.71 2.69 2.20 2.18 2.17 2.04 1.94 1.57 1.46 1.43 1.34 1.30
HN
O
H
N
O
N
H
O
O
H
N
S
S
1.00 1.05 1.03 1.19 1.12 2.18 4.30 2.19 2.36 1.22 2.09 2.33 1.17 9.04 9.32
f1 (ppm)
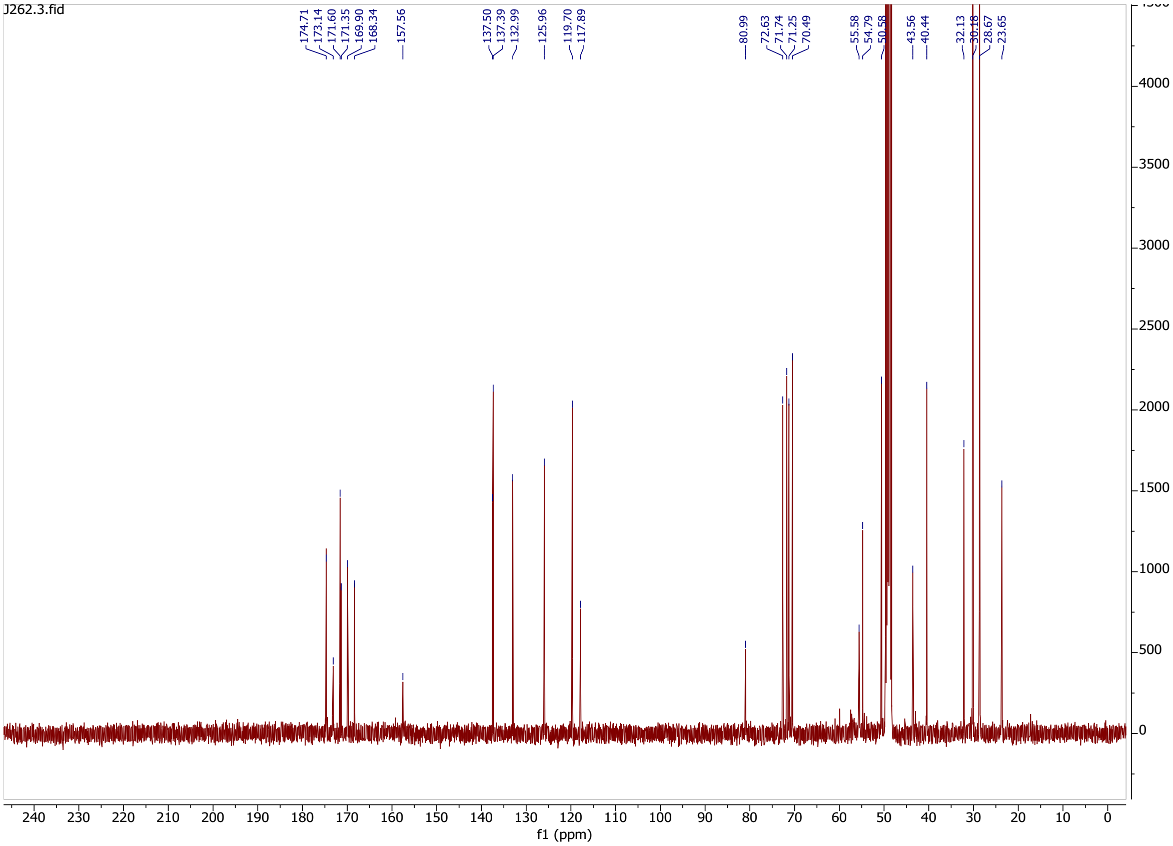
J262.3.fid
174.71 173.14 171.60 171.35 169.90 168.34 157.56
137.50 137.39 132.99 125.96 119.70 117.89
80.99 72.63 71.74 71.25 70.49
55.58 54.79 50.58 43.56 40.44 32.13 30.18 28.67 23.65
f1 (ppm)

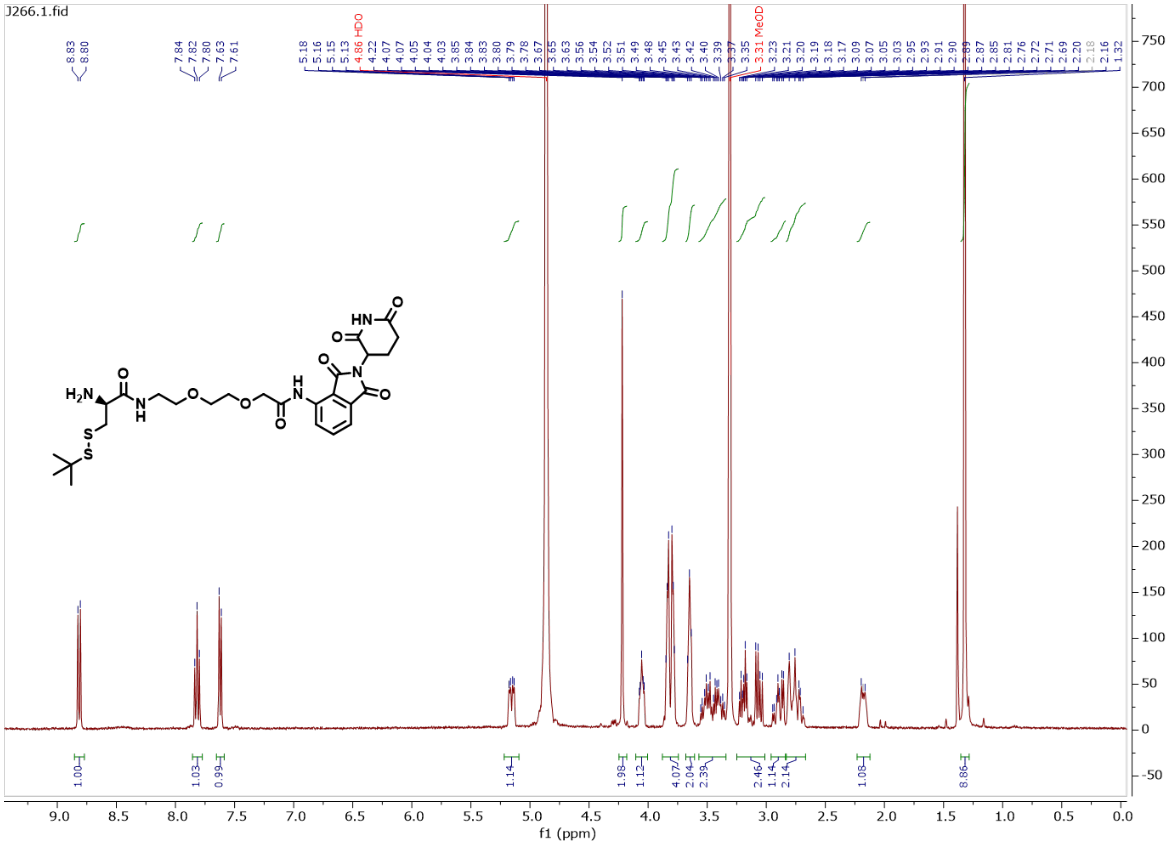
J266.1.fid
f1 (ppm)

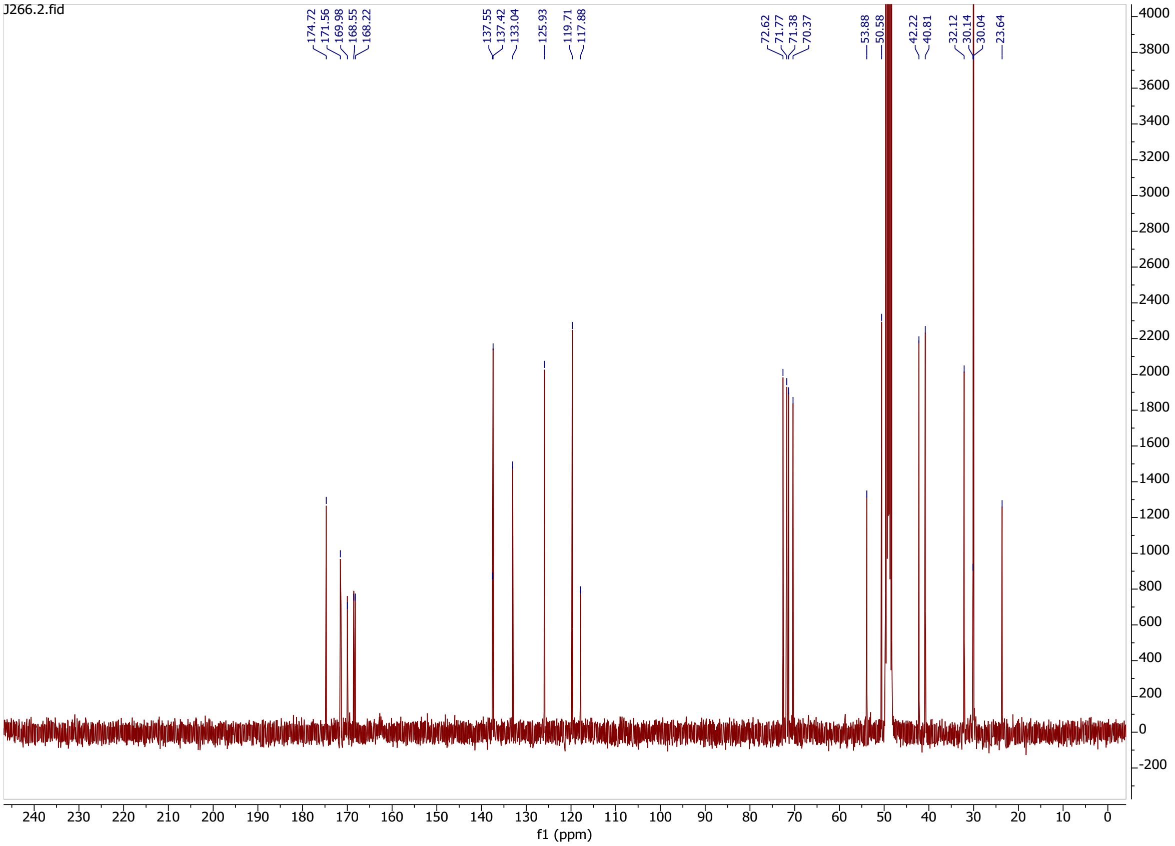
J266.2.fid
f1 (ppm)

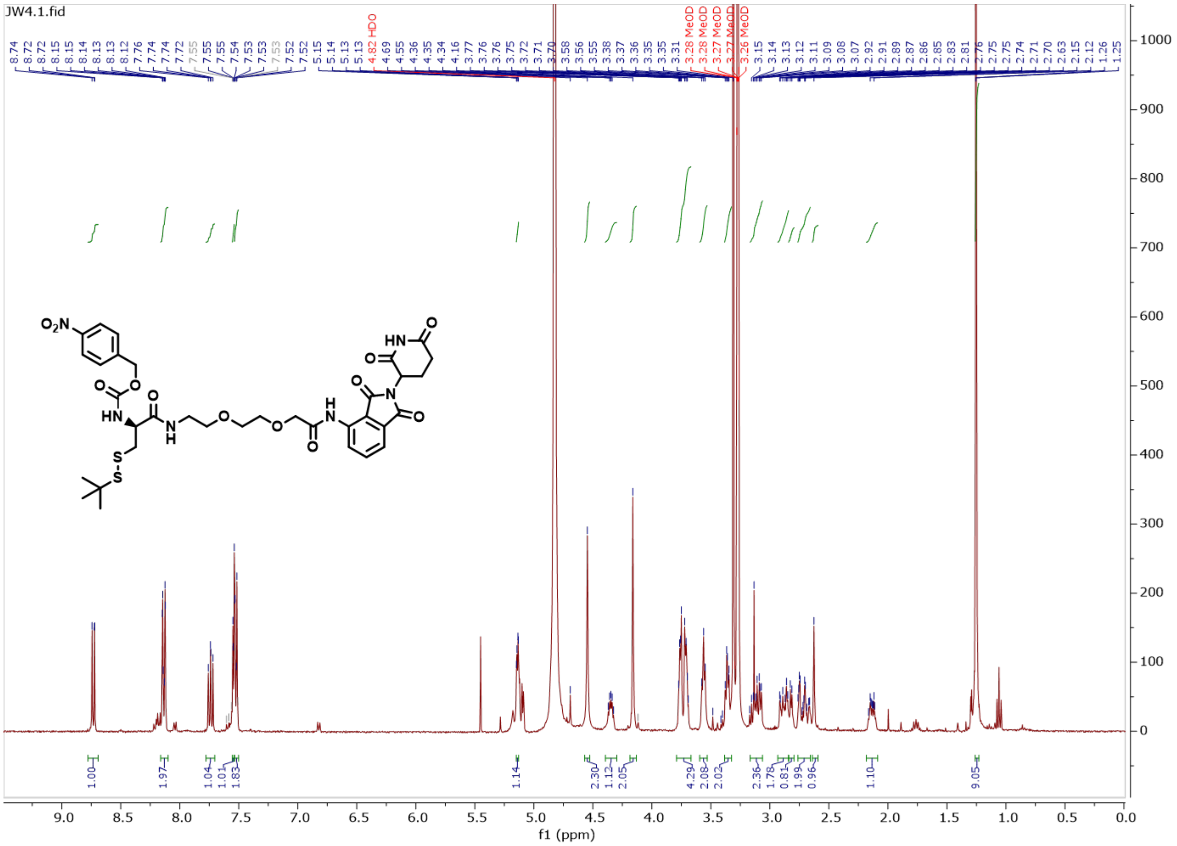
JW4.1.fid
8.74 8.72 8.72 8.15 8.15 8.14 8.13 8.13 8.12 7.76 7.74 7.74 7.72 7.55 7.55 7.55 7.54 7.53 7.53 7.53 7.52 7.52 5.15 5.14 5.13 5.13
4.82 HDO
4.69 4.55 4.36 4.35 4.34 4.16 3.77 3.76 3.76 3.75 3.72 3.71 3.70 3.58 3.56 3.55 3.38 3.37 3.36 3.35 3.35 3.31
3.28 MeOD 3.28 MeOD 3.27 MeOD 3.27 MeOD 3.26 MeOD
3.15 3.14 3.13 3.12 3.11 3.09 3.08 3.07 2.92 2.91 2.89 2.87 2.86 2.85 2.83 2.81 2.76 2.75 2.75 2.74 2.71 2.70 2.63 2.15 2.12 1.26 1.25
1.00 1.97 1.04 1.01 1.83 1.14 2.30 1.12 2.05 4.29 2.08 2.03 2.36 1.78 0.81 1.99 0.96 1.10 9.05
f1 (ppm)

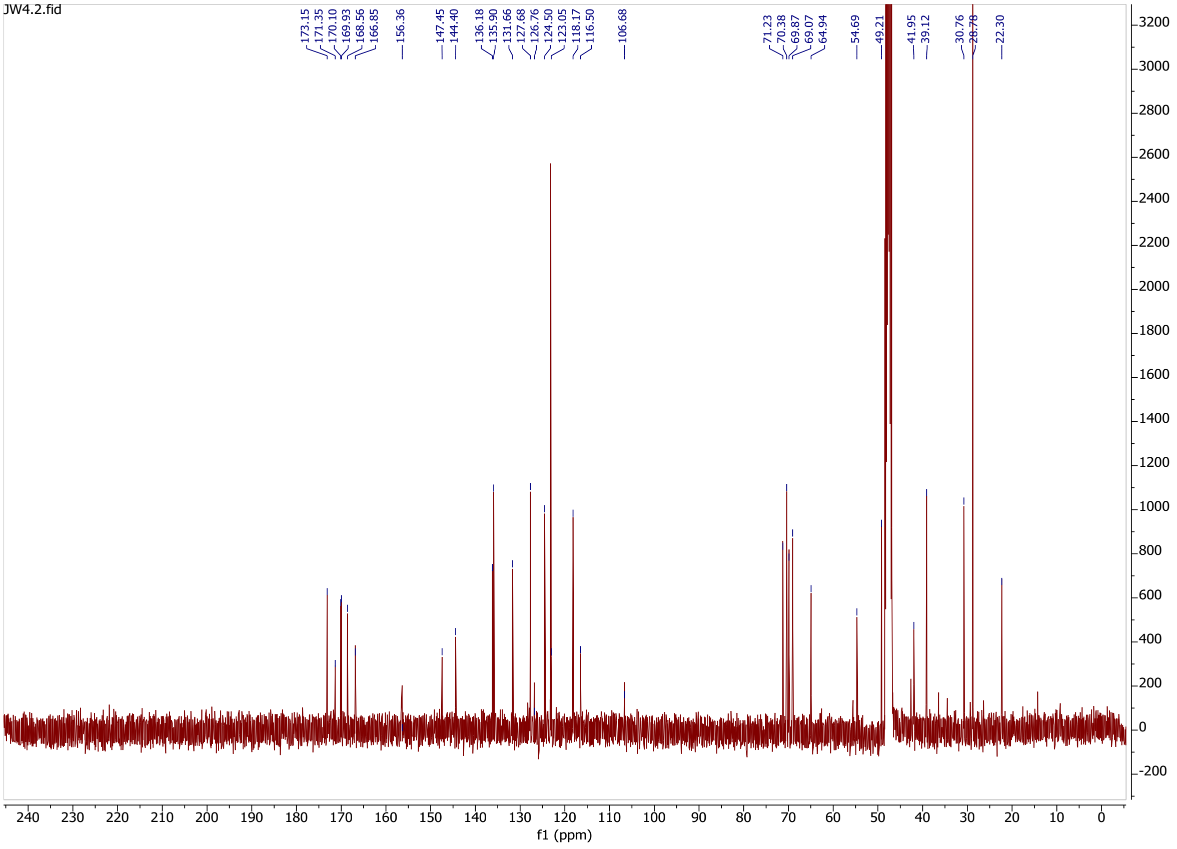
JW4.2.fid
173.15 171.35 170.10 169.93 168.56 166.85 156.36 147.45 144.40 136.18 135.90 131.66 127.68 126.76 124.50 123.05 118.17 116.50 106.68 71.23 70.38 69.87 69.07 64.94 54.69 49.21 41.95 39.12 30.76 28.78 22.30
f1 (ppm)

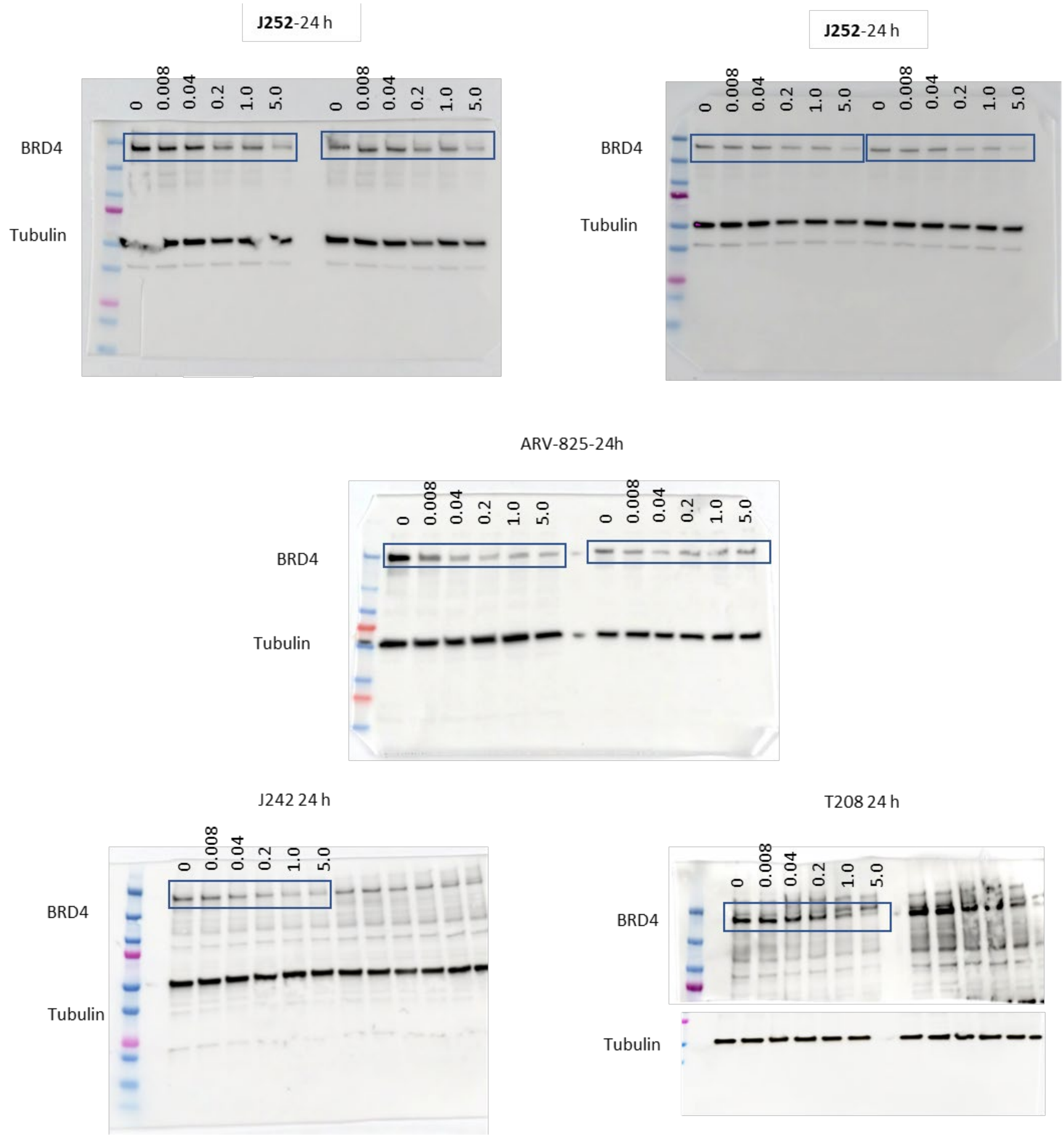

Source data 1. Unprocessed Western blots for in Figure 3, S3 and the replications.

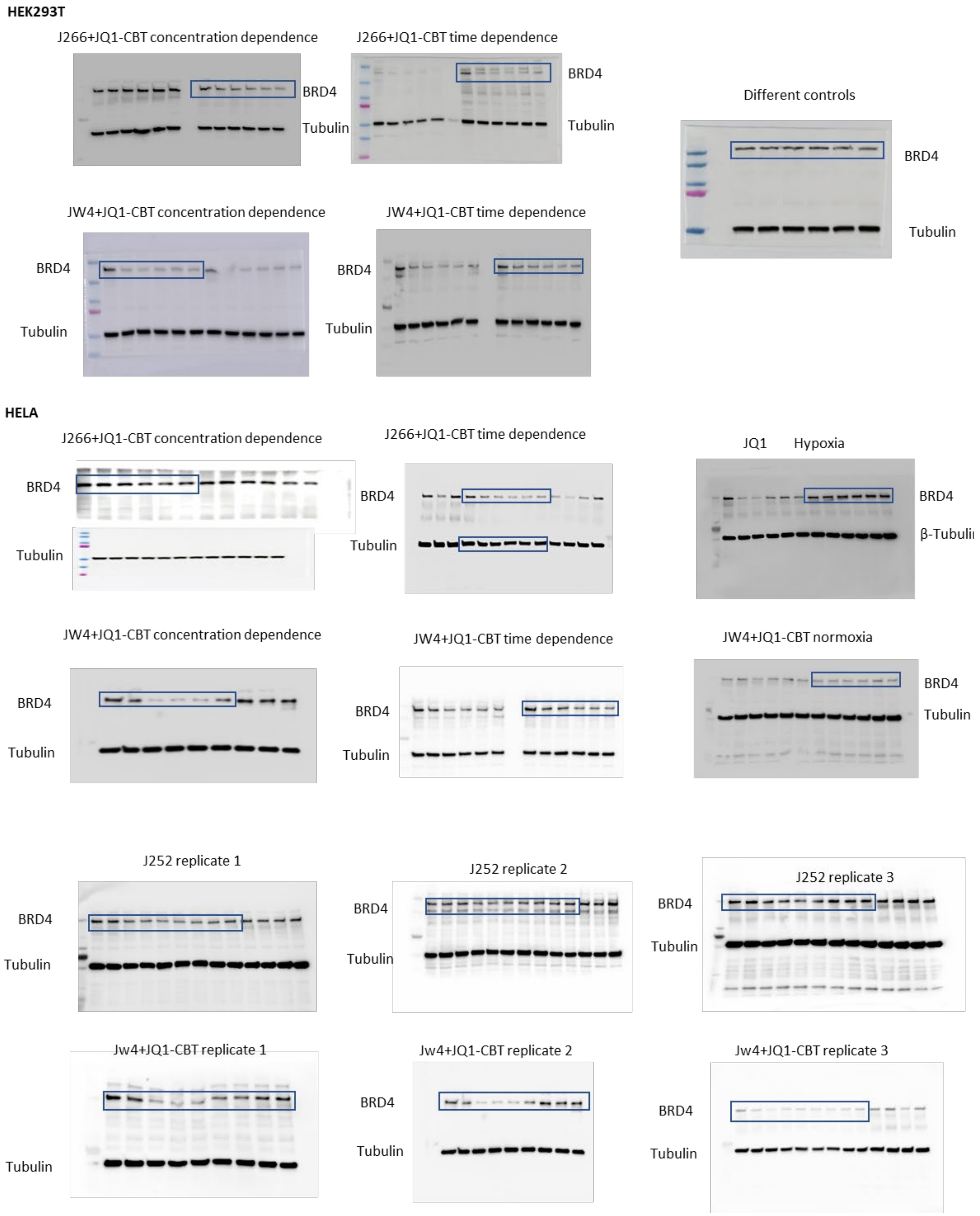

Source data 2. Unprocessed Western blot for Figure 4.
